# Myeloid cell‐based delivery of IFN‐γ reprograms the leukemia microenvironment and induces anti‐tumoral immune responses

**DOI:** 10.15252/emmm.202013598

**Published:** 2021-08-30

**Authors:** Adele Mucci, Gabriele Antonarelli, Carolina Caserta, Francesco Maria Vittoria, Giacomo Desantis, Riccardo Pagani, Beatrice Greco, Monica Casucci, Giulia Escobar, Laura Passerini, Nico Lachmann, Francesca Sanvito, Matteo Barcella, Ivan Merelli, Luigi Naldini, Bernhard Gentner

**Affiliations:** ^1^ San Raffaele Telethon Institute for Gene Therapy (SR‐TIGET) IRCCS San Raffaele Scientific Institute Milan Italy; ^2^ Vita‐Salute San Raffaele University Milan Italy; ^3^ Innovative Immunotherapies Unit Division of Immunology, Transplantation, and Infectious Diseases IRCCS San Raffaele Scientific Institute Milan Italy; ^4^ Department of Pediatric Pneumology, Allergology and Neonatology Hannover Medical School Hannover Germany; ^5^ Pathology Unit IRCCS San Raffaele Hospital Milan Italy; ^6^ National Research Council Institute for Biomedical Technologies Segrate Italy; ^7^ Hematology and Bone Marrow Transplantation Unit IRCCS San Raffaele Hospital Milan Italy

**Keywords:** *ex vivo* gene therapy, immunotherapy, interferon‐gamma, leukemia, Tie2‐expressing monocytes, Cancer, Immunology

## Abstract

The immunosuppressive microenvironment surrounding tumor cells represents a key cause of treatment failure. Therefore, immunotherapies aimed at reprogramming the immune system have largely spread in the past years. We employed gene transfer into hematopoietic stem and progenitor cells to selectively express anti‐tumoral cytokines in tumor‐infiltrating monocytes/macrophages. We show that interferon‐γ (IFN‐γ) reduced tumor progression in mouse models of B‐cell acute lymphoblastic leukemia (B‐ALL) and colorectal carcinoma (MC38). Its activity depended on the immune system's capacity to respond to IFN‐γ and drove the counter‐selection of leukemia cells expressing surrogate antigens. Gene‐based IFN‐γ delivery induced antigen presentation in the myeloid compartment and on leukemia cells, leading to a wave of T cell recruitment and activation, with enhanced clonal expansion of cytotoxic CD8^+^ T lymphocytes. The activity of IFN‐γ was further enhanced by either co‐delivery of tumor necrosis factor‐α (TNF‐α) or by drugs blocking immunosuppressive escape pathways, with the potential to obtain durable responses.

## Graphical abstract

IFN‐γ gene therapy is safe and reduces tumor progression in mouse models of B‐cell acute lymphoblastic leukemia and colorectal carcinoma. Its effects are immune‐mediated through antigen‐presentation and reprogramming of the tumor microenvironment.

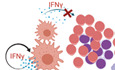

**The paper explained**

**Problem**

Cancer is the second cause of death in developed countries. The immunosuppressive microenvironment surrounding tumor cells represents a key cause of treatment failure. Therapies aiming at reprogramming and restoring anti‐tumoral immune responses have been largely employed in recent years. However, most of these immunotherapies impact on one single immune compartment.

**Results**

IFN‐γ polarizes immune responses towards anti‐tumoral states, but systemic delivery is associated with major side effects. Gene‐based, tumor‐specific IFN‐γ delivery exploiting tumor‐associated macrophages avoids systemic side effects while augmenting antitumor immune responses. Local delivery of IFN‐γ reduced tumor progression by inducing antigen presentation in the myeloid compartment and on leukemia cells, leading to T cell recruitment and activation. The efficacy of this strategy could be enhanced by combination with therapies targeting additional components of the immune system.

**Impact**

We developed a strategy to safely and locally deliver IFN‐γ into the tumor microenvironment and induce antigen presentation and antigen‐specific responses. This therapy led to the reactivation of anti‐tumoral immunity, counteracting one of the leading causes of immune escape and treatment failure. Moreover, our approach may be applied to virtually all malignancies, including solid tumors, as IFN‐γ represents a key player of immunity in most cancer types.

## Introduction

The tumor microenvironment (TME) has become of central interest to identify novel therapeutic targets. TMEs promote tumor growth and progression through several mechanisms, including polarization of host immunity to prevent anti‐cancer immune responses (Binnewies *et al*, 2018). Thus, recent work has focused on restoring the endogenous capacity of the immune system to recognize and eliminate malignant cells (Riley *et al*, 2019), particularly with immunotherapy strategies that activate anti‐tumoral T cell responses (Ghirelli & Hagemann, 2013; Motz & Coukos, 2013). One component of the immune system exerting several fundamental pro‐tumoral functions is represented by tumor‐associated macrophages (TAM) (Quatromoni & Eruslanov, 2012; Caux *et al*, 2016; DeNardo & Ruffell, 2019). In particular, a specific subpopulation of pro‐tumoral macrophages characterized by the expression of the angiopoietin receptor TIE2 (TIE2‐expressing monocytes ‐TEM) has been identified and associated with proangiogenic as well as immunosuppressive activities (Lewis *et al*, 2007; Pucci *et al*, 2009). Such population has been employed in a gene therapy‐based approach to achieve reversion of the immunosuppressive TME, as their turnover from bone marrow (BM) progenitors allowed TEMs to be exploited as vehicles delivering interferon‐α (IFN‐α) following transplantation of genetically‐engineered hematopoietic stem and progenitor cells (HSPC) (De Palma *et al*, 2008). Local expression of IFN‐α at the tumor site was associated with a general reprogramming of the immune infiltrate towards a pro‐inflammatory rather than anti‐inflammatory polarization state. That led to reduced tumor burden through multiple mechanisms, including decreased angiogenesis and metastatic seeding, restored antigen presentation capacity, and improved T cell priming and effector functions (Escobar *et al*, 2014, 2018; Catarinella *et al*, 2016). A phase I/II clinical trial using the IFN‐α gene therapy approach started in patients affected by glioblastoma multiforme (NCT03866109). Some of the therapeutic effects, such as enhancement of antigen presentation, are indirectly mediated through induction of IFN‐γ expression. IFN‐γ is principally expressed by natural killer and T cells engaging in a positive feedback loop with macrophages, which on their side release pro‐inflammatory cytokines such as interleukin‐12 (IL‐12), increase phagocytosis, and upregulate the antigen presentation machinery, thereby favoring the deployment of adaptive immunity (Alspach *et al*, 2019). IFN‐γ is a critical stabilizer of a T‐helper 1 phenotype in CD4^+^ T cells and confers cytolytic capabilities to CD8^+^ T cells (Alspach *et al*, 2019). Early studies have shown that IFN‐γ‐insensitive mice developed tumors more rapidly than wild‐type (WT) controls (Kaplan *et al*, 1998). Since then, IFN‐γ signaling, both in tumor cells and in the TME, has emerged as a pivotal effector of antitumor immunity during the “elimination” and “equilibrium” phase of cancer immunoediting and correlates positively with clinical responses to immunotherapies (Ivashkiv, 2018; Alspach *et al*, 2019). With type‐I interferons, tumor necrosis factor (TNF)‐α, and some interleukins (e.g., IL‐12), IFN‐γ is a top cytokine candidate to stimulate antitumor immunity. We hypothesized that gene‐based, tumor‐specific IFN‐γ delivery exploiting TEMs augmented antitumor immune responses while avoiding side effects associated with systemic administration. In an aggressive leukemia mouse model, we show that IFN‐γ gene therapy boosted anti‐leukemia immune responses, resulting in the elimination of more immunogenic subclones expressing surrogate antigens and selecting for more primitive disease variants that escape immune control. As chronic IFN‐γ exposure induced counterregulatory responses undermining its efficacy, we have shown that combination therapies improve therapeutic efficacy.

## Results

### Gene therapy‐based delivery of IFN‐γ and TNF‐α

**Figure EV1:**
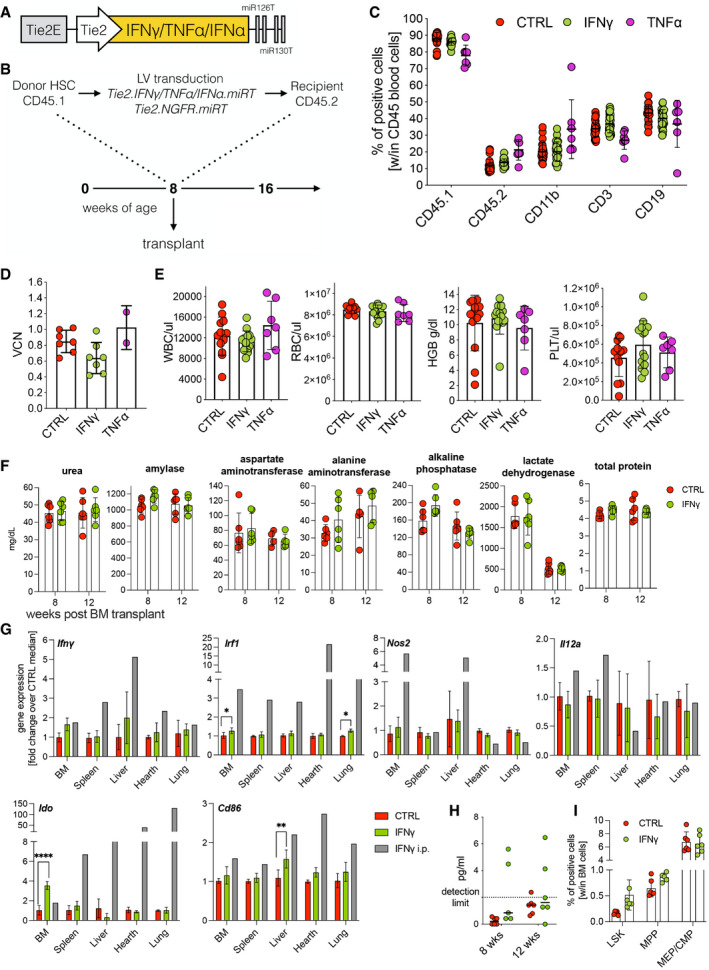
IFN‐γ and TNF‐α gene therapy: engraftment and safety AThird‐generation SIN lentiviral vector expression cassette driven by a Tie2 enhancer/promoter (Tie2E, Tie2) and post‐transcriptionally regulated by two couples of target sites for microRNA‐126 and ‐130a (miR‐126T, miR‐130aT).BExperimental design of genetically‐modified HSPC transplants followed by challenge with B‐ALL.CEngraftment of CD45.1 donor cells and lineage composition in the blood, as assessed by flow cytometry (mean ± SD, each dot represents an individual mouse, two experiments, CTRL = 12 mice, IFN‐γ = 14 mice, TNF‐α = 7 mice).DVector copy number (VCN) in peripheral blood at 8 weeks post‐transplantation (mean ± SD, each dot represents a pool of 2–3 mice, two experiments).EFrom left to right: white blood cell count (WBC), red blood cell count (RBC), hemoglobin concentration (HGB), and platelet count (PLT) measured by hemocytometer (mean ± SD, each dot represents an individual mouse, two experiments, CTRL = 12 mice, IFN‐γ = 14 mice, TNF‐α = 7 mice).F–IMice were transplanted with Tie2.NGFR‐ (CTRL) (*n* = 6) or Tie2.IFN‐γ‐ (IFN‐γ) (*n* = 6) transduced Lin‐ cells and evaluated for potential IFN‐γ‐related toxicity. (F) Serum biochemistry at 8 and 12 weeks after transplantation (mean ± SD, each dot represents an individual mouse). (G) Gene expression levels of *Ifnγ* and IFN‐γ‐related genes in several organs at steady state at 12 weeks after transplantation (mean ± SD; *P = 0.0182 for BM, *P = 0.0129 for lung, **P = 0.0044, ****P ≤ 0.0001, ordinary two‐way ANOVA) The gray bar shows a single mouse that was systemically (i.p., intraperitoneally) injected with recombinant murine IFN‐γ. (H) IFN‐γ protein quantification (ELISA) in the plasma of transplanted mice (each dot represents an individual mouse). (I) Bar graphs showing the frequency of HSPC subpopulations in the BM. LSK (CD45^+^ Lin^−^ Sca1^+^ CD117^+^ CD34^−^ CD150^+^), MPP (CD45^+^ lin^−^ Sca1^+^ CD117^+^ CD34^+^), MEP/CMP (CD45^+^ lin^−^ Sca1^−^ CD117^+^); mean ± SD, each dot represents an individual mouse.

To evaluate the antitumor potential of cytokines in the context of our HSPC gene therapy platform (De Palma *et al*, 2008; Escobar *et al*, 2014), lineage‐negative HSPC from CD45.1 donor mice were transduced with lentiviral vectors expressing either mouse IFN‐γ, TNF‐α, or a biologically inactive variant of human NGFR under the control of the Tie2e/p and microRNA 126/‐130a target sequences (Fig EV1A and B). When transplanted into lethally irradiated CD45.2 recipients, these cells, independently from the transgene, fully reconstituted hematopoiesis of the animals, with the persistence of gene‐marking *in vivo* with vector copy numbers (VCN) ranging from 0.4 to 1 (Fig EV1C and D). Conversely, transplantation of cells, where these cytokines were expressed from a strong myeloid‐specific promoter (SP146‐gp91), resulted in 100% lethality by day 17 (Appendix Table S1). Instead, no significant hematologic abnormalities were observed compared with controls, except a minor T cell reduction in the TNF‐α group, suggesting specificity of gene expression control by the miRNA‐regulated Tie2e/p cassette (Fig EV1C and E), confirmed by the modest up‐regulation of IFN‐γ‐responsive genes in the tissues, without altering blood biochemical parameters and with barely detectable IFN‐γ levels in the plasma of engrafted mice (Fig EV1F–H). For IFN‐γ, an in‐depth toxicity study was performed, confirming that transduced cell engraftment was stably maintained without negative impact on BM progenitor cell numbers (Fig EV1I). Necropsy with organ histopathology did not reveal abnormalities, except for an incidental finding of thymoma in a single mouse (full pathology report in Appendix Supplementary Text).

**Figure EV2:**
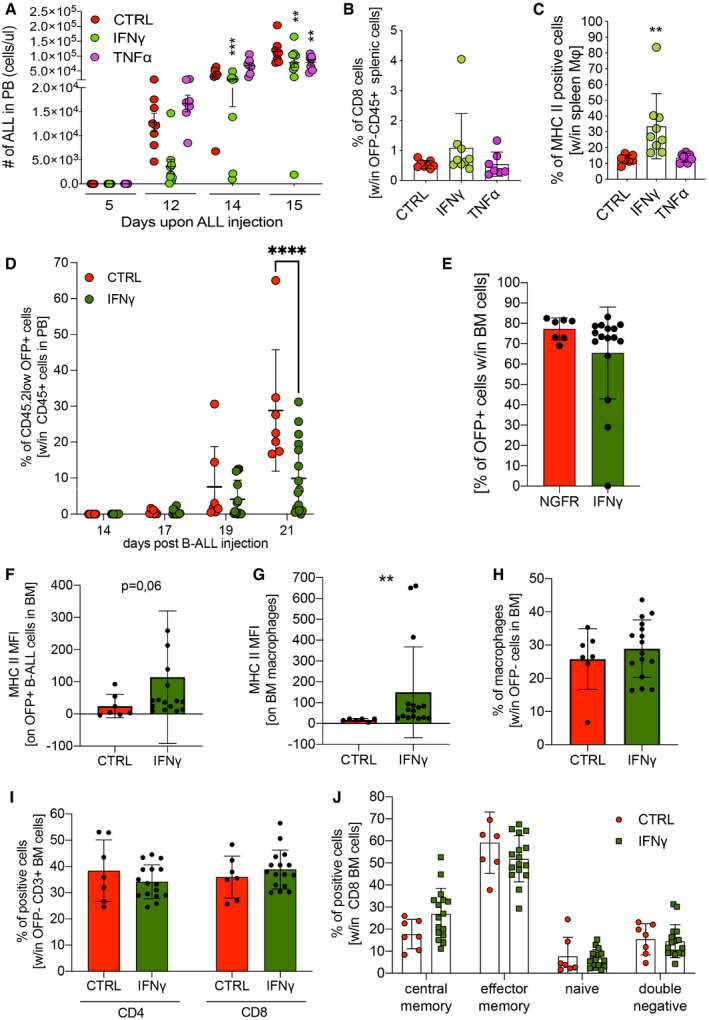
IFN‐γ, but not TNF‐α shows antitumor efficacy in miR‐126‐driven B‐ALL models A–CMice were transplanted with Tie2.NGFR‐ (CTRL, *n* = 8 mice), Tie2.IFN‐γ‐ (IFN‐γ, *n* = 9 mice) or Tie2.TNF‐α‐ (TNF‐α, *n* = 7 mice) transduced Lin^‐^ cells and challenged with B‐ALL (line#11). (A) B‐ALL progression (line#11) measured as absolute number of OFP^+^ cells in the peripheral blood of transplanted mice (mean ± SD, each dot represents an individual mouse; **P = 0.0027 CTRL vs. IFN‐γ and **P = 0.0063 CTRL vs. TNF‐α, ***P = 0.0003, two‐way ANOVA). (B) Percentage of CD8^+^ T lymphocytes within OFP^−^ CD45^+^ splenic cells (mean ± SD, each dot represents an individual mouse). (C) Percentage of MHC class II‐positive macrophages (Mφ), identified by F4/80 expression, in the spleen (mean ± SD, each dot represents an individual mouse; **P = 0.0063, ordinary one‐way ANOVA).D–JMice were transplanted with Tie2.NGFR‐ (CTRL, *n* = 7 mice) or Tie2.IFN‐γ‐ (IFN‐γ, *n* = 16 mice) transduced Lin^‐^ cells, and challenged with an independently generated B‐ALL clone (line#8). (D) B‐ALL progression (line#8) in peripheral blood measured as the percentage of CD45.2^low^ OFP^+^ cells within CD45^+^ cells (mean ± SD, each dot represents an individual mouse; ****P ≤ 0.0001, ordinary two‐way ANOVA with Geisser–Greenhouse correction). (E) B‐ALL burden in the BM measured as the percentage of CD45.2^low^ OFP^+^ cells within CD45^+^ cells (mean ± SD, each dot represents an individual mouse). (F) Bar graphs showing MFI of MHC II on OFP^+^ B‐ALL cells in the BM (mean ± SD, each dot represents an individual mouse, *P* = 0.06, Mann–Whitney test). (G) Bar graphs showing the mean fluorescence intensity of MHC II on BM macrophages (identified as CD45.1^+^ OFP^−^ CD11b^+^ F4/80^+^ cells) (mean ± SD, each dot represents an individual mouse; **P ≤ 0.01, Mann–Whitney test). (H) Bar graphs showing the percentage of macrophages within OFP^−^ cells in the BM (mean ± SD, each dot represents an individual mouse; no significant differences were revealed by Mann–Whitney test). (I) Bar graphs showing the percentage of CD4^+^ or CD8^+^ T lymphocytes within OFP^−^ CD3^+^ cells in the BM (mean ± SD, each dot represents an individual mouse; no significant differences were revealed by Mann–Whitney test). (J) Distribution of lymphocyte maturation stages within OFP^−^ CD8^+^ T cells in the BM (mean ± SD, each dot represents an individual mouse; no significant differences were revealed by Mann–Whitney test).

**Figure 1:**
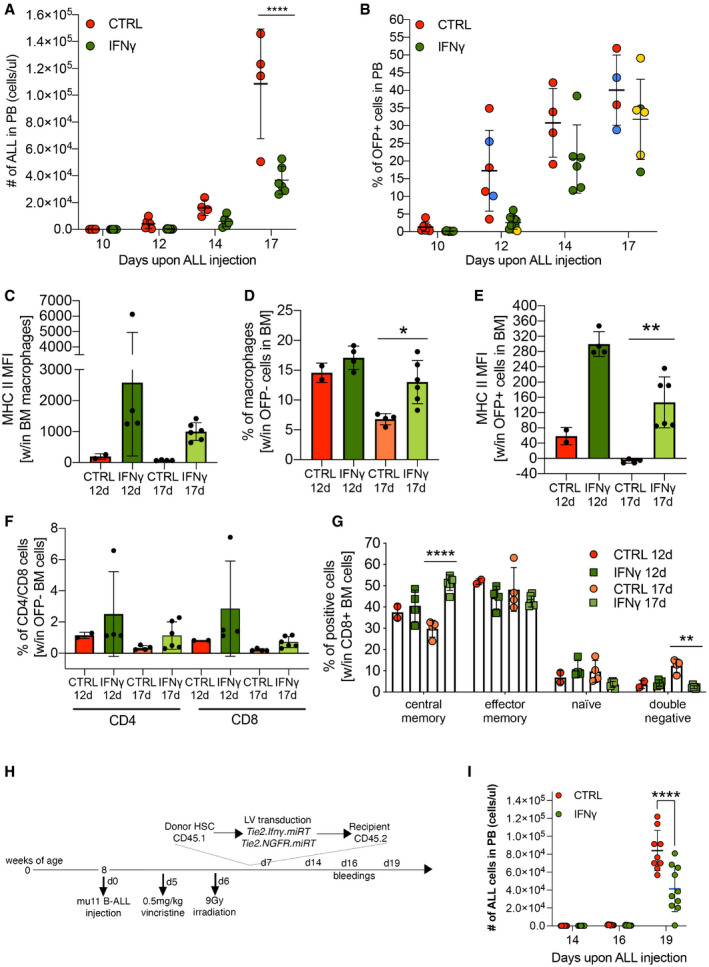
B‐ALL growth in the presence of gene therapy‐based delivery of IFN‐γ A, BB‐ALL progression measured as (A) absolute number or (B) percentage of OFP^+^ cells in the peripheral blood of transplanted mice (mean ± SD, each dot represents an individual mouse, CTRL = 6 mice, IFN‐γ = 10 mice; ****P ≤ 0.001, two‐way ANOVA; mice in blue and yellow (B) were also analyzed by single‐cell RNA sequencing; two additional independent experiments are shown in Fig EV2A).C, DMean fluorescence intensity (MFI) of MHC II on macrophages (C) and their percentage (D) within the BM immune infiltrate at 12 and 17 days after B‐ALL injection (mean ± SD, each dot represents an individual mouse; *P = 0.0145, two‐way ANOVA).EMFI of MHC class II on OFP^+^ B‐ALL cells at the experimental endpoint in the BM of transplanted mice, 12 and 17 days post‐leukemia injection (mean ± SD, each dot represents an individual mouse; **P ≤ 0.01, two‐way ANOVA).F, GPercentage of CD4^+^ and CD8^+^ T cells within OFP^−^ BM cells (F) and maturation state (G) of CD8^+^ lymphocytes (CD62L^−^CD44^−^ double negative, CD62L^−^CD44^+^ effector memory, CD62L^+^CD44^−^ naive and CD62L^+^CD44^+^ central memory T cells) in the BM immune infiltrate at 12 and 17 days after B‐ALL challenge (mean ± SD, each dot represents an individual mouse; **P = 0.005, ****P ≤ 0.001, two‐way ANOVA).HExperimental overview of a therapeutic B‐ALL model. Replicate experiments are shown in Fig EV3A and B.IAbsolute numbers of B‐ALL cells in the peripheral blood (PB) of CTRL‐ and IFN‐γ‐treated animals at the indicated time‐point (mean ± SD, each dot represents an individual mouse, CTRL = 9 mice, IFN‐γ = 10 mice; ****P ≤ 0.001, two‐way ANOVA). Statistical analyses of panels (A) and (I) are shown in Appendix Tables S4 and S5, respectively.

Transplanted animals were then challenged with a B‐ALL (line #11), which has previously been generated by inducing overexpression of miR‐126 in HSPCs (Nucera *et al*, 2016) and has extensively been characterized in the context of IFN‐α gene therapy (Escobar *et al*, 2018). While TNF‐α gene therapy had limited efficacy, IFN‐γ gene therapy showed a substantial initial reduction in leukemia development, followed by leukemia progression in most animals (Fig EV2A). Initial leukemia control in the IFN‐γ group was associated with an incremental increase in CD8^+^ T lymphocytes and a significant increase in MHC II^+^ macrophages in the spleen (Fig EV2B and C). Encouraged by these data, we replicated the efficacy of IFN‐γ in reducing tumor burden (Fig 1A and B) and evaluated its effects on BM cells at different time‐points after B‐ALL administration. Similar to splenic macrophages, IFN‐γ strongly upregulated MHC II on BM macrophages (Fig 1C), which were maintained in numbers on 17 days at significantly higher frequencies than control (Fig 1D). Interestingly, IFN‐γ induced an initial up‐regulation of MHC II on B‐ALL cells (Fig 1E). Diversely from the leukemia‐associated myeloid compartment, MHC II expression on B‐ALL cells was reduced at the late time‐point, potentially indicating leukemia‐specific escape mechanisms. Some mice from the IFN‐γ group showed increased proportions of BM T cells at day 12, which decreased to a much lower extent at disease progression on day 17 compared to control (Fig 1F). Within CD8^+^ T cells, the central memory subset was more represented in the IFN‐γ group (Fig 1G).

The effects of IFN‐γ gene therapy were recapitulated with an independently generated B‐ALL disease (line #8; Fig EV2D). Of note, some of the treated animals had drastically lower levels of leukemia at the experimental endpoint, with one animal showing barely detectable levels of leukemic cells, both in the peripheral blood and in the BM (Fig EV2E). We, again, observed increased MHC II expression within the BM microenvironment, and a trend towards more macrophages and central memory CD8^+^ T cells (Fig EV2F–J). Furthermore, in a solid tumor model of colorectal carcinoma, tumor size was significantly reduced in the IFN‐γ group (Appendix Fig S1A), and so was the tumor weight (Appendix Fig S1B). IFN‐γ induced an increase in CD8^+^ T lymphocytes (Appendix Fig S1C) and a concomitant reduction in CD11b^+^ myeloid cells within the TME (Appendix Fig S1D).

**Figure EV3:**
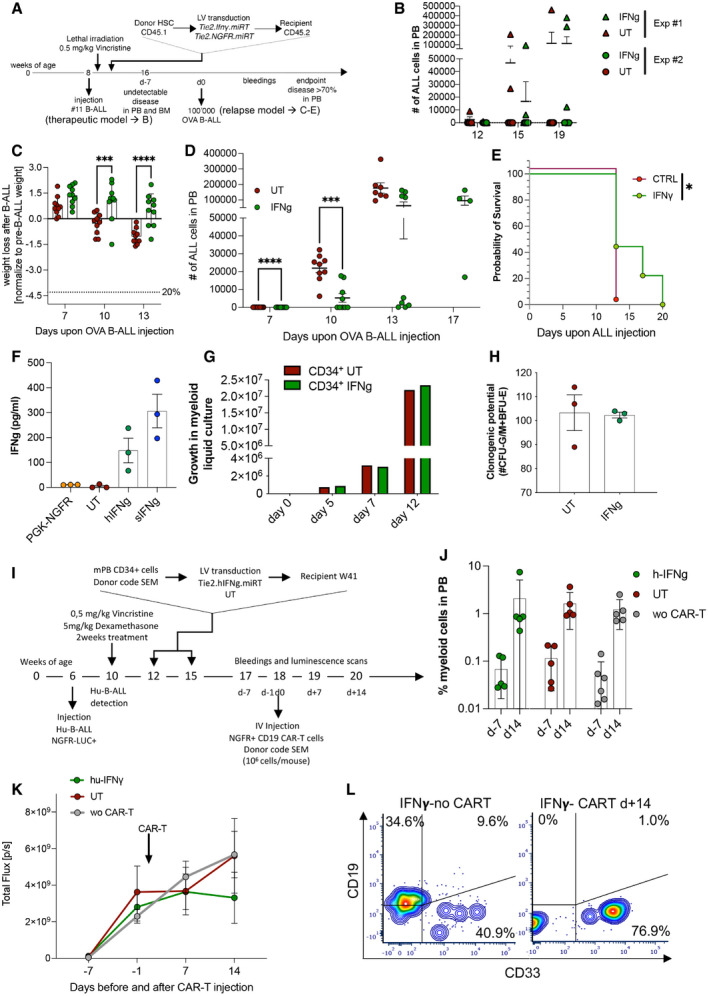
IFN‐γ gene therapy exerts anti‐leukemia effects in a relapse B‐ALL model and can be implemented in a humanized setting AExperimental design. First part (therapeutic model): female CD45.2 C57 mice were injected with B‐ALL (line#11). After 3 days, mice were treated with a single dose of 0.5 mg/kg vincristine. On day 4, mice were lethally irradiated, followed by transplantation of Tie2‐IFN‐γ‐transduced or untransduced CD45.1 lin^−^ cells the day after. Leukemic burden was monitored by serial blood draws. Second part (relapse model): mice that were tumor‐free 8 weeks (day‐7) after initial B‐ALL injection (confirmed by sentinel BM aspirate) were rechallenged with 1 × 10^5^ B‐ALL cells from an NGFR^+^/OVA^+^ subclone of disease#11 (OVA B‐ALL) and followed for leukemia growth over time. The experimental endpoint was defined as the first demonstration of leukemia burden above 70% in the blood, or clinical signs of suffering.BLeukemia burden in the therapeutic B‐ALL model (merge of two replicate experiments: Exp#1, IFN‐γ = 6 mice, CTRL = 5 mice; Exp#2, IFN‐γ = 10 mice, CTRL = 10 mice). Absolute counts of CD45.2^low^CD19^+^ blasts in the blood are shown. Note that, at the 12 and 15 week time‐points, there was a trend toward lower disease burden in the IFN‐γ group (mean ± SD, each dot represents an individual mouse).C–ELeukemia‐free mice from Exp#2 were injected with OVA B‐ALL (IFN‐γ = 10 mice, UT = 10 mice). (C) Weight loss over time after OVA B‐ALL injection (mean ± SD, each dot represents an individual mouse; ***P ≤ 0.001, ****P ≤ 0.0001, two‐way ANOVA). (D) Leukemia burden in the relapse B‐ALL model (mean ± SD, each dot represents an individual mouse; ***P ≤ 0.001, ****P ≤ 0.0001, two‐way ANOVA). (E) Survival curve after OVA B‐ALL injection (one experiment, UT = 9 mice, IFN‐γ = 9 mice, one experiment, *P ≤ 0.05, log‐rank [Mantel–Cox] test).FHuman peripheral blood mononuclear cells were obtained from buffy coats (*n* = 3 donors), transduced or not with the human TIE2.IFN‐γ or a PGK.NGFR control construct in the presence of VPX, and polarized to an M2‐like phenotype in culture. As a positive control, soluble IFN‐γ protein (sIFN‐γ) was used. The levels of human IFN‐γ were quantified by ELISA (mean ± SD).GHuman CD34^+^ cells (single donor) were transduced or not with the human TIE2.IFN‐γ construct (vector copy number: 0.81) and cultured for 2 weeks in myeloid differentiating conditions at appropriate cell densities. The growth curve indicates no negative impact from transduction with the TIE2.IFN‐γ construct.HHuman CD34^+^ cells (single donor) were transduced or not with the human TIE2.IFN‐γ construct (vector copy number: 0.81, as per point G) and cultured for 2 weeks in methylcellulose. Clonogenic potential (number of CFU‐G/M or BFU‐E per 3‐cm plate) is shown, indicating no negative impact from transduction with the TIE2.IFN‐γ construct (three technical replicates, mean ± SEM).ISchematic representation of a therapeutic, humanized B‐ALL model. NSGW41 mice were injected with a luciferase‐marked, primary human B‐ALL (week 6). After detection of B‐ALL by bioluminescence imaging (week 10), two cycles of vincristine/dexamethasone chemotherapy were administered as induction treatment. Mice were then transplanted with two doses of 1 × 10^6^ CD34^+^ HSPC, transduced or not with the human TIE2.IFN‐γ lentiviral vector (weeks 12 and 15). Disease progression was measured on week 17 (2 and 5 weeks after CD34^+^ cell transplants, respectively). Not surprisingly, there were no differences between experimental groups, as relevant CD34^+^ HSPC engraftment levels may not be reached until 6 weeks after transplant. To contain B‐ALL growth, a single dose of 1 × 10^6^ CD19 CAR‐T cells (autologous to the CD34^+^ graft) was administered (week 18‐ day 0), and mice were followed by periodic bleeding and bioluminescence imaging until they developed CAR‐T‐related complications (after day+14).JHuman myeloid cell engraftment in the blood before (day‐7) and after CD19 CAR‐T cell injection (day+14), estimated as the fraction of human CD45^+^/NGFR^−^/CD19^−^ cells in the blood. Note that both the CD19 CAR‐T cells and the B‐ALL are marked by NGFR. There were no differences between human myeloid engraftment of mock‐transduced, TIE2.IFN‐γ‐transduced or non‐CAR‐T‐treated mice. Engraftment increased to therapeutically relevant levels at day+14 (mean ± SD, each dot represents an individual mouse; h‐IFN‐γ/CAR‐T = 5 mice, UT/CAR‐T = 5 mice, w/o CAR‐T = 6 mice, three of which from the UT and three from the h‐IFN‐γ group).KMonitoring of B‐ALL burden by bioluminescence imaging (mean ± SEM; h‐IFN‐γ/CAR‐T = 5 mice, UT/CAR‐T = 5 mice, w/o CAR‐T = 6 mice, three of which from the UT and three from the h‐IFN‐γ group). Please note a trend toward lower disease burden on day+14 in the CD19 CAR‐T cell‐treated mice from the TIE2.IFN‐γ group, providing an initial proof of concept that this treatment may be active in a clinically relevant, human leukemia model. Future studies will employ T cell preparations that do not cause xenogeneic graft‐versus‐host disease, thereby allowing longer follow‐up.LRepresentative FACS plots (gated on hCD45^+^NGFR^−^ cells) of mice transplanted with TIE2.IFN‐γ‐transduced CD34^+^ cells and treated or not with CD19 CAR‐T cells on day+14. Note that CD19 CAR‐T cell treatment induced human B‐cell aplasia, as expected.

To approach a more clinically relevant experimental model, we tested the efficacy of IFN‐γ gene therapy in a therapeutic setting. Mice challenged with line #11 B‐ALL received chemoradiotherapy for disease control and transplant conditioning, and were then infused with gene‐modified lineage‐negative HSPCs (Fig 1H). Importantly, in this therapeutic setting, IFN‐γ gene therapy resulted in significant leukemia growth inhibition compared to control animals (Fig 1I). In replicate experiments, where vincristine chemotherapy and irradiation were given earlier after B‐ALL injection (Fig EV3A), most animals were cured from leukemia in both IFN‐γ and control groups (Fig EV3B). To model B‐ALL relapse, mice surviving the first challenge were then injected with a B‐ALL subclone of line #11 (NGFR^+^/Ovalbumin^+^). Mice from the IFN‐γ gene therapy group showed a significant delay in relapse kinetics (Fig EV3C), which translated into improved clinical condition (Fig EV3D) and prolonged survival (Fig EV3E).

To further confirm clinical translatability, a humanized TIE2.IFN‐γ construct was designed, validated for functionality and absence of toxicity on human culture‐derived M2 macrophages and CD34^+^ HSPC, and tested in a therapeutically relevant model of human B‐ALL, in combination with CD19 CAR‐T cells (Fig EV3F–L).

### IFN‐γ effects on leukemia growth are immune‐mediated

**Figure EV4:**
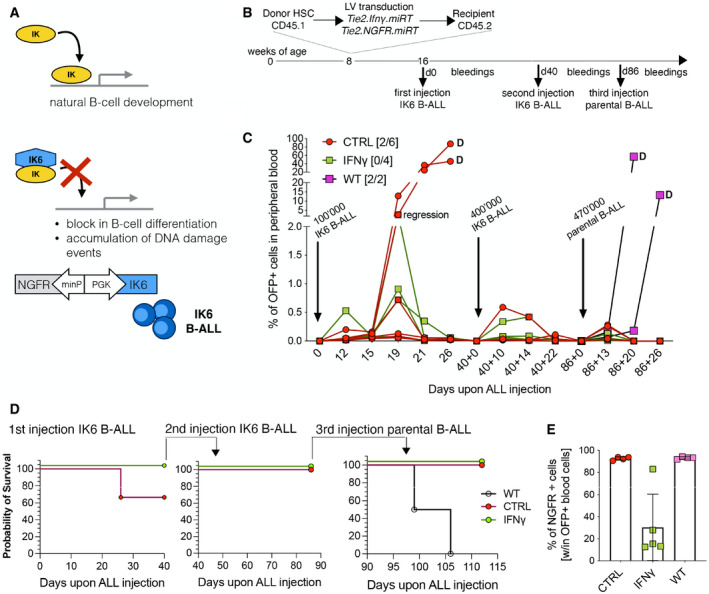
IFN‐γ gene delivery induces antigen loss in IKAROS6 expressing B‐ALL Schematic representation of IKAROS6 activity in leukemia, and genetic modification of the parental B‐ALL to induce constitutive expression of the dominant‐negative IK6 protein.Schematic representation of the experimental outline.B‐ALL progression in the peripheral blood, measured as the percentage of OFP^+^ cells within total white blood cells. CTRL, mice engrafted with HSPCs transduced with the Tie2e/p.NGFR vector; IFN‐γ, mice engrafted with HSPCs transduced with the Tie2e/p.IFN‐γ vector; WT CTRL, sub‐lethally irradiated mice that did not receive prior BM transplantation. The numbers in [brackets] indicate the fraction of mice (over the total number) that developed disease upon primary challenge; differences were not significant by Fisher's exact test. Arrows indicate successive disease challenges, with the indicated B‐ALL subline.Survival curves of three re‐challenges with IK6 B‐ALL (challenges #1 and #2) and parental line #8 disease (challenge #3; one experiment, CTRL = 6 mice, IFN‐γ = 4 mice, WT = 2 mice).Expression of NGFR on OFP^+^ leukemic cells after the first challenge with IK6 B‐ALL (mice from (C), mean ± SD).

Next, we challenged the mice with a B‐ALL subline that expresses the dominant‐negative variant of IKAROS1 (IKAROS6‐IK6) in line #8 (B‐ALL #8‐IK6) (Nucera *et al*, 2016). IK6 is found in human B‐ALL and promotes B‐cell differentiation block, progenitor proliferation, and DNA damage accumulation (Fig EV4A) (Nakase *et al*, 2000; Tonnelle *et al*, 2001; Sezaki *et al*, 2003; Kano *et al*, 2008; Iacobucci *et al*, 2012). B‐ALL #8‐IK6 is expected to be more immunogenic than the parental line, due to the expression of human antigens (IK6, co‐expressed with dNGFR marker gene) and the potential accumulation of novel tumor‐specific antigens. The leukemia #8‐IK6 grew similarly to the parental #8 line in sub‐lethally irradiated animals (Nucera *et al*, 2016). Instead, when BM‐reconstituted mice were injected with #8‐IK6 (Fig EV4B), more CTRL mice died of B‐ALL compared with IFN‐γ (4/12 CTRL and 2/13 IFN‐γ in two separate experiments; Fig EV4C and D). Interestingly, only IFN‐γ‐treated mice showed loss of the NGFR antigen co‐expressed with IK6 on B‐ALL cells (Fig EV4E). All the mice that survived the first challenge became resistant to further challenges from both high doses of #8‐IK6 B‐ALL, as well as its parental #8 B‐ALL, suggesting active immunization against leukemia‐associated antigens (Fig EV4D). Taken together, these data indicate that IFN‐γ gene therapy facilitates the deployment of immune responses directed against leukemia‐associated antigens.

**Figure 2:**
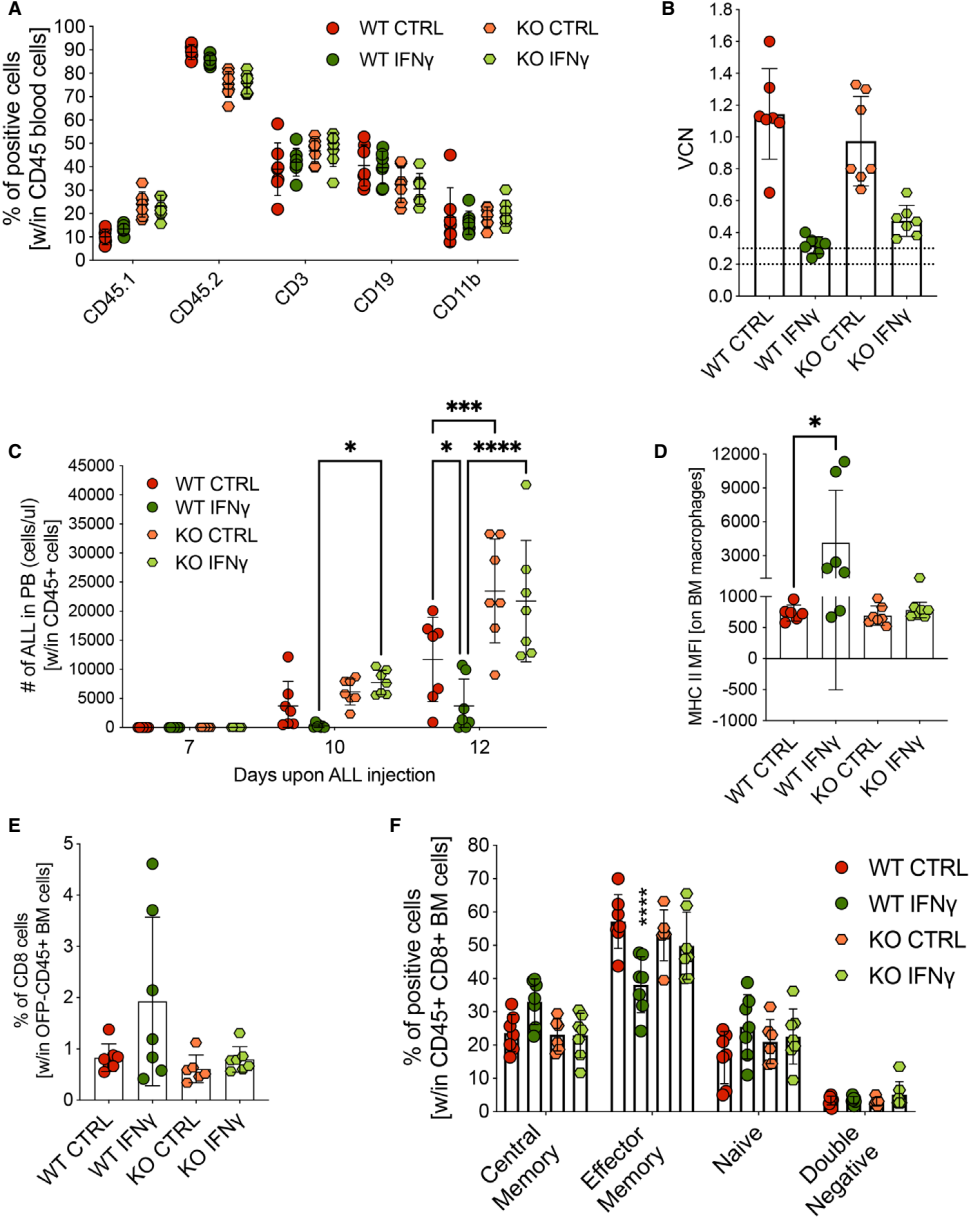
IFN‐γR1 knockout HSPCs display loss of IFN‐γ anti‐tumoral activity Engraftment of CD45.2 donor cells and lineage composition following transplantation of wild‐type (WT) or IFN‐γ receptor 1 knockout (KO) HSPCs transduced with either the control Tie2.NGFR (CTRL) or Tie2.IFN‐γ (IFN‐γ) LV (mean ± SD, each dot represents an individual mouse, WT CTRL = 7 mice, WT IFN‐γ = 7 mice, KO CTRL = 7 mice, KO IFN‐γ = 7 mice).Vector copy number (VCN) in peripheral blood at 8 weeks post‐transplantation (mean ± SD, each dot represents an individual mouse).B‐ALL progression measured as absolute number of OFP^+^ cells in the peripheral blood (mean ± SD, each dot represents an individual mouse; *P = 0.0286 at day 10; *P* = 0.0174 at day 12; ***P = 0.0002; ****P ≤ 0.0001; ordinary two‐way ANOVA).MFI of MHC class II‐positive macrophages, identified by F4/80 expression, in the BM (mean ± SD, each dot represents an individual mouse; WT IFN‐γ vs. WT CTRL: *P = 0.0221, Mann–Whitney test).Percentage of CD8^+^ T lymphocytes within OFP^−^ CD45^+^ BM cells (mean ± SD, each dot represents an individual mouse).Maturation state (CD62L^−^CD44^−^ double negative, CD62L^−^CD44^+^ effector memory, CD62L^+^CD44^−^ naive and CD62L^+^CD44^+^ central memory T cells) of CD8^+^ lymphocytes (mean ± SD, each dot represents an individual mouse; WT IFN‐γ vs. WT CTRL: ****P ≤ 0.0001, ordinary one‐way ANOVA). Data information: Statistical analysis of panel (C) is shown in Appendix Table S6.

To further confirm the role of the immune system in mediating the effects of IFN‐γ gene therapy, we transduced IFN‐γ receptor 1 knockout (IFN‐γR1 KO) HSPCs with control or IFN‐γ‐expressing vectors and transplanted them, in parallel to WT CD45.2 HSPCs, into lethally irradiated CD45.1 recipients. After confirmed hematopoietic reconstitution (Fig 2A), mice were challenged with B‐ALL (line #11). Even though *in vivo* VCN was lower than in previous experiments (0.3–0.4) for mice transplanted with IFN‐γ‐transduced cells (Fig 2B), WT‐transplanted animals receiving IFN‐γ gene therapy manifested reduced disease growth. In contrast, this effect was absent when mice were transplanted with IFN‐γR1 KO cells transduced with the IFN‐γ vector. Furthermore, IFN‐γR1 KO‐transplanted animals showed higher levels of disease compared with WT‐transplanted animals (Fig 2C), probably due to lack of response to endogenous IFN‐γ. This lack of IFN‐γ efficacy was accompanied by the absence of changes within BM populations, including MHC II expression (Fig 2D), CD8^+^ T lymphocytes (Fig 2E), and memory T cell subpopulations (Fig 2F), which were instead observed in animals transplanted with IFN‐γ‐transduced WT cells. Therefore, we demonstrated that the anti‐tumoral activity of IFN‐γ is dependent mainly on the capacity of the immune cells to respond to its stimulation, as the lack of the IFN‐γR1 on hematopoietic cells led to loss of efficacy.

### Single‐cell RNA sequencing unveils an initially broad antitumor response, followed by loss of IFN‐γ responses and selection of more aggressive B‐ALL variants

**Figure 3:**
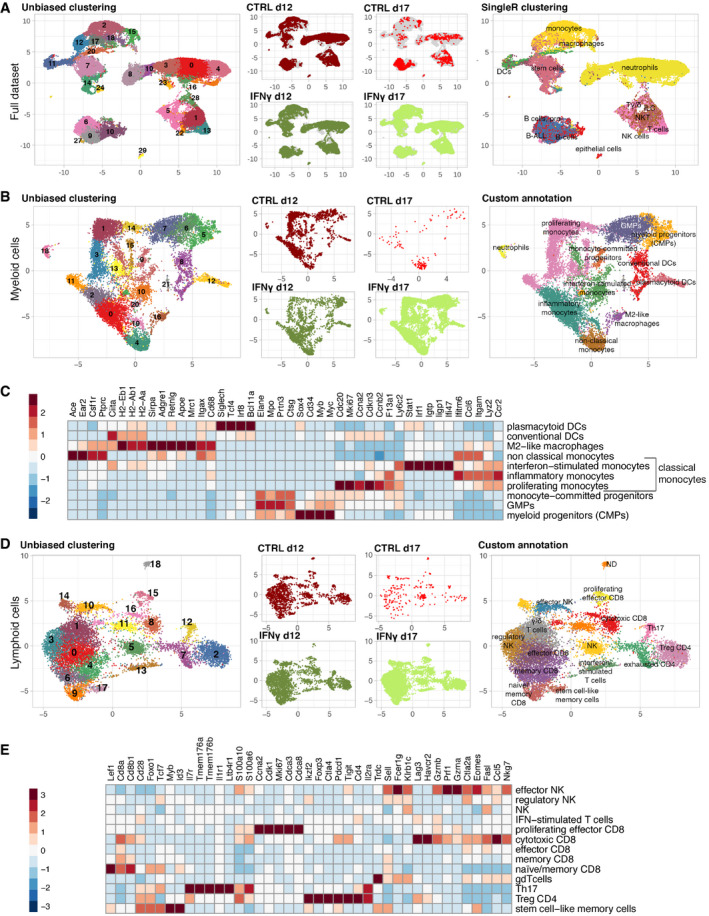
Identification of immune populations by scRNAseq of the BM leukemic environment scRNAseq was performed on sorted CD45^+^ Annexin V^‐^ BM cells from a pool of two control mice and two IFN‐γ‐treated mice at day 12, and on a pool of two control and two pools of two IFN‐γ‐treated mice on day 17 (blue and yellow dots in Fig 1B indicate the individual mice from the CTRL and IFN‐γ groups, respectively).
Uniform manifold approximation and projection (UMAP) visualization of the complete dataset. The left panel shows cells colored according to unsupervised clustering (Louvain—resolution 0.6); the middle panel shows the distribution of cells according to experimental conditions, whereas the right panel displays cell type classification according to singleR immgen annotation, as described in Materials and Methods.UMAP visualization of the myeloid compartment. From the left to the right panel, cells are colored according to unsupervised clustering (Louvain—resolution 1.2), experimental condition and marker‐based, custom cell‐type classification.Heatmap showing top marker genes (obtained from unsupervised clustering) used for custom classification of the myeloid compartment. Interferon‐stimulated, inflammatory and proliferating monocytes belong to the group of classical monocytes.UMAP visualization of the lymphoid compartment. From the left to the right panels, cells are colored according to unsupervised clustering (Louvain—resolution 1.2), experimental condition and custom marker‐based cell‐type classification.Heatmap showing top marker genes (obtained from unsupervised clustering) used for custom classification of cells that belong to the lymphoid compartment.

**Figure EV5:**
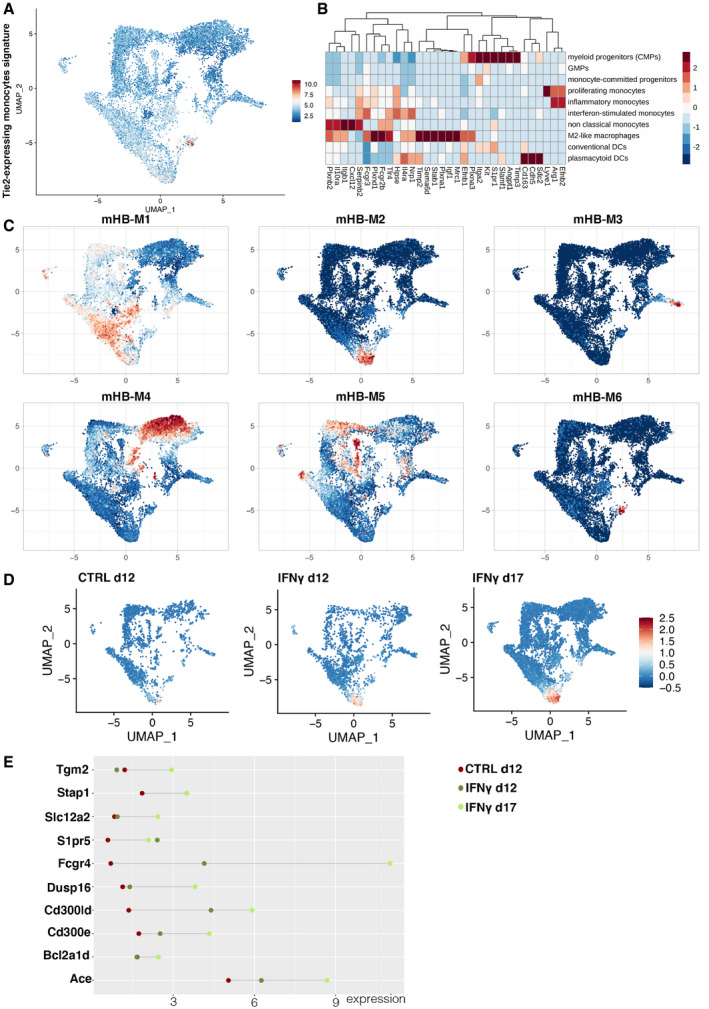
Identification of Tie2‐expressing monocytes and non‐classical monocytes by scRNAseq analysis UMAP highlighting cells expressing the signature of TEMs, based on the transcriptional signature published by Pucci *et al* (2009).Heatmap showing the expression of the genes from the TEM signature within the different myeloid populations defined by our custom signature (see Fig 3C).Mapping of the myeloid cell signatures identified by Witkowski *et al* (2020) onto the myeloid compartment of our leukemic mice. Note that the pro‐leukemic mHB‐M2 cluster colocalizes with our subset of non‐classical monocytes.Representation of the pro‐leukemic mHB‐M2 cluster across the different experimental conditions. Note that this cluster becomes more represented in the IFN‐γ group on day 17.Differential expression of mHB‐M2 marker genes in the myeloid cells of our treated animals, according to experimental group.

Next, we undertook an unbiased transcriptional characterization of the B‐ALL immune microenvironment performing 10× single‐cell RNA sequencing (scRNAseq) on total BM cells from mice treated or not with IFN‐γ gene therapy (controls indicated as blue dots, IFN‐γ as yellow dots in Fig 1B). Quality control metrics are summarized in Table EV1. Unsupervised clustering identified many transcriptional states representative of the major cell types present in BM, with SingleR classification revealing a broad representation of the different hematopoietic cell types, including B‐ALL, myeloid, and lymphoid clusters (Fig 3A and Table EV2). In detail, the unsupervised and custom analysis revealed subpopulations within the myeloid (Fig 3B and C) and lymphoid (Fig 3D and E) compartments. Moreover, we detected a small cluster of M2‐like macrophages expressing a characteristic TEM signature (Fig EV5A and B) (Pucci *et al*, 2009), as well as a distinct subset of non‐classical monocytes (mHB‐M2) that have recently been associated with disease progression in B‐ALL (Fig EV5C and D) (Witkowski *et al*, 2020). Notably, the mHB‐M2 subset was overrepresented in IFN‐γ‐treated animals at day 17, indicating a possible mechanism of therapy resistance (Fig EV5D and E).

To understand what cell populations responded to IFN‐γ, we interrogated myeloid‐ or lymphoid‐tailored IFN‐γ‐related gene expression signatures. Dendritic cells, M2‐like macrophages, and interferon‐stimulated macrophages showed highest IFN‐γ signature expression, which increased over baseline in the IFN‐γ group. At the same time, little response was observed in non‐classical, inflammatory, and proliferating monocytes (Appendix Fig S2A). In addition, gene therapy induced a broad IFN‐γ response in most T‐/NK cell subsets on day 12, which was lost in a fraction of cells on day 17 (Appendix Fig S2B).

**Figure 4:**
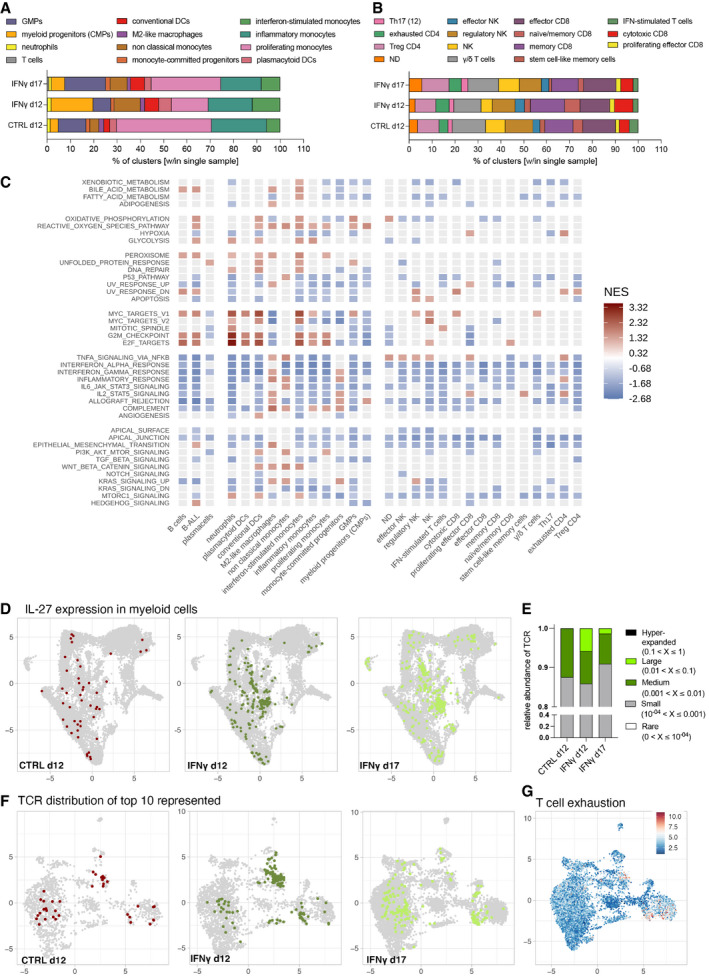
Single‐cell analysis of IFN‐γ‐driven changes in BM cells and TCR repertoire of marrow infiltrating lymphocytes Myeloid cell type distribution. The bar plots show the relative contribution (% of cells out of total) of custom cell type classification labels (see Fig 3B) within each experimental condition.Lymphoid cell type distribution. The bar plots show the relative contribution (% of cells out of total) of custom cell type classification labels (see Fig 3D) within each experimental condition.Gene Set Enrichment Analysis (GSEA) using hallmarks gene sets, comparing the day 17 IFN‐γ with the day 12 IFN‐γ condition. GSEA was performed on logFC pre‐ranked gene lists obtained from day 17 vs. day 12 gene expression comparisons within each cell type (custom classification). Red and blue tiles denote positive and negative Normalized Enriched Scores (NES), respectively. Gray tiles show terms that resulted not enriched from the GSEA test (cut‐off *P*.adj < 0.2).UMAPs showing the amount and distribution of IL‐27‐expressing myeloid cells (UMI > 0), colored according to experimental condition.Relative TCR abundance within all sequenced TCRs represented as small (0.01–0.1%), medium (0.1–1%) or large (1–10%) clusters. No hyperexpanded TCRs (> 10% abundance) were detected.UMAPs showing cells that harbor the top 10 abundant TCRs for each condition, obtained from TCR repertoire analysis. Cells are colored according to experimental condition.UMAP showing a T cell exhaustion signature in the lymphoid compartment. Cells are colored according to the module score value of the signature (see methods for gene lists), scaled across an 11‐value color‐palette (inverted RdBu palette from RColorBrewer package—http://colorbrewer.org).

Next, we compared the relative representation of the different subpopulations in the sequenced samples. IFN‐γ‐treated animals showed increased myeloid progenitors (CMPs + GMPs), dendritic cells and, surprisingly, non‐classical monocytes at the expense of proliferating monocytes (Fig 4A), as well as an increase in cytotoxic CD8^+^ T lymphocytes on day 12 (Fig 4B). To shed light on the mechanisms governing loss of IFN‐γ efficacy, we performed gene set enrichment analysis (GSEA) using the hallmark gene set from the Molecular Signatures Database (MSigDB) on the pre‐ranked gene list obtained from the comparison of day 17 vs. day 12 (Fig 4C). With the notable exception of M2‐like macrophages, which increased their inflammatory profile on day 17, all other cell populations downregulated inflammatory hallmarks (including IFN‐α and IFN‐γ response) while upregulating gene sets related to energy metabolism, cell cycle, and proliferation (Fig 4C and Tables [Link], [Link], [Link]). Given that the M2‐like macrophage cluster highly resembles TEMs (Fig EV5A and B), it is likely that this population continues to express IFN‐γ from our vector construct resulting in autocrine stimulation. However, the small proportion of TEM in the leukemia microenvironment may not be sufficient to prevent B‐ALL progression. Notably, we see widespread induction of IL‐27, a T cell pro‐survival factor (Schneider *et al*, 2011), in myeloid cells from the IFN‐γ group, persisting to day 17 (Fig 4D).

To assess the impact of myeloid‐derived IFN‐γ release on the T cell compartment, we performed single cell TCR sequencing of BM‐infiltrating T cells (Fig 4E and F). On day 12, the most represented TCRs showed a higher clonal abundance within IFN‐γ‐treated mice than controls. Most T cell clones expressing highly represented TCRs in IFN‐γ‐treated mice at day 12 map onto the cytotoxic CD8 cluster (Fig 4F), possibly representing tumor‐specific T cells that may help in early leukemia control, but became unable to persist while leukemia progressed, as evidenced by downregulation of transcriptional programs associated with the transcription factor *Tox* (Table EV5, cytotoxic CD8). CD4^+^ T cells and some cytotoxic CD8^+^ T cells showed expression of T cell exhaustion markers (Fig 4G), but with barely any differences between the CTRL and IFN‐γ groups.

**Figure 5:**
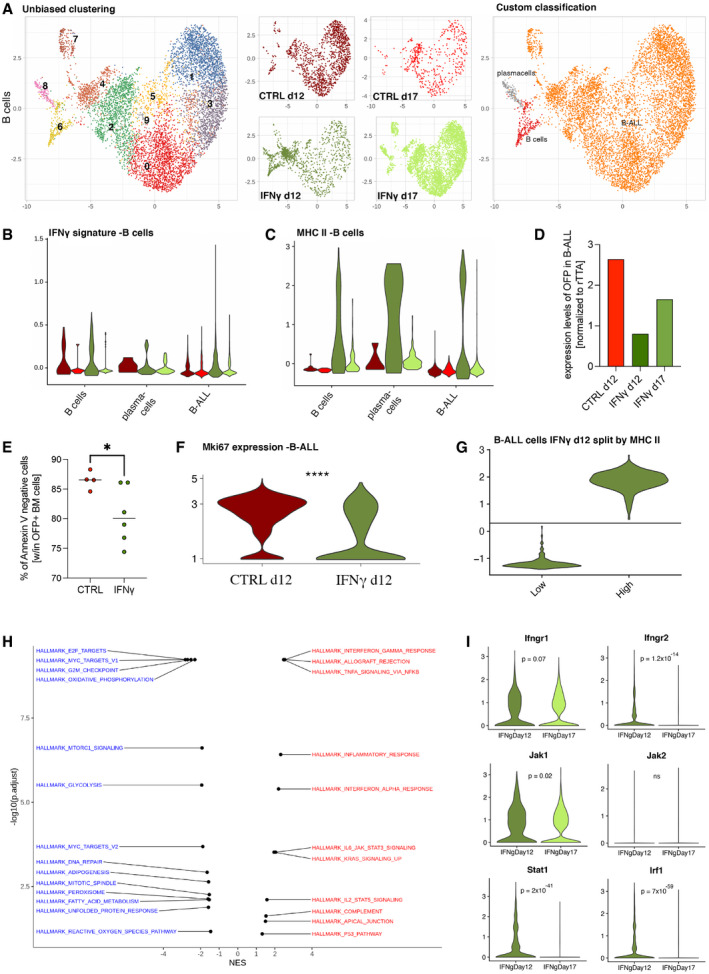
IFN‐γ‐driven changes in B‐ALL cells and possible mechanisms of efficacy loss AUMAP visualization of the B‐cell/B‐ALL compartment. From the left to the right panel, cells are colored according to unsupervised clustering (Louvain—resolution 0.6), experimental condition and marker‐based custom cell‐type classification, unequivocally identifying leukemic cells by the presence of the tTA transcript.B, CViolin plots show the distribution of IFN‐γ (B) and MHC II gene (C) signatures (module score value for each cell—see gene list details in methods) for each experimental condition, within each custom cell type. Wilcoxon rank‐sum test for IFN‐γ signature, condition IFN‐γ d12 vs. CTR d12: B‐ALL *P* = 2.68 × 10^−19^, B cells *P* = 0.5, plasma cells *P* = 0.78; Wilcoxon rank‐sum test for MHC II signature, condition IFN‐γ d12 vs. CTRL d12: B‐ALL *P* = 4.26 × 10^−46^, B cells *P* = 1 × 10^−9^, plasma cells *P* = 0.32.DReduction of OFP expression in B‐ALL cells in the IFN‐γ‐treated vs. CTRL group (mean).EViability of OFP^+^ B‐ALL cells before sorting for single‐cell RNA sequencing (mean, CTRL = 4 mice, IFN‐γ = 6 mice; *P = 0.0333, Mann–Whitney test).FExpression of Mki67 transcripts in B‐ALL cells from CTRL d12 and IFN‐γ d12 conditions, extracted from the scRNAseq dataset (****P ≤ 0.0001, Welch's test).G, H(G) IFN‐γ d12 B‐ALL cells were split according to MHC II gene signature values and compared for gene expression profile, in order to identify significantly enriched terms (within the hallmark gene set MsigDB collection) by using the GSEA approach (H). On the left, hallmark terms downregulated (negative NES—blue labels) in the MHC II high leukemia cell population are shown, whereas the right shows upregulated hallmark terms (positive NES—red labels) in the MHC II high leukemia cell population, as compared to the MHC II low counterpart.ILoss of IFN‐γ response over time in B‐ALL cells from the IFN‐γ group, potentially facilitated by reduced expression of Ifngr1, Ifngr2, Jak1, Stat1‐ and Irf1 (data extracted from the scRNAseq dataset statistical analysis by two‐way ANOVA).

Similarly, B‐ALL showed transcriptional heterogeneity by unsupervised analysis (Fig 5A), with transient up‐regulation of an IFN‐γ gene signature, as well as MHC II molecules (Fig 5B and C). This was accompanied by a reduction in the potential neoantigen OFP, which is co‐expressed with the miR‐126 oncogene (Nucera *et al*, 2016) (Fig 5D). Moreover, B‐ALL cells from IFN‐γ‐treated animals displayed decreased viability and reduced expression of the proliferation marker Mki67 compared to controls (Fig 5E and F). Based on the MHC II molecule expression module score, we divided B‐ALL cells from IFN‐γ d12 into an MHC II high subpopulation, displaying enrichment for IFN‐responsive gene modules, and an MHC II low subpopulation, with higher expression of proliferative and oxidative phosphorylation pathways (Fig 5G and H). Moreover, at day 17, B‐ALL cells from treated animals showed reduced expression of the IFN‐γ receptors and signal transducers (Ifngr1, Ifngr2, and Jak1), accompanied by reduced intracellular signaling via Stat1 and Irf1 (Fig 5I), possibly indicating the development of a resistance mechanism to IFN‐γ (Arenas *et al*, 2018).

### Enhancing IFN‐γ anti‐tumoral activity by combination therapies

As the delivery of IFN‐γ by gene therapy showed promising effects on the immune microenvironment at an early time‐point when the leukemic burden was limited, but loss of efficacy on day 17, we next tested whether combination therapies were able to enhance the effectiveness of IFN‐γ gene therapy in controlling the weakly immunogenic, parental B‐ALL.

**Figure 6:**
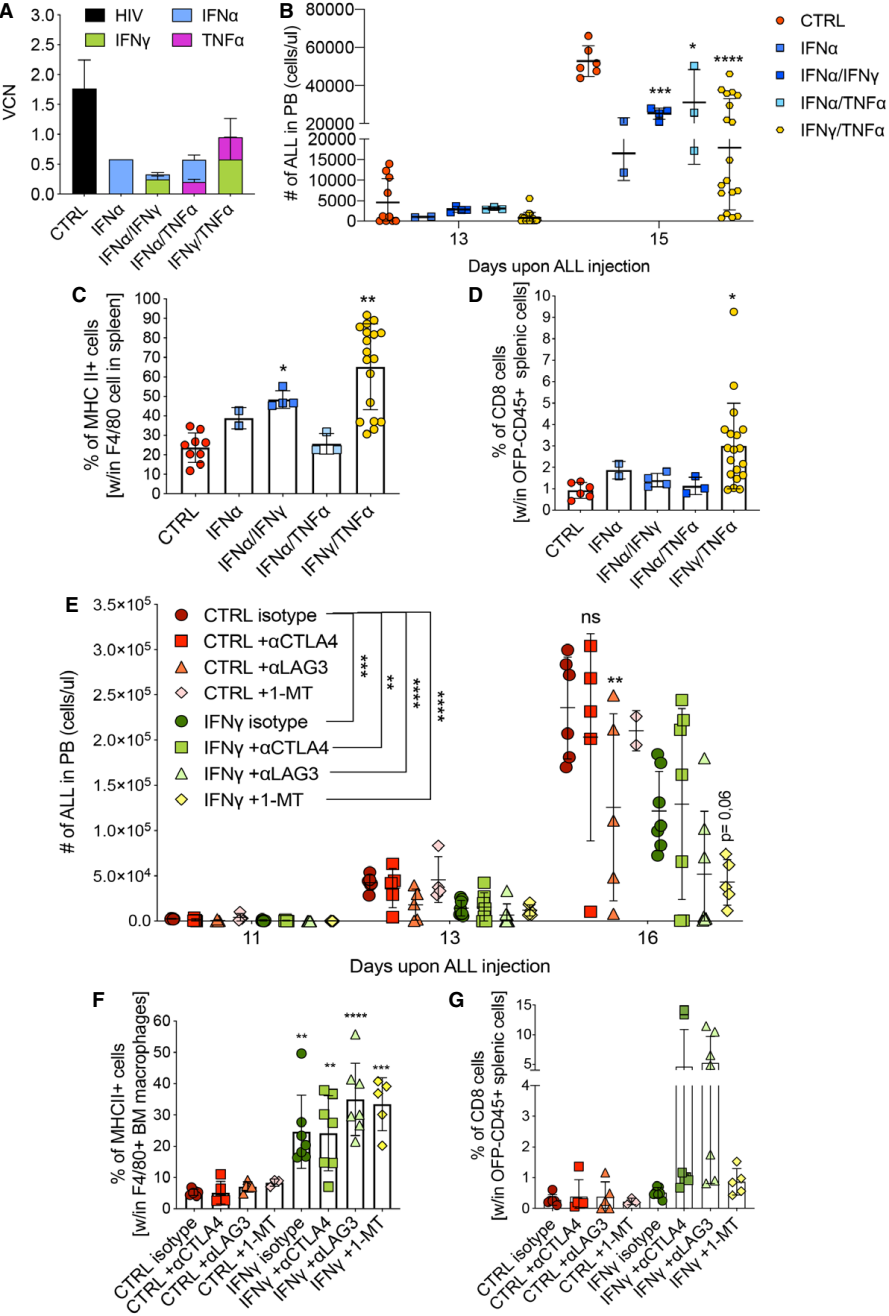
Combination of IFN‐γ gene therapy with other immunotherapies enhances anti‐tumoral activity A–DCombination gene therapy with IFN‐α, TNF‐α, and IFN‐γ, expressed from the Tie2 vector platform. CTRL (Tie2.NGFR), two independent experiments, *n* = 9 mice; IFN‐α, one experiment, *n* = 2 mice; IFN‐α/IFN‐γ, one experiment, *n* = 4 mice; IFN‐α/TNF‐α, one experiment, *n* = 3 mice; IFN‐γ/TNF‐α, two independent experiments, *n* = 19 mice. (A) Vector copy number (VCN) in peripheral blood at 8 weeks post‐transplantation (mean ± SD). (B) B‐ALL progression in peripheral blood measured as absolute number of OFP^+^ cells (mean ± SD, each dot represents an individual mouse; *P = 0.0308, ***P = 0.0004, ****P ≤ 0.0001, two‐way ANOVA with Geisser–Greenhouse correction). (C) Percentage of MHC II^+^ macrophages, identified by F4/80 expression, in the spleen (mean ± SD, each dot represents an individual mouse; *P = 0.0484, **P = 0.0049, ordinary one‐way ANOVA). (D) Percentage of CD8^+^ T lymphocytes within CD45‐positive and OFP‐negative splenic cells (mean ± SD, each dot represents an individual mouse; *P = 0.0356, ordinary one‐way ANOVA).E–GCombination of IFN‐γ gene therapy with immuno‐oncology drugs. CTRL (Tie2.NGFR) + antibody isotype, *n* = 6 mice; CTRL + αCTLA4 antibody, *n* = 5 mice; CTRL + αLAG3 antibody, *n* = 5 mice; CTRL + 1‐methyltryptophan (1‐MT), *n* = 5 mice; IFN‐γ (Tie2.IFN‐γ) + antibody isotype, *n* = 7 mice; IFN‐γ + αCTLA4 antibody, *n* = 7 mice; IFN‐γ + αLAG3 antibody, *n* = 7 mice; IFN‐γ + 1‐MT, *n* = 5 mice. (E) B‐ALL progression in the peripheral blood measured as absolute number of OFP^+^ cells (mean ± SD, each dot represents an individual mouse; ns = not significant, **P = 0.001 (CTRL isotype vs. IFN‐γ + αCTLA4); **P = 0.0022 (CTRL isotype vs. CTRL + αLAG3), ***P ≤ 0.001, ****P ≤ 0.0001; comparisons are against the corresponding isotype, or as indicated by the lines; two‐way ANOVA). (F) Percentage of MHC II^+^ macrophages, identified by F4/80 expression, in the spleen (mean ± SD, each dot represents an individual mouse; **P ≤ 0.01, ***P ≤ 0.001, ****P ≤ 0.0001 as compared to CTRL isotype, ordinary one‐way ANOVA). (G) Percentage of CD8^+^ T lymphocytes within OFP‐negative BM cells (mean ± SD, each dot represents an individual mouse). Data information: Statistical analyses of panels (C) and (E) are shown in Appendix Tables S7 and S8, respectively.

First, we multiplexed our TEM‐based gene therapy platform by co‐delivering dual combinations of IFN‐γ, IFN‐α, and TNF‐α, all expressed from the Tie2e/p‐miRT vector backbone stably integrated into HSPC. Mice transplanted with HSPC co‐transduced with IFN‐α/IFN‐γ, IFN‐α/TNF‐α or IFN‐γ/TNF‐α successfully engrafted to similar levels as control LV‐transduced cells or cells transduced only with the IFN‐α vector, resulting in average *in vivo* VCNs between 0.3 and 1.0, variably distributed between the individual cytokine components (Fig 6A). Mice were then challenged with B‐ALL (line #11). All cytokine groups showed reduced B‐ALL growth compared to control (Fig 6B). The IFN‐γ/TNF‐α combination revealed improved efficacy, with some animals showing very low levels of OFP^+^ disease until euthanasia, even at total VCNs below 1 of the combined cytokines (Fig 6B). Efficacy correlated with increased percentages of MHC II^+^ macrophages for the conditions that included IFN‐γ (Fig 6C), while the percentage of CD8^+^ T cells was exclusively enriched in the most promising combination of IFN‐γ with TNF‐α (Fig 6D).

As a second approach, given that IFN‐γ may activate a series of feedback mechanisms ultimately leading to loss of efficacy (Benci *et al*, 2019), we evaluated synergy with drugs blocking immunosuppressive escape pathways: (i) checkpoint blockers targeting inhibitory signaling cascades induced after T cell activation; (ii) inhibitors of the indoleamine 2,3‐dioxygenase (IDO), upregulated in myeloid cells upon IFN‐γ stimulation (see Fig EV1G) that blunts T cell expansion by catabolizing tryptophan in the tumor microenvironment. After hematopoietic reconstitution with IFN‐γ‐ or control‐transduced HSPC and B‐ALL challenge, we treated the mice with either monoclonal antibodies directed against the inhibitory T cell checkpoints CTLA4 (αCTLA4) or LAG3 (αLAG3), thereby reducing T‐cell exhaustion, or with the IDO inhibitor 1‐methyltryptophan (1‐MT). Checkpoint blockade led to a variable response in both IFN‐γ and control animals. Some showed a very low disease burden at the endpoint analysis, and others had disease levels comparable to the respective isotype controls (Fig 6E). Noteworthy, a higher proportion of mice from the IFN‐γ group showed a strong response compared to the control group (2/7 vs. 1/5 mice for αCTLA4; 4/7 vs. 1/5 mice for αLAG3, respectively). Mice reconstituted with control HSPC showed little disease inhibition following treatment with the IDO inhibitor (Fig 6E). Instead, mice from the IFN‐γ gene therapy group showed a strong, homogeneous response to IDO inhibition, possibly reflecting a synergistic effect (Fig 6E). While all groups receiving gene therapy‐based delivery of IFN‐γ showed higher percentages of MHC II^+^ macrophages than the controls (Fig 6F), the increase in CD8^+^ T cells was especially important in animals receiving the checkpoint blockers, and inversely correlated with disease levels (Fig 6G).

In summary, we showed that the anti‐leukemic efficacy of myeloid‐based delivery of IFN‐γ could be improved by checkpoint blockers and IDO inhibitors, at least in part by amplifying T cell responses, which may be blunted upon chronic IFN‐γ exposure.

## Discussion

We here demonstrate stable genetic engineering of mouse hematopoiesis with an *Ifn‐γ* transgene, whose expression is transcriptionally targeted to a subset of tumor‐infiltrating myeloid cells using a previously described and extensively characterized lentiviral vector platform (De Palma *et al*, 2008; Escobar *et al*, 2014, 2018). Advantages of this delivery platform include limited systemic cytokine exposure and the need for a single, one‐off treatment only, as genetically modified HSPCs guarantee persistence. We show immune‐mediated inhibition of tumor growth in two B‐ALL models representative of the human disease (Nucera *et al*, 2016) and a heterotopic colorectal cancer model, even when only 20% of hematopoietic cells carry the genetic modification, as evidenced by the *in vivo* VCN of 0.2. Our B‐ALL model rapidly induces an immunosuppressive microenvironment, including downregulation of MHC II genes, up‐regulation of IL‐10 (Escobar *et al*, 2018), and accumulation of non‐classical monocytes, which have recently been shown to play a pivotal role in B‐ALL progression (Witkowski *et al*, 2020). Similar to IFN‐α (Escobar *et al*, 2018), IFN‐γ monotherapy did not cure mice from a weakly immunogenic disease. Compared to IFN‐α, IFN‐γ led to a strong induction of MHC II molecules, presumably enhancing antigen presentation (Escobar *et al*, 2018). We noted a loss of response to IFN‐γ treatment (leukemic cells included) at the late time‐point, with only M2‐like macrophages (Fig 5A) maintaining an active IFN‐γ pathway. This argues against the loss of cells expressing transgenic IFN‐γ, expected to be prominently within M2‐like macrophages, where Tie2 promoter activity is highest. While the proportion of M2‐like macrophages appears stable over time (Fig 5A), it is relatively low, representing <5% of the mononuclear myeloid BM microenvironment, and well below the proportions found in some solid tumors (Mazzieri *et al*, 2011). Therefore, it is possible that the Tie2e/p‐miRT targeting construct is more suitable for solid tumors than hematologic malignancies affecting the BM due to a higher concentration of TEMs in the former. Interestingly, TEMs were distinct from a pro‐leukemic population of non‐classical monocytes described by Witkowski *et al*. This latter population was detected at higher frequency in the IFN‐γ‐treated animals (see Fig EV5), indicating a potential feedback mechanism activated by the leukemia to facilitate immune evasion. It is tempting to speculate that drugs specifically targeting these non‐classical monocytes (but not TEMs) may synergize with IFN‐γ gene therapy in B‐ALL and possibly other tumors. Additional mechanisms responsible for paradoxical effects of IFN‐γ on antitumor immunity have been described, most notably up‐regulation of inhibitory checkpoint molecules such as PD‐L1 or inactivation of downstream signaling components (Ribas & Wolchok, 2018). In our scRNAseq dataset, we did not find transcriptional evidence for such events in any of the non‐leukemic BM cell populations analyzed, with the presence of exhaustion markers mostly limited to CD4^+^ T cells (Fig 5G), and comparable between treatment and controls. Physiologically, IFN‐γ secretion in activated T cells peaks at 24 h, followed by a rapid drop pointing to intrinsic homeostatic mechanisms restricting excessive activation (Alspach *et al*, 2019). We hypothesize that the cytotoxic CD8^+^ T‐cell clones we observe on day 12 in the IFN‐γ group, which likely represent tumor‐reactive T cells, fail to persist. Indeed, the persistence of progenitor‐like CD8^+^ T cells has recently been linked to the expression of the *Thymocyte Selection Associated High Mobility Group Box* (*Tox*) gene (Yao *et al*, 2019). *Tox* was one of the top downregulated genes in cytotoxic CD8^+^ T cells over time upon IFN‐γ treatment (Table EV5), and the differentially expressed hallmark gene signatures (such as hypoxia, interferon‐α, E2F targets; Fig 5H) closely resemble those obtained in the work of Yao et al (2019).

We observed that IFN‐γ causes early proliferation impairment, as a direct effect on B‐ALL cells, in addition to immune‐mediated effects on the more immunogenic subclones (Fidanza *et al*, 2017). This “equilibrium phase” is followed by an “escape phase” where less immunogenic and more aggressive B‐ALL subpopulations are selected. Indeed, we found transcriptional alterations in B‐ALL cells over time, indicating activation of signaling pathways associated with immature stem cell phenotype (Dierks *et al*, 2007; Lin *et al*, 2010; Dagklis *et al*, 2015), metabolic switch to oxidative phosphorylation (Aykin‐Burns *et al*, 2009; Schafer *et al*, 2009; Kamarajugadda *et al*, 2012; Lagadinou *et al*, 2013; Jiang *et al*, 2014; Ghanbari Movahed *et al*, 2019) and increased proliferation (de Barrios *et al*, 2020) suggesting a disease‐intrinsic mechanism of resistance. Our finding that only part of the B‐ALL cells upregulates MHC II in response to IFN‐γ may point to intratumor heterogeneity, i.e., the presence of pre‐existing resistant B‐ALL subpopulations that are positively selected under treatment. Many of the pathways upregulated on day 17 in the B‐ALL overlapped with those found in the MHC II low fraction on day 12, supporting this hypothesis. Downregulation of MHC II on acute myeloid leukemia cells has recently been identified as a principal mechanism of relapse after allogeneic transplantation, which was overcome, at least *in vitro*, by IFN‐γ treatment (Toffalori *et al*, 2019). To best exploit this cytokine therapeutically, it will be relevant to further study the mechanisms and determinants of IFN‐γ responsiveness in leukemia.

In conclusion, we have shown how local delivery of IFN‐γ represents a safe and efficient strategy to achieve TME reprogramming leading to early leukemia control. In‐depth characterization of the healthy BM and leukemic compartment identified IFN‐γ's fundamental role in antitumor and antigen‐specific responses, which are lost over time through different mechanisms impinging on T cell activity and augmenting aggressiveness of the disease. This strategy could be applied to other malignancies such as solid tumors, and its effects could be enhanced by further tackling the immune evasion mechanism acquired during disease progression.

## Materials and Methods

### Plasmid construction and lentiviral vector production

The Tie2.IFN‐γ, Tie2.TNF‐α, and Tie2.NGFR lentiviral vectors (LV) were generated by cloning a murine *Ifnγ* cDNA (CAT#: MR227155, OriGene) or a murine *Tnfα* cDNA (CAT#: MR212145, OriGene) or dNGFR into Tie2 e/p‐miRT backbone (Escobar *et al*, 2014). Likewise, a human *IFNγ* cDNA was cloned into the human TIE2 e/p‐miRT backbone (Escobar *et al*, 2014). The primers used to amplify *Ifnγ* cDNA and *Tnfα* cDNA included a stop codon at the end of the coding sequence. Concentrated VSV‐G‐pseudotyped LV stocks were produced and titered as described previously (De Palma & Naldini, 2002). 293T cells were regularly tested for mycoplasma contamination.

### Vector copy number

For VCN analysis, 5 ng/µl of genomic DNA was used to perform by droplet digital PCR, as previously described (Milani *et al*, 2017), using primers/probe sets designed on the primer binding site region of LV, or on the Tie2 region and the specific cytokine, and normalized on murine endogenous DNA by a primers/probe set against the murine Sema3a gene (Appendix Table S2). The PCR was performed following manufacturer's instructions (Bio‐Rad) and read with QX200 reader. Analysis was performed with QuantaSoft Analysis Pro Software (Bio‐Rad).

### Validation of the human IFN‐γ construct

All primary cells were obtained from donors that signed informed consent forms approved by the Ospedale San Raffaele Ethics Committee, in accordance with the Declaration of Helsinki, the Department of Health and Human Services Belmont Report and the Good Clinical Practice guidelines of the International Conference on Harmonization. CD14^+^ monocytes were isolated from buffy coats obtained from healthy donors by positive selection (Miltenyi) and cultured for 9 days in RPMI 1640 (15% FBS, 5% human serum), 10 ng/ml rhM‐CSF (PeproTech), and 20 ng/ml rhIL4 (Miltenyi Biotech) from day 5. After 9 days of culture, 200 ng/ml of LPS (Sigma‐Aldrich) was added for 24 h, and IFN‐γ concentration was measured in cell culture supernatants by standard sandwich ELISA (antibody clones NIB42 and 4S.B3, BD Biosciences). LV transduction of monocytes was accomplished at a multiplicity of infection (MOI) of 10 following a 3‐h exposure to VpX‐VLP (https://doi.org/10.3389/fimmu.2020.01260). A primary t(9;22)^+^ B‐ALL was obtained from the OSR biobank following informed consent and IRB approval. The disease was transduced with a lentivirus co‐expressing NGFR and Luciferase, passaged in NSG mice, selected for NGFR positivity, and frozen. The B‐ALL was intravenously injected into NSG^W41^ mice, at a dose of 1.2 × 10^6^ cells per mouse. Disease burden was periodically monitored by bioluminescence imaging. After disease detection, mice received weekly vincristine (0.5 mg/kg i.v.) and five times per week dexamethasone (5 mg/kg, i.p.) injections, as shown in the scheme in Fig EV3. Commercially available leukapheresis was subjected to CD34 selection (CliniMACS, Miltenyi). The positive fraction was transduced with the human TIE2‐IFN‐γ construct (or mock‐transduced) as described (Petrillo *et al*, 2018), while the negative fraction was used to produce autologous CAR‐T cells directed against the CD19 antigen, as described (Casucci *et al*, 2018), with exception that a lentivirus was used for transduction. Engineered CD34^+^ and T cells were characterized *in vitro*, and cryopreserved aliquots were used for transplantation (1 × 10^6^ CD34^+^ or T cells per dose, intravenously).

### Mice

Female C57Bl/6 Ly45.2 and Ly45.1 mice of 6–8 weeks of age were purchased from Charles River Laboratory. Female C57Bl/6 Ly45.1 mice were used as donors. Female NSG^W41^ mice were obtained from internal breeding at our mouse facility. At time of treatment, animals were 6–8 weeks old. All animal procedures were performed according to protocols approved by the Animal Care and Use Committee of the San Raffaele Scientific Institute (IACUC 600, 836, 936, 1095, 1102) and communicated to the Ministry of Health and local authorities according to Italian law. Mice were housed in the two animal facilities of the San Raffaele Hospital (Dibit 1—Via Olgettina 58, 20132 Milan, Italy and Dibit2—Via Olgettina 60, 20132 Milan, Italy) in groups of 2–5 per cage, with sterilized food, water *ad libitum* and controlled 12‐h/12‐h light/dark cycles.

### IFN‐γ toxicity studies

Organs were collected from mice 12 weeks after transplant. Part of them were fixed in formalin and underwent pathology evaluation at the mouse pathology facility of Ospedale San Raffaele. Hearts, lungs, livers, spleens, and BM were collected and dry frozen at −80°C. RNA was isolated using the miRNeasy Mini Kit (QIAGEN, 217004) according to manufacturer's instructions. RNA was converted to cDNA using SuperScript™ IV VILO™ Master Mix (Thermo Fisher Scientific, 11756050). Digital droplet PCR with SsoAdvanced Universal SYBR Green Supermix (Bio‐Rad, 1725270) was employed to measure expression of selected genes. Primers were obtained from Biorad: *Ifng* Mmu EG5073430, *Cd86* MmuEG5065771, *Hprt* MmuEG5073066, *Actb* MmuEG5193531, *Irf1* MmuEG5073097, *Ido1* MmuEG5083069, *Il12a* MmuEG5079577, *Nos2* MmuEG5084795. Blood chemistry was performed on murine serum samples in the Ospedale San Raffaele Mouse Lab with the ILab Aries machine. IFN‐γ ELISA was performed on plasma with the Mouse IFN‐gamma Quantikine ELISA Kit MIF00 (R&D Systems) according to the manufacturer's instructions.

### Hematopoietic stem and progenitor cell transplantation

Bone marrow was harvested from female 6‐ to 9‐week‐old C57Bl/6 mice, and lineage‐negative HSPCs were purified by immuno‐magnetic isolation (Lineage Cell Depletion Kit mouse, Miltenyi, #130–090–858). HSPC transduction, culture, and transplantation in recipient female 8‐week‐old C57Bl/6 mice were performed as previously described (Escobar *et al*, 2014). In the therapeutic setting (Figs 1H and I and EV3A and B), mice injected with B‐ALL first received a single i.v. dose of 0.5 mg/kg Vincristine between days 3 and 5 from B‐ALL administration, followed by 900 Rad total body irradiation and infusion of transduced HSPCs.

### Tumor challenge and combination immunotherapies

For leukemia challenge, mice were intravenously injected with 5 × 10^4^ parental B‐ALL, unless differently specified in the experiment (Fig EV4B and C: 1 × 10^5^ IK6 B‐ALL, 4 × 10^5^ IK6 B‐ALL, 4.7 × 10^5^ line #8 B‐ALL; Fig EV3F–I: 1 × 10^5^ OVA B‐ALL line #11). Combination therapies were administered intraperitoneally, as follows:

For checkpoint blocking experiments: 200 μg anti‐CTLA4 (clone 9D9 BioXCell, #BE0164) or isotype control antibody (clone MCP‐11 BioXCell, #BE0086) were administered intraperitoneally at day 3 upon leukemia injection, followed by 100 μg every 3–4 days for a total of five infusions; 200 μg anti‐LAG 3 (clone C9B7W BioXCell, #BE0174) or isotype control antibody (clone HRPN BioXCell, #BE0088) were administered intraperitoneally at day 3 upon leukemia injection, followed by 100 μg every 3–4 days, for a total of five infusions; 8.3 mg 1‐Methyl‐dl‐tryptophan (1‐MT; 860646 Sigma) was administered intraperitoneally at day 3 upon leukemia injection, and every 3–4 days, for a total of five infusions.

For solid tumors challenge, mice were subcutaneously injected in the lower right flank with 5 × 10^5^ MC38 cells. Growth of tumor mass was followed every 3–4 days by caliper measurement. Tumor volumes were calculated by: *V* = 0.5236 × length × width^2^. Cells were tested for mycoplasma contamination.

### Blood counts and flow cytometry

Blood cell counts and hemoglobin levels were obtained on whole blood using ProCyte Dx Hematology Analyzer (IDEXX). All cytometric analyses were performed on FACSCanto II and LSRFortessa instruments (BD Bioscience) and analyzed with the FlowJo software (v. 10.5.3, Tree Star Inc.). Staining of the different compartments was performed as in Escobar *et al,* 2018 (Escobar *et al*, 2018). A list of antibodies and staining conditions is provided in Appendix Table S3. The gating strategy is shown in Appendix Fig S3. Solid tumors were dissociated in Collagenase 200 μg/ml (C5138, Sigma‐Aldrich), Dispase II 2 mg/ml (17105041, Thermo Fisher Scientific), and DNase I 100 U/ml (04716728001, Roche) for 45 min at 37°C and stained as previously described (Escobar *et al*, 2014).

### Single‐cell RNA sequencing

#### Cell preparation

Viable (Annexin V‐) BM cells were subjected to 2‐way sorting, separating B cells from non‐B cells: one way to collect CD19^+^ OFP^+/−^ cells and the other way to collect CD45^+^CD19^−^ cells. Granulocytes were excluded via Ly6g to reduce percentages to ≈ 15–30%. CTRL d12: mouse D1 36% OFP^+^ disease, after sorting 31% granulocytes; mouse D2 53% OFP^+^ disease, after sorting 33% granulocytes. IFN‐γ d12: (i) mouse C3 21% OFP^+^ disease, after sorting 36% granulocytes; (ii) mouse C5 2.7% OFP^+^ disease, no exclusion of granulocytes (8%). CTRL d17: mouse A3 45% OFP^+^ disease, after sorting 35% granulocytes; mouse A4 51% OFP^+^ disease, after sorting 19% granulocytes. IFN‐γ d17: (iii) mouse B5 59% OFP^+^ disease, after sorting 11% granulocytes; mouse C1 55% OFP^+^ disease, after sorting 18% granulocytes; (iv) mouse D3 49% OFP^+^ disease, after sorting 16% granulocytes; mouse D5 51% OFP^+^ disease, after sorting 9% granulocytes. After sorting, cells were washed, counted, loaded onto the Chromium Controller (10× Genomics) for single‐cell bead encapsulation, and processed for library preparation using the Chromium Single Cell 5′ Library & Gel Bead Kit, Chromium Single Cell V(D)J Enrichment and Mouse T Cell Kit, according to manufacturer's instructions.

#### De‐multiplexing, quality control, pre‐processing, and unsupervised clustering

scRNAseq libraries (GEX and VDJ) were demultiplexed and processed by the Cell Ranger Single‐Cell Software Suite (version 3.1.0, 10X Genomics) using the GRCm38 reference genome and gene annotations (v3.0.0) provided by the manufacturer. In order to quantify custom elements delivered with the lentiviral constructs present in the B‐ALL model and used in the gene therapy protocol, synthetic chromosomes and annotations for each constructs/element were appended and included in the reference files. Default parameters were used in all Cell Ranger steps including demultiplexing (mkfastq), gene expression quantification (quant), and VDJ pipeline. We sequenced six samples, obtaining for each of them both single‐cell gene expression data and TCR data. The CTRL d17 was discarded from further analysis due to poor quality. We obtained a number of cells per sample ranging from 1,830 to 23,861, for a total of ≈ 85,000 cells (Table EV1). The median UMI count per cell ranged from 3,974 to 5,753, whereas the average number of reads per cell were comprised between 20,300 and 160,000. Regarding the BCR‐TCR repertoire libraries, we obtained a number of cells ranging from 1,113 to 4,826 and from 273 to 848 for the TCR and BCR samples. VDJ sample libraries with a number of cells < 50 were discarded from the analysis. Full Cell Ranger metrics can be inspected in Table EV1. Feature barcode‐filtered matrices from Cell Ranger output were used as input for Seurat R package v3. Each sample was analyzed independently following the standard Seurat workflow. In particular, cells with a number of expressed genes (nFeature_RNA) < 200 or > 6,000 and cells with a percentage of transcripts mapping to mitochondrial genes > 15 were discarded. Counts were normalized and scaled by a factor of 10,000. In order to reduce the dimensionality of the dataset, we identified the most variable genes (top 20% of total number of expressed genes) using the default method implemented in the Seurat package. Data were then scaled and regressed out for unwanted source of variability, by passing to the var.to.regress parameter in the ScaleData function the following variables: nCount_RNA, percent.mt, and CC.Difference. The CC.Difference is defined as the difference between S.Score and G2M.Score module scores calculated with the CellCycleScoring function using S and G2M features from the Regev reference gene sets, with genes converted for mouse species. By using the most variable genes, we calculated 100 PCs and selected top 50 PCs to perform cell clustering and UMAP dimensionality reduction. Clustering (short nearest neighbor [SNN] with Louvain modularity algorithm) was performed with standard parameters and using two different resolutions: 0.6 and 1.2. Each sample was inspected for the presence of doublets, even if the number of genes expressed by the cells was capped at 6,000 genes. We expected to have a percent of doublets ranging from 15% to 20% according to the number of cells detected. Considering the high number of cells recovered, we performed doublets removal by doubletfinder (v.2.0.3) (McGinnis *et al*, 2019) and doubletdecon (v1.1.4) (DePasquale *et al*, 2019) algorithms. Doublet finder was run by setting a number of PCs equal to 50, an expected doublet rate of 15% and a pK value equal to the value corresponding to the max BC metric obtained from the find.pK function. The clustering resolution used was 1.2 except for samples GEX2 and GEXN2, in which we used the 0.6 resolution. DoubletDecon was run using the Main_Doublet_Decon function with standard parameters. Metadata including doublets classification of both doubletdecon and doubletfinder were added to the Seurat objects. We applied both a conservative classification and an aggressive classification using intersection and union of doublets classification provided by the two software packages, respectively. For the analysis of the final objects, we applied the more stringent approach considering doublets the cells classified as such by at least one of the software packages.

#### Cell type annotation

Cell type annotation was assessed by using SingleR (v1.1.11), a computational method, based on correlation analysis that leverages reference transcriptomic datasets of pure cell types to infer the cell of origin of each of the single cells independently. We performed annotation by using the Mousernaseq and the Immgen reference dataset (Heng *et al*, 2008; Benayoun *et al*, 2019). We performed a double annotation using for each dataset both main labels (including cell types labels) and fine labels (including cell subtypes labels), the latter characterized by a more detailed classification of the former. Pruned labels associated with all main annotations were then imported in Seurat object. The disease annotation was carried on by highlighting cells expressing at least 1 UMI mapping to the TTA custom gene. In order to get more insights and details regarding the different cell type components, we split the full dataset into four main compartments: myeloid cells (monocytes + macrophages), lymphoid cells (NK‐T cells), B cells (including B‐ALL) and neutrophils. To perform this step, we set up a data analysis workflow that used both annotations (Mousernaseq main and Immgen main) by intersecting specific labels in order to get rid of discordant or poor‐quality annotations. In particular, we selected cells by intersecting selected groups of cells (Table EV2). After preliminary analyses on each of the subsets, outlier cell clusters spotted by marker inspection, which did not fit their compartment, were discarded from subsequent analyses. After common and standard processing with Seurat, as already described above, we applied data integration by using harmony (v.1.0, Korsunsky *et al*, 2019), including the orig.ident variable in the group.by.vars parameter. Cluster marker identification was performed at 0.6 and 1.2 resolutions (FindClusters parameter) with the FindAllMarkers function (min.pct = 0.25, min.cells.group = 10), focusing on upregulated genes only. The treatment effect within each cluster identified was assessed with the FindMarkers function, setting parameters according to the comparison to be perform. In particular, we consider as untreated cells only the ones belonging to the sample GEXN1, due to the low number of cells present in the GEXN2 sample. Gene ontology overrepresentation analysis (ORA) was performed with the ClusterProfiler R package (enrichGO function with default parameters) using as input the marker lists collected and as gene universe the list of genes expressed in the dataset under analysis. Gene Set Enrichments Analysis was performed by using the GSEA function in clusterprofiler R package. In particular, we applied this data analysis approach for interpreting results obtained from intra‐clusters comparisons and in group vs. group comparisons. The inputs to GSEA were logFC‐ranked list of genes obtained from findallmarker function setting logFC to 0 and return.thresh parameter to 1. The study of specific expression programs was assessed by the evaluation of custom and literature‐based gene signatures by using the AddModuleScore function from the Seurat package. Myeloid‐specific IFNγ‐related gene signature: *Stat1*, *Irf1*, *Ido1*, *Ifng*, *Il12a*, *Igtp*, *Ifit1*, *Ifit3*, *H2*‐*Ab1*, *H2*‐*Oa*, *H2*‐*Eb1*, *H2*‐*DMb2*, *H2*‐*Aa*, *H2*‐*DMb1*, *H2*‐*Dma*, *Ciita*; lymphoid‐specific and B‐cell‐specific IFNγ‐related gene signature: *Irf1*, *Ido1*, *Ifng*, *Il12a*, *Igtp*, *Ifit1*, *Ifit3*. MHC II signature: *H2*‐*Ab1*, *H2*‐*Oa*, *H2*‐*Eb1*, *H2*‐*DMb2*, *H2*‐*Aa*, *H2*‐*DMb1*, *H2*‐*DMa*, *Ciita*.

#### TCR repertoire analysis

The evaluation of the TCR repertoire was assessed both by inspecting the results obtained from the Cell Ranger VDJ pipeline reports and by using the scRepertoire R package (v0.99.1), https://github.com/ncborcherding/scRepertoire). In particular, after the analysis of both absolute and relative clonotype abundance across samples and conditions (treated vs. untreated groups) with scRepertoire, we focused on the mapping of highly abundant clonotypes (in particular, the top10 most abundant for each condition) onto the UMAP embedding of single‐cell expression datasets in order to identify the presence of cluster‐specific TCR.

### Statistical analysis

Values for statistical significance have been calculated using GraphPad Prism. The respective method used is given in the figure legends. Figures show meanhow mean  ± standard deviation, unless otherwise stated.

## Author contributions

AM and GA planned and performed experiments, analyzed, and interpreted the data, and wrote the manuscript; CC planned and performed experiments, analyzed, and interpreted the data shown in Figs 1H and I, and EV4. FMV performed experiments and analyzed the data. GD performed and analyzed experiments and provided technical support. RP performed experiments and analyzed data. BGr and MC provided CAR‐T cells and intellectual input. GE contributed to the experimental setup and provided intellectual input; LP helped validate the human IFN‐γ construct; NL provided IFN‐γR1 knockout mice and critically reviewed the manuscript; FS performed histopathologic analysis and wrote the toxicity report. IM and MB developed and executed the bioinformatics analysis; LN provided intellectual input and critically reviewed the manuscript; BGe provided financial support, supervised the research, interpreted the data, and wrote the manuscript.

## Conflict of interest

B.Ge. is a founder of Genenta Science, a biotech start‐up financing the clinical development of IFN‐α gene therapy. Genenta did not fund the research on IFN‐γ gene therapy presented in this manuscript, nor does Genenta have ownership rights on the data presented herein.

## Supporting information



Appendix

Expanded View Figures PDF

Table EV1

Table EV2

Table EV3

Table EV4

Table EV5

## Data Availability

The datasets and computer code produced in this study are available in the following database: RNA‐Seq data: Gene Expression—accession number GSE178941 (http://www.ncbi.nlm.nih.gov/geo/query/acc.cgi?acc=GSE178941).
