# Supplementary material for: Myeloid cell‐based delivery of IFN‐γ reprograms the leukemia microenvironment and induces anti‐tumoral immune responses
Source: EMBO Mol Med. 2021 Aug 30;13(10):e13598. doi: 10.15252/emmm.202013598 (PMC8495462; doi:10.15252/emmm.202013598)
Supplement: Supplementary file 1 — Appendix [file EMMM-13-e13598-s002.pdf]

Table of content

Appendix Figure S1. IFNγ efficacy in a solid tumor model of colorectal carcinoma.

Appendix Figure S2. IFNγ signature in the myeloid and lymphoid compartments.

Appendix Figure S3. Gating strategies for myeloid and lymphoid subpopulations.

Appendix Table S1. *In vivo* transplantation of murine lineage negative cells expressing gene of interest under a strong myeloid specific promoter (SP146/gp91).

Appendix Table S2. Primers for ddPCR assays.

Appendix Table S3. Antibodies utilized for flow cytometry.

Appendix Table S4. Statistical analysis of Figure 1 panel A.

Appendix Table S5. Statistical analysis of Figure 1 panel I.

Appendix Table S6. Statistical analysis of Figure 2 panel C.

Appendix Table S7. Statistical analysis of Figure 6 panel C.

Appendix Table S8. Statistical analysis of Figure 6 panel E.

Pathology report from IFNγ-engrafted mice.

Appendix Figure S1

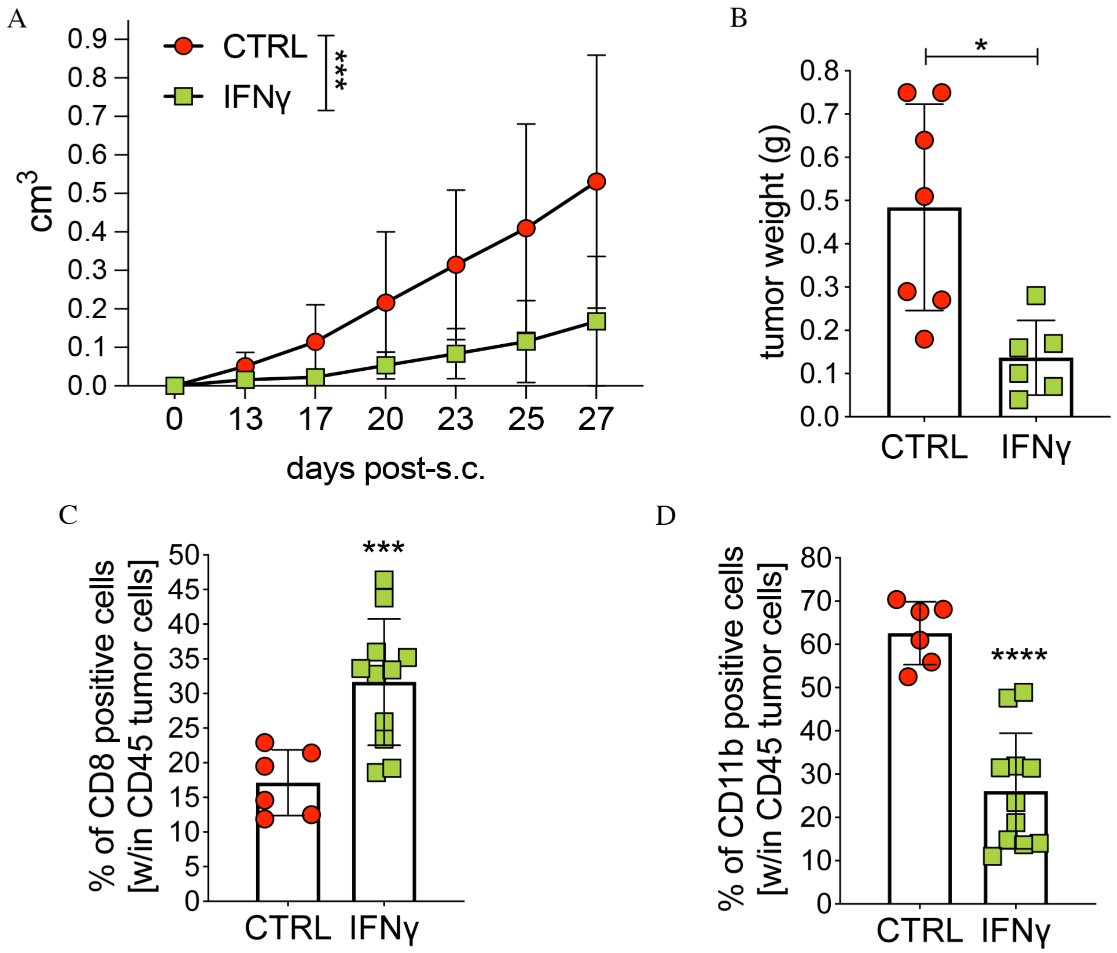

Appendix Figure S1. IFNγ efficacy in a solid tumor model of colorectal carcinoma.

(**A**) Tumor volume over-time (one experiment, CTRL = 7 mice, IFNγ = 6 mice, mean ± SD; ***$p \leq 0.001$, Two-Way ANOVA).

(**B**) Tumor weight at endpoint analysis after resection (mean ± SD, each dot represents an individual mouse; *$p \leq 0.05$, ordinary One-Way ANOVA).

(**C**) Percentage of CD8+ T lymphocytes within CD45+ hematopoietic cells infiltrating the tumor (mean ± SD, each dot represents an individual mouse, 2 independent experiments, ***$p \leq 0.001$, ordinary One-Way ANOVA).

(**D**) Percentage of CD11b+ myeloid cells within CD45 hematopoietic cells infiltrating the tumor (mean ± SD, each dot represents an individual mouse, 2 independent experiments, ****$p \leq 0.0001$, ordinary One-Way ANOVA).

Appendix Figure S2

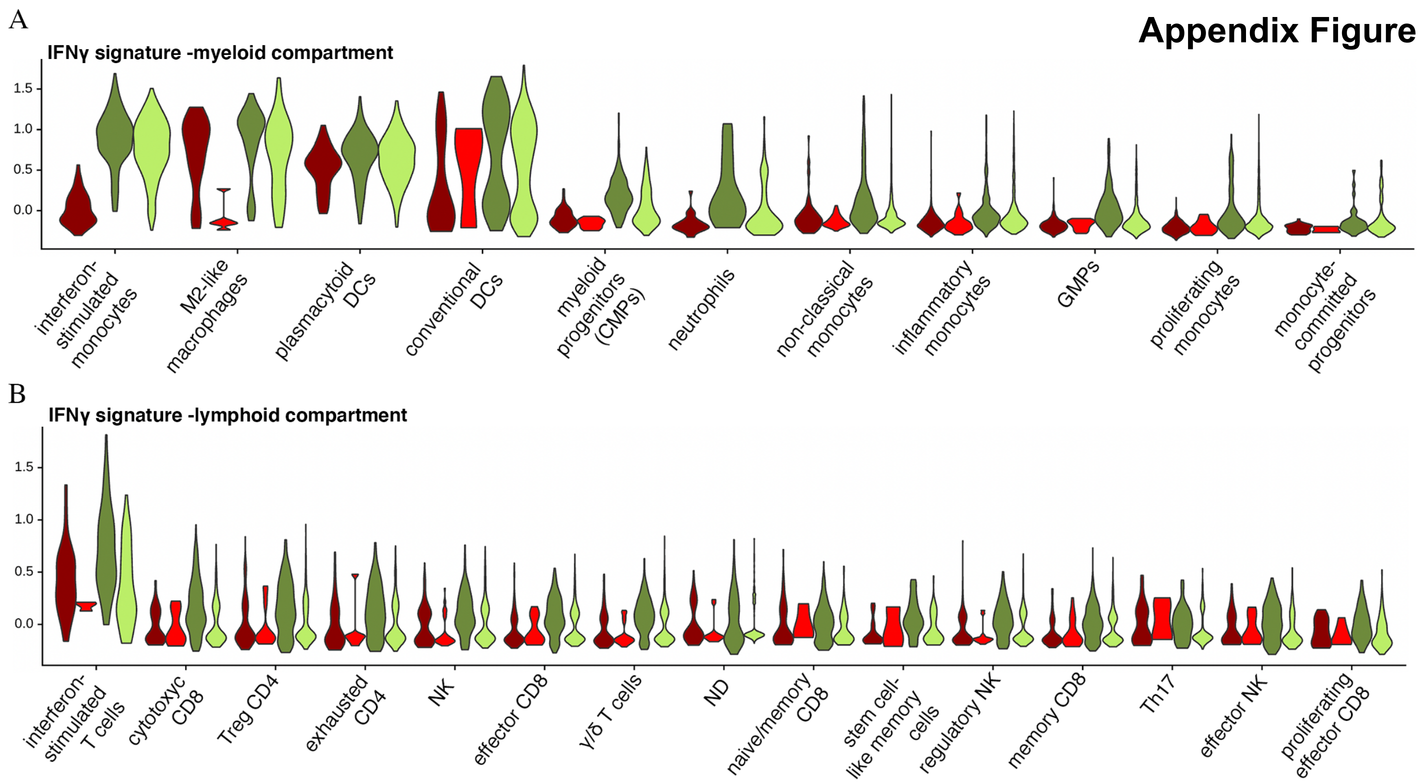

Appendix Figure S2. IFNγ signature in the myeloid and lymphoid compartments.

(**A**) Violin plots showing myeloid-specific IFNγ gene signature expression (*Stat1*, *Irf1*, *Ido1*, *Ifng*, *Il12a*, *Igtp*, *Ifit1*, *Ifit3*, *H2-Ab1*, *H2-Oa*, *H2-Eb1*, *H2-DMb2*, *H2-Aa*, *H2-DMb1*, *H2-Dma*, *Ciita*) within myeloid cell clusters, derived from the scRNAseq data. Statistics by Wilcox Rank Sum Test, IFNγ d12 vs. CTRL d12: conventional DCs, p = 0.0005; plasmacytoid DCs, p= 0.002; T cells p=0.1; interferon stimulated monocytes, p= $9x10^{-50}$; myeloid progenitors (CMPs), p= $8x10^{-14}$; proliferating monocytes, p= $8x10^{-57}$; inflammatory monocytes, p= $1.6x10^{-30}$; GMPs, p= $1.2x10^{-10}$; M2-like macrophages, p= 0.2; non-classical monocytes, p= $6.2x10^{-5}$; neutrophils, p= 0.0006; monocyte committed progenitors, p= 0.0004).

(**B**) Violin Plots of a lymphoid-specific IFNγ-related gene signature (*Irf1*, *Ido1*, *Ifng*, *Il12a*, *Igtp*, *Ifit1*, *Ifit3*) on lymphoid clusters, derived from the scRNAseq data. Statistics by Wilcox Rank Sum Test, IFNγ d12 vs. CTRL d12: γ/δ T cells, p= $4.3x10^{-11}$; cytotoxic CD8, p= 0.0004; ND, p= 0.17; effector NK, p= 0.69; regulatory NK, p= $1.6x10^{-6}$; proliferating effector CD8, p= 0.6; NK, p= $1,6x10^{-5}$; IFN-stimulated T cells, p=0.4; effector CD8, p= $6.9x10^{-11}$; stem cell-like memory cells, p=0.002; memory CD8, p= $2.7x10^{-8}$; Treg CD4, p= 0.002; Th17, p= 0.6; exhausted CD4, p= 0.008; naïve/memory CD8, p= 0.6).

Appendix Figure S3

A

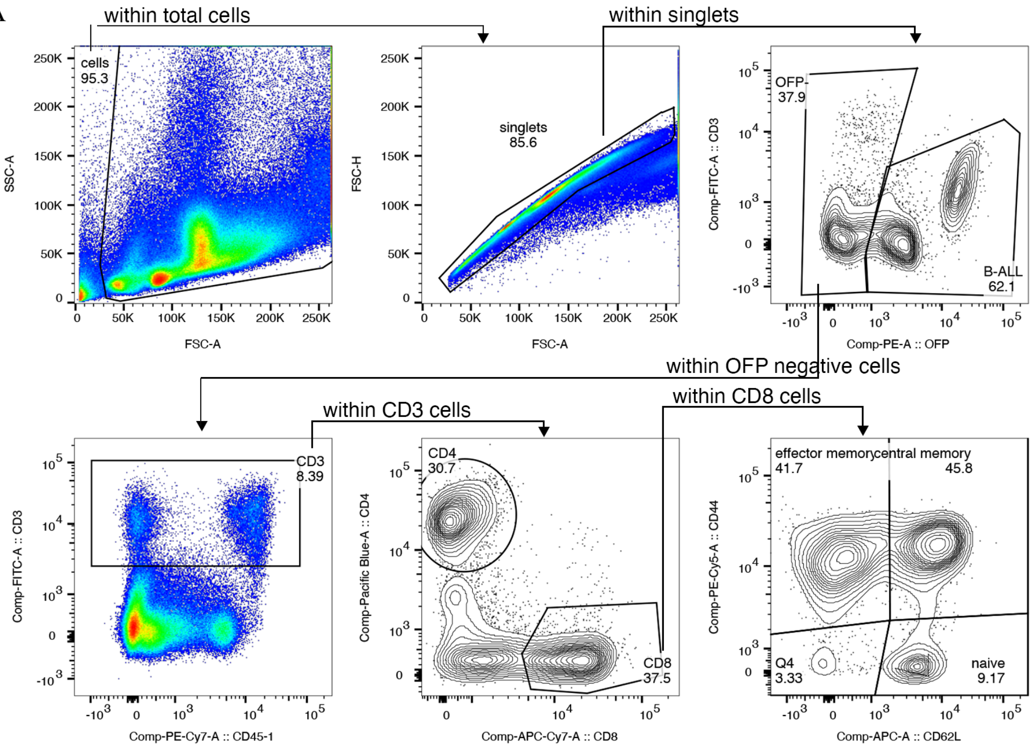

B

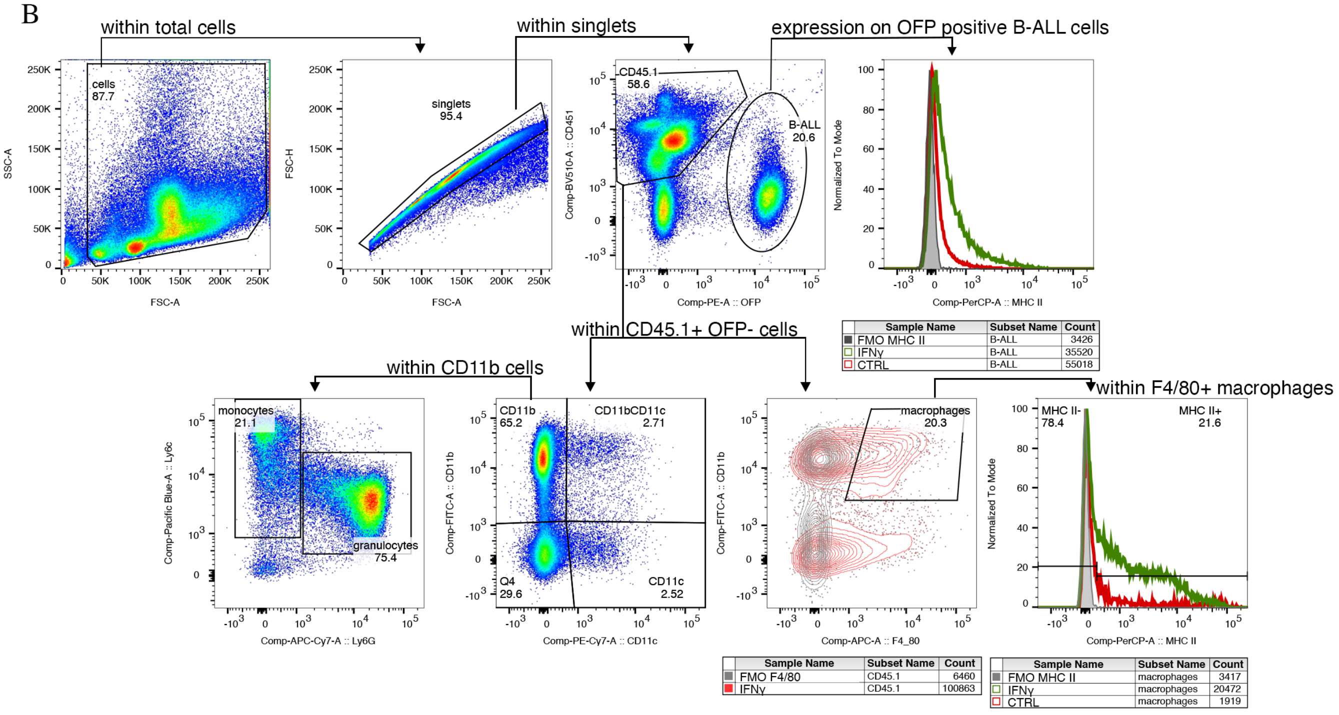

Appendix Figure S3. Gating strategies for myeloid and lymphoid subpopulations.

(A) Representative FACS plots showing gating strategy for lymphoid subpopulations

(B) Representative FACS plots showing gating strategy for myeloid subpopulations

Appendix Table S1. In vivo transplantation of murine lineage negative cells expressing gene of interest under a strong myeloid specific promoter (SP146/gp91)

**SP146/gp91phox.NGFR.miRT**	**SP146/gp91phox.IFNγ.miRT**
8 mice	8 mice
MOI 50	MOI 50
cell dose: 500'000/mouse	cell dose: 500'000/mouse
VCN *in vitro* : 3.8	VCN *in vitro* : 4.05
VCN *in vivo* (BM): 5.32	VCN *in vivo (BM)* : 3.45

Appendix Table S2. Primers for ddPCR assays.

TARGET		SEQUENCE
**HIV**	**Forward**	5′-TACTGACGCTCTCGCACC-3′
	**Probe**	FAM 5′-ATCTCTCTCCTTCTAGCCTC-3′
	**Reverse**	5′-TCTCGACGCAGGACTCG-3′
**Tie2**	**Forward**	5’- CTGTGGTGTTTCTCCTTG-3’
**IFNα**	**Probe**	FAM 5’-TGATGGTCCTGGCGGTGCTGAGCT-3’ BHQ2
	**Reverse**	5’-GAGGTTATGAGTCTGAGG-3’
**IFNγ**	**Probe**	FAM 5’-TGGCTGTTACTGCCACGGCACAGT-3’ BHQ2
	**Reverse**	5’-CTTCCACATCTATGCCAC-3’
**TNFα**	**Probe**	FAM 5’-CACCATGAGCACAGAAAGCATGATCCGCGA-3’ BHQ2
	**Reverse**	5’-GAAGAGGCTGAGACATAG-3’
**Sema3a**	**Forward**	5′-ACCGATTCCAGATGATTGGC-3′
	**Probe**	HEX 5′-AGAGGCCTGTCCTGCAGCTCATGG-3′ BHQ1
	**Reverse**	5′-TCCATATTAATGCAGTGCTTGC-3′

Appendix Table S3. Antibodies utilized for flow cytometry.

antigen	name	color	clone	catalog #	company	dilution
**CD16/CD32**	Purified Rat Anti-Mouse CD16/CD32 (Mouse BD Fc Block)	purified	2.4G2	553142	BD Biosciences	1 : 100
**CD45**	Anti-Mo CD45, eBioscience	APC	30-F11	17-0451-82	Thermo Fisher Scientific	1 : 100
**CD45.1**	eBioscience Anti-Mouse CD45.1	APC	A20	17-0453-82	Thermo Fisher Scientific	1 : 100
**CD45.1**	eBioscience Anti-Mouse CD45.1	FITC	A20	11-0453-82	Thermo Fisher Scientific	1 : 100
**CD45.1**	Brilliant Violet 510 anti-mouse CD45.1	BV510	A20	110741	BioLegend	1 : 100
**CD45.1**	PE Mouse Anti-Mouse Cd45.1	PE	A20	553776	BD Biosciences	1 : 100
**CD45.1**	PE/Cy7 anti-mouse CD45.1	PE/Cy7	A20	110730	BioLegend	1 : 100
**CD45.2**	Pacific Blue anti-Mouse CD45.2	Pacific Blue	104	109820	BioLegend	1 : 100
**CD45.2**	eBioscience Anti-Mouse CD45.2	PE	104	12-0454-82	Thermo Fisher Scientific	1 : 100
**CD45.2**	Brilliant Violet 510 anti-mouse CD45.2	BV510	104	109838	BioLegend	1 : 100
**CD45.2**	eBioscience Anti-Mouse CD45.2	FITC	104	11-0454-82	Thermo Fisher Scientific	1 : 100
**CD19**	Anti-Mouse CD19	APC	eBio1D3	17-0193-82	Thermo Fisher Scientific	1 : 100
**CD19**	Brilliant Violet 510 anti-mouse CD19	BV510	6D5	115545	BioLegend	1 : 100
**CD19**	PE/Cy7 anti-mouse CD19	PE/Cy7	6D5	115520	BioLegend	1 : 100
**CD19**	PE Rat Anti-Mouse CD19	PE	1D3	553786	BD Biosciences	1 : 100
**CD3e**	eBioscience Anti-Mouse CD3e	PE	145-2C11	12-0031-81	Thermo Fisher Scientific	1 : 100
**CD3e**	eBioscience Anti-Mouse CD3e	FITC	145-2C11	11-0031-82	Thermo Fisher Scientific	1 : 100
**CD4**	Anti-Mouse CD4 eBioscience	eFluo450	RM4-5	48-0042-82	Thermo Fisher Scientific	1 : 200
**CD4**	Anti-Mouse CD4 eBioscience	AlexaFluor700	GK1.5	56-0041-80	Thermo Fisher Scientific	1 : 100
**CD4**	Anti-Mouse CD4 eBioscience	APC	GK1.5	17-0041-82	Thermo Fisher Scientific	1 : 100
**CD8a**	CD8a/Ly-2 Rt Anti-Ms mAb	APC/Cy7	53-6.7	A15386	Life Technologies	1 : 100
**CD8a**	Brilliant Violet 510 Anti-Mouse CD8a	BV510	53-6.7	100751	BioLegend	1 : 200
**CD11b**	FITC Rat Anti-Mouse CD11b	FITC	M1/70	553310	BD Biosciences	1 : 100
**CD11b**	PE-Cy7 Rat Anti-CD11b	PE/Cy7	M1/70	561098	BD Biosciences	1 : 100
**CD11b**	APC/Cyanine7 anti-mouse/human CD11b	APC/Cy7	M1/70	101226	BioLegend	1 : 100
**CD11b**	PE Rat Anti-Mouse CD11b	PE	M1/70	553311	BD Biosciences	1 : 100
**CD11c**	eBioscience Anti-Mouse CD11c	eFluor450	N418	48-0114-82	Thermo Fisher Scientific	1 : 100
**CD11c**	FITC Hamster Anti-Mouse CD11c	FITC	HL3	557400	BD Biosciences	1 : 100
**CD150**	Brilliant Violet 510™ anti-mouse CD150 (SLAM) Antibody	BV510	TC15-12F12.2	115929	BioLegend	1 : 100
**CD34**	PE anti-mouse CD34 Antibody	PE	MEC14.7	119307	BioLegend	1 : 100
**CD44**	PE-Cy5 Rat Anti-Mouse CD44	PE/Cy5	IM7	561861	BD Biosciences	1 : 200
**CD62L**	APC Anti-Mouse CD62L	APC	MEL-14	104412	BioLegend	1 : 100
**CD103**	BV711 Rat Anti-Mouse CD103	BV711	M290	564320	BD Biosciences	1 : 100
**MHC II**	Brilliant Violet 510 anti-mouse I-A/I-E	BV510	M5/114.15.2	107635	BioLegend	1 : 100
**MHC II**	PerCP-Cy5.5 Rat Anti-Mouse I-A/I-E	PerCP/Cy5.5	M5/114.15.2	562363	BD Biosciences	1 : 100
**PD-1**	PE-CF594 Hamster Anti-Mouse CD279	PE-CF594	J43	562523	BD Biosciences	1 : 100
**LAG-3**	PerCP-Cy5.5 Rat Anti-Mouse CD223	PerCP/Cy5.5	C9B7W	564673	BD Biosciences	1 : 100
**CD25**	eBioscience Anti-Mouse CD25	PE/Cy7	PC61.5	25-0251-82	Thermo Fisher Scientific	1 : 100
**Ly6G**	APC-Cy7 Rat Anti-Mouse Ly-6G	APC/Cy7	1A8	560600	BD Biosciences	1 : 200
**Ly6C**	Anti-Mo Ly-6C eBioscience	eFluo450	HK1.4	48-5932-82	Thermo Fisher Scientific	1 : 200
**LNGFR (CD271)**	PE-Vio770, Human	PE-Vio770	ME20.4-1-H4	130-113-422	Miltenyi Biotec	1 : 100
**LNGFR (CD271)**	BV510 Mouse Anti-Human CD271	BV510	C40-1457	563451	BD Biosciences	1 : 100
**Lin+ cocktail**	Anti-Mouse Hematopoietic Lineage Antibody Cocktail, eBioscience	eFluor450		88-7772-72	Thermo Fisher Scientific	1 : 100
**c-Kit**	APC Rat Anti-Mouse CD117	APC	2B8	553356	BD Biosciences	1 : 100
**Sca-1**	PE-Cy7 Rat Anti-Mouse Ly-6A/E	PE/Cy7	D7	558162	BD Biosciences	1 : 100
**F4/80**	Rat Anti-Mouse F4/80: RPE	RPE	CI:A3-1	MCA497PE	BioRad	1 : 100
**F4/80**	Rat Anti-Mouse F4/80: APC	APC	CI:A3-1	MCA497APC	BioRad	1 : 100
**CD19**	FITC Mouse Anti-Human CD19	FITC	HIB19 (RUO)	555412	BD Biosciences	2 : 100
**CD33**	CD33-VioBlue, human for 100 tests	VioBlue	REA775	130-111-024	Miltenyi Biotec	2 : 100

Appendix Table S4

Figure 1A

Compare each cell mean with the other cell mean in that row								
**Number of families**	1							
**Number of comparisons per family**	4							
**Alpha**	0.05							
**Šídák's multiple comparisons test**	Predicted (LS) mean diff.	95.00% CI of diff.	Below threshold?	Summary	Adjusted P Value			
**CTRL - IFNγ**								
**10**	81.93	-15280 to 15444	No	ns	>0.9999			
**12**	3815	-11547 to 19177	No	ns	0.9479			
**14**	10132	-9071 to 29334	No	ns	0.5425			
**17**	71777	52574 to 90979	Yes	****	<0.0001			
**Test details**	Predicted (LS) mean 1	Predicted (LS) mean 2	Predicted (LS) mean diff.	SE of diff.	N1	N2	t	DF
**CTRL - IFNγ**								
**10**	96.37	14.44	81.93	5915	6	10	0.01385	44.00
**12**	4109	293.7	3815	5915	6	10	0.6449	44.00
**14**	16187	6056	10132	7394	4	6	1.370	44.00
**17**	108472	36695	71777	7394	4	6	9.707	44.00

Appendix Table S5

Figure 1I

**Compare each cell mean with the other cell mean in that row.**								
**Number of families**	1							
**Number of comparisons per family**	3							
**Alpha**	0,05							
**Sidak's multiple comparisons test**	Mean Diff.	95% CI of diff.	Significant?	Summary	Adjusted P Value			
**CTRL - IFNγ**								
**14**	1101	-1.577e+007 to 1.577e+007	No	ns	> 0.9999			
**16**	479277	-1.529e+007 to 1.625e+007	No	ns	0,9998			
**19**	4.264E+10	2.687e+007 to 5.841e+007	Yes	****	< 0.0001			
**Test details**	Mean 1	Mean 2	Mean Diff.	SE of diff.	N1	N2	t	DF
**CTRL - IFNγ**								
**14**	16124	15023	1101	6.389E+09	9	10	0,0001723	51
**16**	1.053E+09	573336	479277	6.389E+09	9	10	0,07502	51
**19**	8.404E+10	4.14E+10	4.264E+10	6.389E+09	9	10	6,674	51

Appendix Table S6

Figure 2C

Within each row, compare columns (simple effects within rows)								
**Number of families**	3							
**Number of comparisons per family**	6							
**Alpha**	0.05							
**Tukey's multiple comparisons test**	Mean Diff.	95.00% CI of diff.	Below threshold?	Summary	Adjusted P Value			
**7**								
**WT CTRL vs. KO CTRL**	-14.97	-6918 to 6888	No	ns	>0.9999			
**WT CTRL vs. WT IFNγ**	7.008	-6896 to 6910	No	ns	>0.9999			
**WT CTRL vs. KO IFNγ**	-13.72	-6917 to 6890	No	ns	>0.9999			
**KO CTRL vs. WT IFNγ**	21.98	-6881 to 6925	No	ns	>0.9999			
**KO CTRL vs. KO IFNγ**	1.254	-6902 to 6905	No	ns	>0.9999			
**WT IFNγ vs. KO IFNγ**	-20.73	-6924 to 6883	No	ns	>0.9999			
**10**								
**WT CTRL vs. KO CTRL**	-2404	-9307 to 4500	No	ns	0.7965			
**WT CTRL vs. WT IFNγ**	3457	-3446 to 10360	No	ns	0.5552			
**WT CTRL vs. KO IFNγ**	-4020	-10923 to 2884	No	ns	0.4243			
**KO CTRL vs. WT IFNγ**	5861	-1043 to 12764	No	ns	0.1242			
**KO CTRL vs. KO IFNγ**	-1616	-8520 to 5287	No	ns	0.9268			
**WT IFNγ vs. KO IFNγ**	-7477	-14380 to -573.3	Yes	*	0.0286			
**12**								
**WT CTRL vs. KO CTRL**	-11786	-18690 to -4883	Yes	***	0.0002			
**WT CTRL vs. WT IFNγ**	7957	1054 to 14860	Yes	*	0.0174			
**WT CTRL vs. KO IFNγ**	-10046	-16950 to -3143	Yes	**	0.0015			
**KO CTRL vs. WT IFNγ**	19743	12840 to 26647	Yes	****	<0.0001			
**KO CTRL vs. KO IFNγ**	1740	-5164 to 8643	No	ns	0.9107			
**WT IFNγ vs. KO IFNγ**	-18003	-24907 to -11100	Yes	****	<0.0001			
**Test details**	Mean 1	Mean 2	Mean Diff.	SE of diff.	N1	N2	q	DF
**7**								
**WT CTRL vs. KO CTRL**	8.506	23.48	-14.97	2625	7	7	0.008066	72.00
**WT CTRL vs. WT IFNγ**	8.506	1.497	7.008	2625	7	7	0.003776	72.00
**WT CTRL vs. KO IFNγ**	8.506	22.22	-13.72	2625	7	7	0.007391	72.00
**KO CTRL vs. WT IFNγ**	23.48	1.497	21.98	2625	7	7	0.01184	72.00
**KO CTRL vs. KO IFNγ**	23.48	22.22	1.254	2625	7	7	0.0006754	72.00
**WT IFNγ vs. KO IFNγ**	1.497	22.22	-20.73	2625	7	7	0.01117	72.00
**10**								
**WT CTRL vs. KO CTRL**	3727	6130	-2404	2625	7	7	1.295	72.00
**WT CTRL vs. WT IFNγ**	3727	269.5	3457	2625	7	7	1.863	72.00
**WT CTRL vs. KO IFNγ**	3727	7746	-4020	2625	7	7	2.166	72.00
**KO CTRL vs. WT IFNγ**	6130	269.5	5861	2625	7	7	3.158	72.00
**KO CTRL vs. KO IFNγ**	6130	7746	-1616	2625	7	7	0.8707	72.00
**WT IFNγ vs. KO IFNγ**	269.5	7746	-7477	2625	7	7	4.028	72.00
**12**								
**WT CTRL vs. KO CTRL**	11689	23475	-11786	2625	7	7	6.350	72.00
**WT CTRL vs. WT IFNγ**	11689	3732	7957	2625	7	7	4.287	72.00
**WT CTRL vs. KO IFNγ**	11689	21736	-10046	2625	7	7	5.413	72.00
**KO CTRL vs. WT IFNγ**	23475	3732	19743	2625	7	7	10.64	72.00
**KO CTRL vs. KO IFNγ**	23475	21736	1740	2625	7	7	0.9373	72.00
**WT IFNγ vs. KO IFNγ**	3732	21736	-18003	2625	7	7	9.700	72.00

Appendix Table S7

Figure 6C

Within each row, compare columns (simple effects within rows)								
**Number of families**	2							
**Number of comparisons per family**	10							
**Alpha**	0.05							
**Šídák's multiple comparisons test**	Predicted (LS) mean diff.	95.00% CI of diff.	Below threshold?	Summary	Adjusted P Value			
**13**								
**CTRL vs. IFNα**	3613	-17441 to 24668	No	ns	>0.9999			
**CTRL vs. IFNα/IFNγ**	1807	-14274 to 17887	No	ns	>0.9999			
**CTRL vs. IFNα/TNFα**	1556	-16336 to 19449	No	ns	>0.9999			
**CTRL vs. IFNγ/TNFα**	3850	-6870 to 14570	No	ns	0.9721			
**IFNα vs. IFNα/IFNγ**	-1807	-25346 to 21732	No	ns	>0.9999			
**IFNα vs. IFNα/TNFα**	-2057	-26870 to 22755	No	ns	>0.9999			
**IFNα vs. IFNγ/TNFα**	236.6	-20023 to 20496	No	ns	>0.9999			
**IFNα/IFNγ vs. IFNα/TNFα**	-250.2	-21010 to 20509	No	ns	>0.9999			
**IFNα/IFNγ vs. IFNγ/TNFα**	2043	-12981 to 17068	No	ns	>0.9999			
**IFNα/TNFα vs. IFNγ/TNFα**	2294	-14656 to 19244	No	ns	>0.9999			
**15**								
**CTRL vs. IFNα**	36202	14009 to 58394	Yes	***	0.0001			
**CTRL vs. IFNα/IFNγ**	27490	9945 to 45034	Yes	***	0.0003			
**CTRL vs. IFNα/TNFα**	21612	2393 to 40831	Yes	*	0.0178			
**CTRL vs. IFNγ/TNFα**	34791	21979 to 47603	Yes	****	<0.0001			
**IFNα vs. IFNα/IFNγ**	-8712	-32251 to 14827	No	ns	0.9658			
**IFNα vs. IFNα/TNFα**	-14590	-39402 to 10223	No	ns	0.6217			
**IFNα vs. IFNγ/TNFα**	-1411	-21670 to 18849	No	ns	>0.9999			
**IFNα/IFNγ vs. IFNα/TNFα**	-5878	-26637 to 14882	No	ns	0.9952			
**IFNα/IFNγ vs. IFNγ/TNFα**	7301	-7723 to 22326	No	ns	0.8313			
**IFNα/TNFα vs. IFNγ/TNFα**	13179	-3771 to 30129	No	ns	0.2432			
**Test details**	Predicted (LS) mean 1	Predicted (LS) mean 2	Predicted (LS) mean diff.	SE of diff.	N1	N2	t	DF
**13**								
**CTRL vs. IFNα**	4610	996.5	3613	7244	10	2	0.4988	60.00
**CTRL vs. IFNα/IFNγ**	4610	2803	1807	5533	10	4	0.3265	60.00
**CTRL vs. IFNα/TNFα**	4610	3054	1556	6156	10	3	0.2528	60.00
**CTRL vs. IFNγ/TNFα**	4610	759.9	3850	3689	10	18	1.044	60.00
**IFNα vs. IFNα/IFNγ**	996.5	2803	-1807	8099	2	4	0.2231	60.00
**IFNα vs. IFNα/TNFα**	996.5	3054	-2057	8537	2	3	0.2410	60.00
**IFNα vs. IFNγ/TNFα**	996.5	759.9	236.6	6971	2	18	0.03394	60.00
**IFNα/IFNγ vs. IFNα/TNFα**	2803	3054	-250.2	7143	4	3	0.03503	60.00
**IFNα/IFNγ vs. IFNγ/TNFα**	2803	759.9	2043	5170	4	18	0.3953	60.00
**IFNα/TNFα vs. IFNγ/TNFα**	3054	759.9	2294	5832	3	18	0.3933	60.00
**15**								
**CTRL vs. IFNα**	52701	16500	36202	7636	6	2	4.741	60.00
**CTRL vs. IFNα/IFNγ**	52701	25212	27490	6037	6	4	4.554	60.00
**CTRL vs. IFNα/TNFα**	52701	31089	21612	6613	6	3	3.268	60.00
**CTRL vs. IFNγ/TNFα**	52701	17910	34791	4408	6	18	7.892	60.00
**IFNα vs. IFNα/IFNγ**	16500	25212	-8712	8099	2	4	1.076	60.00
**IFNα vs. IFNα/TNFα**	16500	31089	-14590	8537	2	3	1.709	60.00
**IFNα vs. IFNγ/TNFα**	16500	17910	-1411	6971	2	18	0.2024	60.00
**IFNα/IFNγ vs. IFNα/TNFα**	25212	31089	-5878	7143	4	3	0.8229	60.00
**IFNα/IFNγ vs. IFNγ/TNFα**	25212	17910	7301	5170	4	18	1.412	60.00
**IFNα/TNFα vs. IFNγ/TNFα**	31089	17910	13179	5832	3	18	2.260	60.00

Appendix Table S8

Figure 6E

Within each row, compare columns (simple effects within rows)					
**Number of families**	3				
**Number of comparisons per family**	28				
**Alpha**	0.05				
**Tukey's multiple comparisons test**	Predicted (LS) mean diff.	95.00% CI of diff.	Below threshold?	Summary	Adjusted P Value
**11**					
**CTRL isotype vs. CTRL +αCTLA4**	1370	-82114 to 84854	No	ns	>0.9999
**CTRL isotype vs. CTRL +αLAG3**	1932	-87062 to 90927	No	ns	>0.9999
**CTRL isotype vs. CTRL +1-MT**	-1609	-90604 to 87385	No	ns	>0.9999
**CTRL isotype vs. IFNγ isotype**	1843	-72616 to 76301	No	ns	>0.9999
**CTRL isotype vs. IFNγ +αCTLA4**	2167	-74537 to 78871	No	ns	>0.9999
**CTRL isotype vs. IFNγ +αLAG3**	2507	-74196 to 79211	No	ns	>0.9999
**CTRL isotype vs. IFNγ +1-MT**	2409	-77190 to 82009	No	ns	>0.9999
**CTRL +αCTLA4 vs. CTRL +αLAG3**	562.1	-91924 to 93048	No	ns	>0.9999
**CTRL +αCTLA4 vs. CTRL +1-MT**	-2979	-95465 to 89507	No	ns	>0.9999
**CTRL +αCTLA4 vs. IFNγ isotype**	472.4	-78125 to 79070	No	ns	>0.9999
**CTRL +αCTLA4 vs. IFNγ +αCTLA4**	796.9	-79931 to 81525	No	ns	>0.9999
**CTRL +αCTLA4 vs. IFNγ +αLAG3**	1137	-79591 to 81865	No	ns	>0.9999
**CTRL +αCTLA4 vs. IFNγ +1-MT**	1039	-82445 to 84524	No	ns	>0.9999
**CTRL +αLAG3 vs. CTRL +1-MT**	-3541	-101030 to 93947	No	ns	>0.9999
**CTRL +αLAG3 vs. IFNγ isotype**	-89.68	-84517 to 84338	No	ns	>0.9999
**CTRL +αLAG3 vs. IFNγ +αCTLA4**	234.8	-86180 to 86649	No	ns	>0.9999
**CTRL +αLAG3 vs. IFNγ +αLAG3**	575.0	-85839 to 86989	No	ns	>0.9999
**CTRL +αLAG3 vs. IFNγ +1-MT**	477.3	-88517 to 89472	No	ns	>0.9999
**CTRL +1-MT vs. IFNγ isotype**	3452	-80976 to 87879	No	ns	>0.9999
**CTRL +1-MT vs. IFNγ +αCTLA4**	3776	-82638 to 90191	No	ns	>0.9999
**CTRL +1-MT vs. IFNγ +αLAG3**	4116	-82298 to 90531	No	ns	>0.9999
**CTRL +1-MT vs. IFNγ +1-MT**	4019	-84976 to 93013	No	ns	>0.9999
**IFNγ isotype vs. IFNγ +αCTLA4**	324.5	-71030 to 71679	No	ns	>0.9999
**IFNγ isotype vs. IFNγ +αLAG3**	664.6	-70690 to 72019	No	ns	>0.9999
**IFNγ isotype vs. IFNγ +1-MT**	567.0	-73891 to 75025	No	ns	>0.9999
**IFNγ +αCTLA4 vs. IFNγ +αLAG3**	340.2	-73354 to 74035	No	ns	>0.9999
**IFNγ +αCTLA4 vs. IFNγ +1-MT**	242.5	-76461 to 76946	No	ns	>0.9999
**IFNγ +αLAG3 vs. IFNγ +1-MT**	-97.70	-76801 to 76606	No	ns	>0.9999
**13**					
**CTRL isotype vs. CTRL +αCTLA4**	5646	-77838 to 89130	No	ns	>0.9999
**CTRL isotype vs. CTRL +αLAG3**	24145	-59340 to 107629	No	ns	0.9862
**CTRL isotype vs. CTRL +1-MT**	-3446	-92440 to 85549	No	ns	>0.9999
**CTRL isotype vs. IFNγ isotype**	27966	-46492 to 102425	No	ns	0.9415
**CTRL isotype vs. IFNγ +αCTLA4**	26828	-49875 to 103532	No	ns	0.9598
**CTRL isotype vs. IFNγ +αLAG3**	35471	-41233 to 112174	No	ns	0.8425
**CTRL isotype vs. IFNγ +1-MT**	30308	-49291 to 109907	No	ns	0.9373
**CTRL +αCTLA4 vs. CTRL +αLAG3**	18499	-68698 to 105695	No	ns	0.9979
**CTRL +αCTLA4 vs. CTRL +1-MT**	-9092	-101578 to 83394	No	ns	>0.9999
**CTRL +αCTLA4 vs. IFNγ isotype**	22321	-56277 to 100918	No	ns	0.9876
**CTRL +αCTLA4 vs. IFNγ +αCTLA4**	21182	-59546 to 101911	No	ns	0.9922
**CTRL +αCTLA4 vs. IFNγ +αLAG3**	29825	-50903 to 110553	No	ns	0.9463
**CTRL +αCTLA4 vs. IFNγ +1-MT**	24662	-58822 to 108146	No	ns	0.9844
**CTRL +αLAG3 vs. CTRL +1-MT**	-27591	-120076 to 64895	No	ns	0.9834
**CTRL +αLAG3 vs. IFNγ isotype**	3822	-74776 to 82420	No	ns	>0.9999
**CTRL +αLAG3 vs. IFNγ +αCTLA4**	2684	-78045 to 83412	No	ns	>0.9999
**CTRL +αLAG3 vs. IFNγ +αLAG3**	11326	-69402 to 92054	No	ns	0.9999
**CTRL +αLAG3 vs. IFNγ +1-MT**	6163	-77321 to 89647	No	ns	>0.9999
**CTRL +1-MT vs. IFNγ isotype**	31412	-53015 to 115840	No	ns	0.9443
**CTRL +1-MT vs. IFNγ +αCTLA4**	30274	-56140 to 116689	No	ns	0.9594
**CTRL +1-MT vs. IFNγ +αLAG3**	38917	-47498 to 125331	No	ns	0.8599
**CTRL +1-MT vs. IFNγ +1-MT**	33754	-55241 to 122748	No	ns	0.9385
**IFNγ isotype vs. IFNγ +αCTLA4**	-1138	-72493 to 70216	No	ns	>0.9999
**IFNγ isotype vs. IFNγ +αLAG3**	7504	-63850 to 78859	No	ns	>0.9999
**IFNγ isotype vs. IFNγ +1-MT**	2341	-72117 to 76799	No	ns	>0.9999
**IFNγ +αCTLA4 vs. IFNγ +αLAG3**	8643	-65052 to 82337	No	ns	>0.9999
**IFNγ +αCTLA4 vs. IFNγ +1-MT**	3479	-73224 to 80183	No	ns	>0.9999
**IFNγ +αLAG3 vs. IFNγ +1-MT**	-5163	-81867 to 71541	No	ns	>0.9999
**16**					
**CTRL isotype vs. CTRL +αCTLA4**	32436	-51048 to 115921	No	ns	0.9305
**CTRL isotype vs. CTRL +αLAG3**	109947	26463 to 193431	Yes	**	0.0022
**CTRL isotype vs. CTRL +1-MT**	25355	-87215 to 137925	No	ns	0.9970
**CTRL isotype vs. IFNγ isotype**	113970	37266 to 190673	Yes	***	0.0003
**CTRL isotype vs. IFNγ +αCTLA4**	106219	29516 to 182923	Yes	**	0.0010
**CTRL isotype vs. IFNγ +αLAG3**	183688	106984 to 260391	Yes	****	<0.0001
**CTRL isotype vs. IFNγ +1-MT**	192484	109000 to 275968	Yes	****	<0.0001
**CTRL +αCTLA4 vs. CTRL +αLAG3**	77511	-9686 to 164707	No	ns	0.1200
**CTRL +αCTLA4 vs. CTRL +1-MT**	-7082	-122432 to 108268	No	ns	>0.9999
**CTRL +αCTLA4 vs. IFNγ isotype**	81533	805.2 to 162262	Yes	*	0.0459
**CTRL +αCTLA4 vs. IFNγ +αCTLA4**	73783	-6945 to 154511	No	ns	0.0997
**CTRL +αCTLA4 vs. IFNγ +αLAG3**	151251	70523 to 231979	Yes	****	<0.0001
**CTRL +αCTLA4 vs. IFNγ +1-MT**	160048	72851 to 247244	Yes	****	<0.0001
**CTRL +αLAG3 vs. CTRL +1-MT**	-84592	-199942 to 30758	No	ns	0.3224
**CTRL +αLAG3 vs. IFNγ isotype**	4023	-76705 to 84751	No	ns	>0.9999
**CTRL +αLAG3 vs. IFNγ +αCTLA4**	-3728	-84456 to 77001	No	ns	>0.9999
**CTRL +αLAG3 vs. IFNγ +αLAG3**	73741	-6988 to 154469	No	ns	0.1001
**CTRL +αLAG3 vs. IFNγ +1-MT**	82537	-4659 to 169733	No	ns	0.0775
**CTRL +1-MT vs. IFNγ isotype**	88615	-21927 to 199157	No	ns	0.2168
**CTRL +1-MT vs. IFNγ +αCTLA4**	80865	-29677 to 191406	No	ns	0.3256
**CTRL +1-MT vs. IFNγ +αLAG3**	158333	47791 to 268874	Yes	***	0.0006
**CTRL +1-MT vs. IFNγ +1-MT**	167129	51779 to 282479	Yes	***	0.0005
**IFNγ isotype vs. IFNγ +αCTLA4**	-7750	-81445 to 65944	No	ns	>0.9999
**IFNγ isotype vs. IFNγ +αLAG3**	69718	-3977 to 143412	No	ns	0.0778
**IFNγ isotype vs. IFNγ +1-MT**	78514	-2214 to 159242	No	ns	0.0628
**IFNγ +αCTLA4 vs. IFNγ +αLAG3**	77468	3774 to 151163	Yes	*	0.0320
**IFNγ +αCTLA4 vs. IFNγ +1-MT**	86265	5536 to 166993	Yes	*	0.0273
**IFNγ +αLAG3 vs. IFNγ +1-MT**	8796	-71932 to 89525	No	ns	>0.9999

**Test details**	Predicted (LS) mean 1	Predicted (LS) mea	Predicted (LS) mea	SE of diff.	N1	N2	q	DF
**11**								
**CTRL isotype vs. CTRL +αCTLA4**	2612	1242	1370	27039	6	5	0.07166	115.0
**CTRL isotype vs. CTRL +αLAG3**	2612	679.5	1932	28824	6	4	0.09480	115.0
**CTRL isotype vs. CTRL +1-MT**	2612	4221	-1609	28824	6	4	0.07895	115.0
**CTRL isotype vs. IFNγ isotype**	2612	769.2	1843	24116	6	8	0.1080	115.0
**CTRL isotype vs. IFNγ +αCTLA4**	2612	444.7	2167	24843	6	7	0.1234	115.0
**CTRL isotype vs. IFNγ +αLAG3**	2612	104.5	2507	24843	6	7	0.1427	115.0
**CTRL isotype vs. IFNγ +1-MT**	2612	202.2	2409	25781	6	6	0.1322	115.0
**CTRL +αCTLA4 vs. CTRL +αLAG3**	1242	679.5	562.1	29955	5	4	0.02654	115.0
**CTRL +αCTLA4 vs. CTRL +1-MT**	1242	4221	-2979	29955	5	4	0.1407	115.0
**CTRL +αCTLA4 vs. IFNγ isotype**	1242	769.2	472.4	25457	5	8	0.02625	115.0
**CTRL +αCTLA4 vs. IFNγ +αCTLA4**	1242	444.7	796.9	26147	5	7	0.04310	115.0
**CTRL +αCTLA4 vs. IFNγ +αLAG3**	1242	104.5	1137	26147	5	7	0.06150	115.0
**CTRL +αCTLA4 vs. IFNγ +1-MT**	1242	202.2	1039	27039	5	6	0.05436	115.0
**CTRL +αLAG3 vs. CTRL +1-MT**	679.5	4221	-3541	31575	4	4	0.1586	115.0
**CTRL +αLAG3 vs. IFNγ isotype**	679.5	769.2	-89.68	27345	4	8	0.004638	115.0
**CTRL +αLAG3 vs. IFNγ +αCTLA4**	679.5	444.7	234.8	27988	4	7	0.01186	115.0
**CTRL +αLAG3 vs. IFNγ +αLAG3**	679.5	104.5	575.0	27988	4	7	0.02905	115.0
**CTRL +αLAG3 vs. IFNγ +1-MT**	679.5	202.2	477.3	28824	4	6	0.02342	115.0
**CTRL +1-MT vs. IFNγ isotype**	4221	769.2	3452	27345	4	8	0.1785	115.0
**CTRL +1-MT vs. IFNγ +αCTLA4**	4221	444.7	3776	27988	4	7	0.1908	115.0
**CTRL +1-MT vs. IFNγ +αLAG3**	4221	104.5	4116	27988	4	7	0.2080	115.0
**CTRL +1-MT vs. IFNγ +1-MT**	4221	202.2	4019	28824	4	6	0.1972	115.0
**IFNγ isotype vs. IFNγ +αCTLA4**	769.2	444.7	324.5	23111	8	7	0.01985	115.0
**IFNγ isotype vs. IFNγ +αLAG3**	769.2	104.5	664.6	23111	8	7	0.04067	115.0
**IFNγ isotype vs. IFNγ +1-MT**	769.2	202.2	567.0	24116	8	6	0.03325	115.0
**IFNγ +αCTLA4 vs. IFNγ +αLAG3**	444.7	104.5	340.2	23869	7	7	0.02016	115.0
**IFNγ +αCTLA4 vs. IFNγ +1-MT**	444.7	202.2	242.5	24843	7	6	0.01380	115.0
**IFNγ +αLAG3 vs. IFNγ +1-MT**	104.5	202.2	-97.70	24843	7	6	0.005561	115.0
**13**								
**CTRL isotype vs. CTRL +αCTLA4**	42343	36697	5646	27039	6	5	0.2953	115.0
**CTRL isotype vs. CTRL +αLAG3**	42343	18198	24145	27039	6	5	1.263	115.0
**CTRL isotype vs. CTRL +1-MT**	42343	45789	-3446	28824	6	4	0.1691	115.0
**CTRL isotype vs. IFNγ isotype**	42343	14376	27966	24116	6	8	1.640	115.0
**CTRL isotype vs. IFNγ +αCTLA4**	42343	15515	26828	24843	6	7	1.527	115.0
**CTRL isotype vs. IFNγ +αLAG3**	42343	6872	35471	24843	6	7	2.019	115.0
**CTRL isotype vs. IFNγ +1-MT**	42343	12035	30308	25781	6	6	1.663	115.0
**CTRL +αCTLA4 vs. CTRL +αLAG3**	36697	18198	18499	28242	5	5	0.9263	115.0
**CTRL +αCTLA4 vs. CTRL +1-MT**	36697	45789	-9092	29955	5	4	0.4292	115.0
**CTRL +αCTLA4 vs. IFNγ isotype**	36697	14376	22321	25457	5	8	1.240	115.0
**CTRL +αCTLA4 vs. IFNγ +αCTLA4**	36697	15515	21182	26147	5	7	1.146	115.0
**CTRL +αCTLA4 vs. IFNγ +αLAG3**	36697	6872	29825	26147	5	7	1.613	115.0
**CTRL +αCTLA4 vs. IFNγ +1-MT**	36697	12035	24662	27039	5	6	1.290	115.0
**CTRL +αLAG3 vs. CTRL +1-MT**	18198	45789	-27591	29955	5	4	1.303	115.0
**CTRL +αLAG3 vs. IFNγ isotype**	18198	14376	3822	25457	5	8	0.2123	115.0
**CTRL +αLAG3 vs. IFNγ +αCTLA4**	18198	15515	2684	26147	5	7	0.1451	115.0
**CTRL +αLAG3 vs. IFNγ +αLAG3**	18198	6872	11326	26147	5	7	0.6126	115.0
**CTRL +αLAG3 vs. IFNγ +1-MT**	18198	12035	6163	27039	5	6	0.3223	115.0
**CTRL +1-MT vs. IFNγ isotype**	45789	14376	31412	27345	4	8	1.625	115.0
**CTRL +1-MT vs. IFNγ +αCTLA4**	45789	15515	30274	27988	4	7	1.530	115.0
**CTRL +1-MT vs. IFNγ +αLAG3**	45789	6872	38917	27988	4	7	1.966	115.0
**CTRL +1-MT vs. IFNγ +1-MT**	45789	12035	33754	28824	4	6	1.656	115.0
**IFNγ isotype vs. IFNγ +αCTLA4**	14376	15515	-1138	23111	8	7	0.06965	115.0
**IFNγ isotype vs. IFNγ +αLAG3**	14376	6872	7504	23111	8	7	0.4592	115.0
**IFNγ isotype vs. IFNγ +1-MT**	14376	12035	2341	24116	8	6	0.1373	115.0
**IFNγ +αCTLA4 vs. IFNγ +αLAG3**	15515	6872	8643	23869	7	7	0.5121	115.0
**IFNγ +αCTLA4 vs. IFNγ +1-MT**	15515	12035	3479	24843	7	6	0.1981	115.0
**IFNγ +αLAG3 vs. IFNγ +1-MT**	6872	12035	-5163	24843	7	6	0.2939	115.0
**16**								
**CTRL isotype vs. CTRL +αCTLA4**	235569	203133	32436	27039	6	5	1.696	115.0
**CTRL isotype vs. CTRL +αLAG3**	235569	125622	109947	27039	6	5	5.750	115.0
**CTRL isotype vs. CTRL +1-MT**	235569	210214	25355	36460	6	2	0.9835	115.0
**CTRL isotype vs. IFNγ isotype**	235569	121599	113970	24843	6	7	6.488	115.0
**CTRL isotype vs. IFNγ +αCTLA4**	235569	129350	106219	24843	6	7	6.047	115.0
**CTRL isotype vs. IFNγ +αLAG3**	235569	51881	183688	24843	6	7	10.46	115.0
**CTRL isotype vs. IFNγ +1-MT**	235569	43085	192484	27039	6	5	10.07	115.0
**CTRL +αCTLA4 vs. CTRL +αLAG3**	203133	125622	77511	28242	5	5	3.881	115.0
**CTRL +αCTLA4 vs. CTRL +1-MT**	203133	210214	-7082	37360	5	2	0.2681	115.0
**CTRL +αCTLA4 vs. IFNγ isotype**	203133	121599	81533	26147	5	7	4.410	115.0
**CTRL +αCTLA4 vs. IFNγ +αCTLA4**	203133	129350	73783	26147	5	7	3.991	115.0
**CTRL +αCTLA4 vs. IFNγ +αLAG3**	203133	51881	151251	26147	5	7	8.181	115.0
**CTRL +αCTLA4 vs. IFNγ +1-MT**	203133	43085	160048	28242	5	5	8.014	115.0
**CTRL +αLAG3 vs. CTRL +1-MT**	125622	210214	-84592	37360	5	2	3.202	115.0
**CTRL +αLAG3 vs. IFNγ isotype**	125622	121599	4023	26147	5	7	0.2176	115.0
**CTRL +αLAG3 vs. IFNγ +αCTLA4**	125622	129350	-3728	26147	5	7	0.2016	115.0
**CTRL +αLAG3 vs. IFNγ +αLAG3**	125622	51881	73741	26147	5	7	3.988	115.0
**CTRL +αLAG3 vs. IFNγ +1-MT**	125622	43085	82537	28242	5	5	4.133	115.0
**CTRL +1-MT vs. IFNγ isotype**	210214	121599	88615	35803	2	7	3.500	115.0
**CTRL +1-MT vs. IFNγ +αCTLA4**	210214	129350	80865	35803	2	7	3.194	115.0
**CTRL +1-MT vs. IFNγ +αLAG3**	210214	51881	158333	35803	2	7	6.254	115.0
**CTRL +1-MT vs. IFNγ +1-MT**	210214	43085	167129	37360	2	5	6.326	115.0
**IFNγ isotype vs. IFNγ +αCTLA4**	121599	129350	-7750	23869	7	7	0.4592	115.0
**IFNγ isotype vs. IFNγ +αLAG3**	121599	51881	69718	23869	7	7	4.131	115.0
**IFNγ isotype vs. IFNγ +1-MT**	121599	43085	78514	26147	7	5	4.247	115.0
**IFNγ +αCTLA4 vs. IFNγ +αLAG3**	129350	51881	77468	23869	7	7	4.590	115.0
**IFNγ +αCTLA4 vs. IFNγ +1-MT**	129350	43085	86265	26147	7	5	4.666	115.0
**IFNγ +αLAG3 vs. IFNγ +1-MT**	51881	43085	8796	26147	7	5	0.4758	115.0

Appendix Table S6
Appendix Table S6

Collection and histopathology evaluation of tissues from wild-type mice treated with Hematopoietic Stem Cells (HSCs) transduced with a Tie2.Ifn2.Ifntiviral plasmid

Author:	Signature:	Date:
Francesca Sanvito<br>GLP SR-TIGET<br>Responsible Scientist &<br>Study Pathologist	Francesca Sanvito	08 June 2021

MANAGEMENT:	Signature:	Date:
Paola Albertini<br>GLP SR-TIGET<br>Management	Paola Albertini	08 June 2021

TABLE OF CONTENTS

1. DETAILS OF TEST FACILITY AND PERSONNEL 3
2. INTRODUCTION 4
3. DEFINITION AND ABBREVIATION 4
4. REGULATORY COMPLIANCE 4
5. MATERIALS AND METHODS 4
5.1. Study Design 4
5.2. Treatment Groups 4
5.3. Necropsy and histology 5
5.4. Pathology 5
6. RESULTS 5
6.1. Mortality/ICDs: Intercurrent deaths 5
6.2. Terminal Kill 5
6.2.1. Organ weights and macroscopic findings 5
6.2.2. Microscopic findings 6
7. CONCLUSION 7
8. REFERENCES 7
Appendix 8

1. DETAILS OF TEST FACILITY AND PERSONNEL

**Test Facility**	GLP SR-TIGET<br>Ospedale San Raffaele,<br>Via Olgettina, 60<br>20132 Milan, Italy
**Responsible Scientist and Study Pathologist**	Francesca Sanvito<br>GLP SR-TIGET
**Reference Scientist**	Giacomo Desantis<br>SR-TIGET
**Scientific Project Responsible**	Bernhard Gentner<br>SR-TIGET
**Personnel involved**	GLP SR-TIGET Test Facility personnel:<br>Michela Vezzoli<br>Maura De Simone<br>Ilaria Visigalli<br>Francesca Cecere<br>Raisa Jofra Hernandez<br>Valentina Mauro<br>Rossana Norata<br>Rocchi Martina

2. INTRODUCTION

The purpose of this study is the necropsy, macroscopic observations, organs collection, and histopathological analysis to evaluate the safety of anti-tumor myeloid-based gene therapy achieved by transplantation of Hematopoietic Stem Cells (HSCs) transduced with a Tie2.Ifn2.Ifntiviral plasmid. The plasmid encodes for the murine Ifng gene under regulation of the murine Tie2 gene promoter.

3. DEFINITION AND ABBREVIATION

Table of abbreviations in the Incidence Tables of Microscopic Findings_interim report	
**Depos pigment hepato**	Deposition: tubular epithelium - cortex,
**Extramed hematopoie**	Extramedullary hematopoiesis
**IHC**	Immunohistochemistry

4. REGULATORY COMPLIANCE

No claim for compliance with GLP is made for this study although the work has been performed following Good Laboratory Practices principles and applicable SOPs.

5. MATERIALS AND METHODS

5.1. Study Design

Female Ly45.2 C57BL/6N mice were transplanted either with 0.828x10^6 Ly45.1Lin⁻ cells transduced with a Tie2.Ifnγ plasmid (Test Item, Group2) or with 0.843x10^6 Tie2.Ngfr plasmid (Control Item, Group1) after irradiation regimen (850 cGy) at 8-week age. Animals were monitored for 12 weeks.

5.2. Treatment Groups

Group Code	Group Name	Ascentos ID Group	Conditioning	Transplanted Cells	Sex	N°	Mouse ID (RD Path 126)
1	Tie2.Ngfr	1	850 cGy	Tie2.Ngfr-transduced Ly45.1 Lin⁻ cells	F	6	001-006
2	Tie2.Ifnγ	2	850 cGy	Tie2.Ifnγ-transduced Ly45.1 Lin⁻ cells	F	6	007-012

F: female

5.3. NECROPSY AND HISTOLOGY

A full necropsy was performed and gross observations were recorded from all terminal killed animals at 12 weeks post infusion, for Group 1 and Group 2. Bone marrow (right femur, tibia, and sternum), brain, heart, kidneys, liver (left and right, medial right, medial left,) with gall bladder, lungs, mesenteric lymph nodes, spleen, thymus, and any macroscopic abnormalities were collected as for study plan. Selected organs, heart, lungs, liver, kidneys spleen and thymus were dissected free of fat and weighted. Tissues were processed, embedded in paraffin, sectioned, and stained with haematoxylin and eosin for histological examination. Decalcification was performed for femur, tibia and sternum.

Blocks and slides will be identified as follows: Study N.-group-animal ID.

Immunohistochemical analysis (IHC) using anti CD3, anti CD45R/B220, anti F4/80 and anti IBA1 was performed on thymus of animal R&D PATH126-2-009.

5.4. PATHOLOGY

Necropsy, histology and microscopic evaluation of tissues were performed following GLP SR-TIGET SOPs and as for study plan. Terminal body weight, organ weights, macroscopic and microscopic observations were recorded using a dedicated computerized system (Ascentos l.3.3, PDS Ltd).

Microscopic lesions were graded on a scale of 1 to 5 as minimal, mild, moderate, marked, or severe; minimal referred to the least extent discernible and severe the greatest extent possible. The assessment of the histopathology included an informal blinding step. The slides were reviewed unblinded, to determine the features of the pathology relevant to the disease process and the range of severity of changes relative to the controls. The slides (including controls) were then informally randomised (shuffled), and relevant pathological features scored without information on the treatment groups.

6. RESULTS

6.1. MORTALITY/ICDs: INTERCURRENT DEATHS

No mortality was recorded.

6.2. TERMINAL KILL

6.2.1. ORGAN WEIGHTS AND MACROSCOPIC FINDINGS

(Appendix Table 1, Table 2 and Pathology Individual Animal Data)

Terminal body weight, individual organ weights and group mean data are recorded in Table 1 and Table 2. Absolute organ weights and relative organ weights calculated on terminal body weight and on brain weight are represented. No difference in terminal body weights and organ weights were observed in animals between Group 1 and Group 2. The slight difference in lung weights between groups is due to the expected variability of the organ. No macroscopic or microscopic changes were observed.

No macroscopic observations were recorded in animals of both Group 1 and Group 2.

6.2.2. MICROSCOPIC FINDINGS

(Appendix: Table 3 and Pathology Individual Animal Data)

Spleen

Minimal to mild extramedullary hematopoiesis was detected in all mice across both groups, without difference in severity.

Thymus

Mouse R&D PATH 126-2-009 of Group 2 showed T cell lymphoblastic lymphoma. Lymphoma was localized in one lobe and showed diffused CD3 positivity (T cell marker) by immunohistochemistry. Other IHC markers, CD45R/B220 (B cells), anti F4/8 and anti IBA1 were negative in neoplastic cells.

No microscopic changes were seen in the thymus of the other four mice examined in the Group 2 and in the six of the Group 1.

Legend of images: neoplastic lymphocytes have scant cytoplasm and round nuclei. The nuclei have a fine chromatin pattern with either centralized or inconspicuous nucleoli. Mitoses are frequent, and there are scattered tingible-body macrophages.

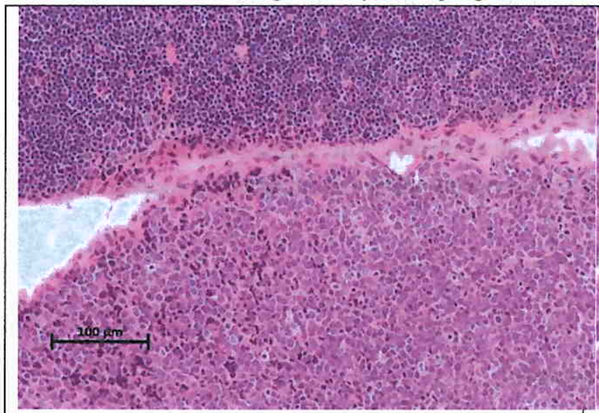

R&DPATH126-2-009 Thymus Lymphoma. H&E, bar: 100 micron

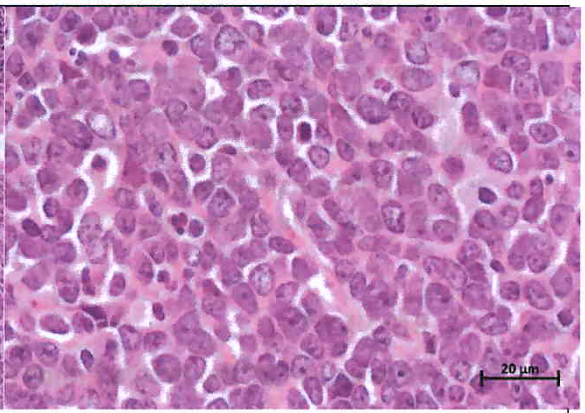

R&DPATH126-2-009 Thymus Lymphoma. H&E, bar: 20 micron

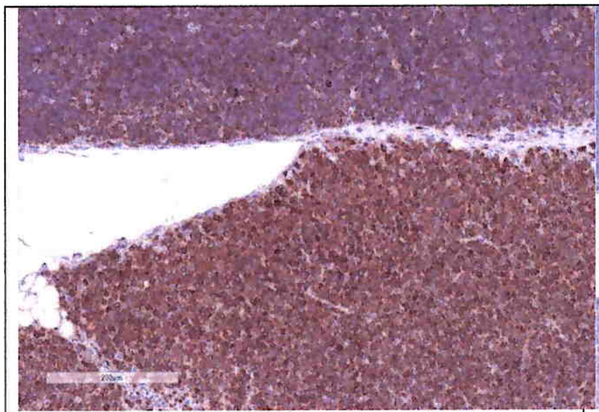	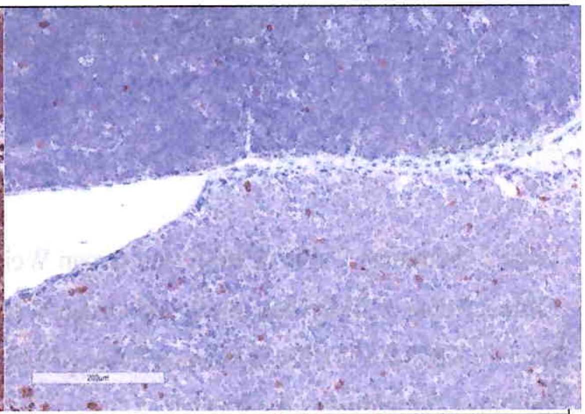
R&DPATH126-2-009 Thymus Lymphoma. CD3, bar: 200 micron	R&DPATH126-2-009 Thymus Lymphoma. CD45R/B220, bar: 200 micron

Liver

Mouse R&D PATH 126-2-009 showed a focal minimal deposition of pigment in few hepatocytes.

Bone marrow, brain, heart, kidneys, gall bladder, lungs, and mesenteric lymph nodes

No findings were recorded in mice of both groups.

7. CONCLUSION

The increased extramedullary hematopoiesis seen in test item treated and control animals represents an expected finding in the spleen of irradiated female mice. One animal transplanted with Hematopoietic Stem Cells (HSCs) transduced with a Tie2.Ifn2.Ifntiviral plasmid showed localised T cell lymphoblastic lymphoma in thymus. No correlated microscopic findings were seen in other organs nor in the other mice transplanted with the test item. Lymphoma is a spontaneous tumor in mice and the development is enhanced and accelerated by irradiation. Therefore, the occurrence in this study is inconclusive and considered to be incidental (Vesselinovitch, 1971; Ward, 2006; Upton et al., 1960).

8. REFERENCES

Vesselinovitch SD, Simmons EL, Mihailovich N, Rao KVN, and Lombard LS. The effect of age, fractionation, and dose on radiation carcinogenesis in various tissues of mice. *Cancer Res* 1971; 31: 2133-42.

Ward JM Lymphomas and leukemias in mice *Exp Toxicol Pathol.* 2006, 57 (5-6): 377-81.

Upton A.C., Kimball A.W. and Furth J. Some Delayed Effects of Atom-Bomb Radiations in Mice. *Cancer Res* 1960; 20: 1-60.

APPENDIX

Table 1. Summary Table of Body and Organ Weights and Statistics pag. 9

Table 2. Table of Individual Body and Organ Weights pag. 12

Table 3. R&D PATH 126 Incidence Table of Microscopic Findings pag. 14

Pathology Individual Animal Data pag. 16

Collection and histopathology evaluation of tissues from wild-type mice treated with Hematopoietic Stem Cells (HSCs) transduced with a Tie2.Ifn2.Ifntiviral plasmid.

Table 1

Summary Table of Body and Organ Weights and Statistics
Status at Necropsy: K0

		Tie2.Ngfr	Tie2.Ifngamma
Groups		Tie2.Ngfr	Tie2.Ifngamma
Sex		F	F
No. Animals		6	6
FINAL BODY WEIGHT	FINAL BODY WEIGHT		
	Number of weights	6	6
	Mean weight [g]	20.8	20.8
	Standard Deviation [g]	1.5	1.2
BRAIN	BRAIN		
	Number of weights	6	6
	Mean weight [g]	0.48	0.46
	Standard Deviation [g]	0.08	0.06
	Mean Organ/Body [%]	2.30	2.23
	Standard Deviation [%]	0.26	0.38
HEART	HEART		
	Number of weights	6	6
	Mean weight [g]	0.13	0.12
	Standard Deviation [g]	0.02	0.01
	Mean Organ/Body [%]	0.61	0.60
	Standard Deviation [%]	0.10	0.08
	Mean Organ/Brain [%]	27.04	26.82
	Standard Deviation [%]	6.34	1.07
LUNG	LUNG		
	Number of weights	6	6
	Mean weight [g]	0.33	0.36
	Standard Deviation [g]	0.02	0.11
	Mean Organ/Body [%]	1.58	1.75
	Standard Deviation [%]	0.14	0.60
	Mean Organ/Brain [%]	69.31	79.55
	Standard Deviation [%]	8.56	27.92
LIVER	LIVER		
	Number of weights	6	6
	Mean weight [g]	1.19	1.19
	Standard Deviation [g]	0.12	0.10
	Mean Organ/Body [%]	5.73	5.70
	Standard Deviation [%]	0.29	0.23
	Mean Organ/Brain [%]	250.44	260.81
	Standard Deviation [%]	23.41	42.15

Collection and histopathology evaluation of tissues from wild-type mice treated with Hematopoietic Stem Cells (HSCs) transduced with a Tie2.Ifn2.Ifntiviral plasmid.

Table 1

Summary Table of Body and Organ Weights and Statistics
Status at Necropsy: K0

		Tie2.Ngfr	Tie2.Ifngamma
Groups		Tie2.Ngfr	Tie2.Ifngamma
Sex		F	F
No. Animals		6	6
KIDNEYS	KIDNEYS		
	Number of weights	6	6
	Mean weight [g]	0.27	0.27
	Standard Deviation [g]	0.01	0.02
	Mean Organ/Body [%]	1.31	1.28
	Standard Deviation [%]	0.07	0.07
	Mean Organ/Brain [%]	57.58	58.63
	Standard Deviation [%]	8.14	8.41
SPLEEN	SPLEEN		
	Number of weights	6	6
	Mean weight [g]	0.09	0.10
	Standard Deviation [g]	0.02	0.02
	Mean Organ/Body [%]	0.46	0.47
	Standard Deviation [%]	0.10	0.06
	Mean Organ/Brain [%]	20.19	21.43
	Standard Deviation [%]	5.41	4.43
THYMUS	THYMUS		
	Number of weights	6	6
	Mean weight [g]	0.05	0.04
	Standard Deviation [g]	0.02	0.01
	Mean Organ/Body [%]	0.26	0.22
	Standard Deviation [%]	0.09	0.04
	Mean Organ/Brain [%]	11.75	9.93
	Standard Deviation [%]	4.90	2.60

Collection and histopathology evaluation of tissues from wild-type mice treated with Hematopoietic Stem Cells (HSCs) transduced with a Tie2.Ifn2.Ifntiviral plasmid.

Table 1

Summary Table of Body and Organ Weights and Statistics

Collection and histopathology evaluation of tissues from wild-type mice treated with Hematopoietic Stem Cells (HSCs) transduced with a Tie2.Ifn2.Ifntiviral plasmid.

Table 2

Table of Individual Body and Organ Weights
Dose Group: Tie2.Ngfr, Control
Sex: Female

Animal Number / Days on Test / Date of Necropsy		RDPAT H126-1-001	RDPAT H126-1-002	RDPAT H126-1-003	RDPAT H126-1-004	RDPAT H126-1-005	RDPAT H126-1-006
Days on Test		83	83	83	84	84	84
Date of Necropsy		05-May-21	05-May-21	05-May-21	06-May-21	06-May-21	06-May-21
Defined/Actual Necropsy Status		K0/K0	K0/K0	K0/K0	K0/K0	K0/K0	K0/K0
FINAL BODY WEIGHT							
	Total [g]	21.0	20.1	20.3	21.9	18.6	22.9
BRAIN							
	Total [g]	0.47	0.46	0.42	0.47	0.43	0.64
	Organ/Body [%]	2.23	2.29	2.06	2.13	2.31	2.81
HEART							
	Total [g]	0.15	0.12	0.15	0.12	0.11	0.11
	Organ/Body [%]	0.70	0.58	0.75	0.53	0.61	0.50
	Organ/Brain [%]	31.23	25.50	36.49	24.89	26.41	17.73
LUNG							
	Total [g]	0.33	0.34	0.29	0.34	0.33	0.34
	Organ/Body [%]	1.55	1.70	1.42	1.54	1.80	1.50
	Organ/Brain [%]	69.18	74.35	68.87	72.32	77.88	53.24
LIVER							
	Total [g]	1.20	1.09	1.21	1.18	1.07	1.40
	Organ/Body [%]	5.69	5.43	5.95	5.38	5.77	6.14
	Organ/Brain [%]	254.90	237.52	289.50	252.05	250.23	218.43
KIDNEYS							
	Total [g]	0.26	0.27	0.29	0.28	0.25	0.28
	Organ/Body [%]	1.26	1.34	1.43	1.27	1.33	1.24
	Organ/Brain [%]	56.26	58.57	69.39	59.44	57.82	43.97
SPLEEN							
	Total [g]	0.09	0.07	0.12	0.09	0.11	0.09
	Organ/Body [%]	0.44	0.37	0.57	0.39	0.59	0.39
	Organ/Brain [%]	19.84	16.10	27.54	18.21	25.69	13.78
THYMUS							
	Total [g]	0.05	0.04	0.09	0.05	0.04	0.05
	Organ/Body [%]	0.24	0.21	0.44	0.23	0.22	0.24
	Organ/Brain [%]	10.77	9.40	21.61	10.62	9.59	8.49

Collection and histopathology evaluation of tissues from wild-type mice treated with Hematopoietic Stem Cells (HSCs) transduced with a Tie2.Ifn2.Ifntiviral plasmid.

Table 2

Table of Individual Body and Organ Weights
Dose Group: Tie2.Ifngamma, Treated
Sex: Female

	Animal Number	RDPATH126-2-007	RDPATH126-2-008	RDPATH126-2-009	RDPATH126-2-010	RDPATH126-2-011	RDPATH126-2-012
	Days on Test	83	83	83	84	84	84
	Date of Necropsy	05-May-21	05-May-21	05-May-21	06-May-21	06-May-21	06-May-21
	Defined/Actual Necropsy Status	K0/K0	K0/K0	K0/K0	K0/K0	K0/K0	K0/K0
FINAL	FINAL BODY WEIGHT						
	Total [g]	21.3	19.3	21.9	20.9	21.9	19.5
BRAIN	BRAIN						
	Total [g]	0.42	0.43	0.46	0.45	0.44	0.58
	Organ/Body [%]	1.97	2.21	2.10	2.14	2.01	2.98
HEART	HEART						
	Total [g]	0.12	0.12	0.12	0.12	0.12	0.15
	Organ/Body [%]	0.55	0.61	0.56	0.55	0.56	0.76
	Organ/Brain [%]	27.92	27.60	26.56	25.80	27.69	25.38
LUNG	LUNG						
	Total [g]	0.30	0.55	0.27	0.30	0.41	0.34
	Organ/Body [%]	1.40	2.87	1.22	1.43	1.86	1.72
	Organ/Brain [%]	71.34	130.19	58.00	67.05	92.82	57.91
LIVER	LIVER						
	Total [g]	1.27	1.05	1.23	1.17	1.31	1.09
	Organ/Body [%]	5.96	5.44	5.60	5.59	6.01	5.59
	Organ/Brain [%]	303.08	246.54	266.58	261.37	299.64	187.67
KIDNEYS	KIDNEYS						
	Total [g]	0.26	0.26	0.26	0.28	0.29	0.25
	Organ/Body [%]	1.21	1.35	1.18	1.34	1.35	1.28
	Organ/Brain [%]	61.63	61.06	56.18	62.67	67.21	43.06
SPLEEN	SPLEEN						
	Total [g]	0.11	0.07	0.11	0.11	0.10	0.09
	Organ/Body [%]	0.50	0.35	0.49	0.53	0.47	0.47
	Organ/Brain [%]	25.27	15.65	23.45	24.73	23.58	15.92
THYMUS	THYMUS						
	Total [g]	0.06	0.05	0.04	0.05	0.04	0.04
	Organ/Body [%]	0.28	0.23	0.17	0.25	0.17	0.21
	Organ/Brain [%]	13.98	10.59	8.10	11.53	8.37	6.99

Collection and histopathology evaluation of tissues from wild-type mice treated with Hematopoietic Stem Cells (HSCs) transduced with a Tie2.Ifn2.Ifntiviral plasmid.

Table 3. R&DPATH126 Incidence Table of Microscopic Findings

Number of Animals with Microscopic Findings by Organ/Group/Sex, Grade 0..5
Status at Necropsy: K0 Combined, incl. Decedents

	Groups	Tie2.Ngfr	Tie2.Ifngamma
	Sex	F	F
	No. Animals	6	6
BRAIN			
	NO. EXAM.	6	6
	NAD	6	6
HEART			
	NO. EXAM.	6	6
	NAD	6	6
LUNG			
	NO. EXAM.	6	6
	NAD	6	6
LIVER LIVER			
	NO. EXAM.	6	6
	NAD	6	5
Depos pigment hepato		0	1
Grade: 1		0	1
GALLBLADDER			
	NO. EXAM.	6	6
	NAD	6	6
KIDNEYS			
	NO. EXAM.	6	6
	NAD	6	6
SPLEEN SPLEEN			
	NO. EXAM.	6	6
	NAD	0	0
Extramed hematopoie		6	6
Grade: 1		4	4
Grade: 2		2	2
BONE MARROW, STERNUM			
	NO. EXAM.	6	6
	NAD	6	6
BONE MARROW, FEMUR			
	NO. EXAM.	6	6
	NAD	6	6
BONE MARROW, TIBIA			
	NO. EXAM.	6	6
	NAD	6	6

NO.EXAM. = Number of animals examined
NAD = Nothing Abnormal Discovered

Collection and histopathology evaluation of tissues from wild-type mice treated with Hematopoietic Stem Cells (HSCs) transduced with a Tie2.Ifn2.Ifntiviral plasmid.

Table 3. R&DPATH126 Incidence Table of Microscopic Findings

Number of Animals with Microscopic Findings by Organ/Group/Sex, Grade 0..5
Status at Necropsy: K0 Combined, incl. Decedents

		Groups	Tie2.Ngfr	Tie2.Ifngamma
		Sex	F	F
		No. Animals	6	6
THYMUS	THYMUS			
		NO. EXAM.	6	6
		NAD	6	5
	Lymphoma		0	1
	MESENT. LYMPH NODE			
		NO. EXAM.	6	6
		NAD	6	6
SYSTEMIC NEOPLASM	SYSTEMIC NEOPLASMS			
		NO. EXAM.	0	1
		NAD	-	0
	Lymphoma malignant		-	1

NO.EXAM. = Number of animals examined
NAD = Nothing Abnormal Discovered

Collection and histopathology evaluation of tissues from wild-type mice treated with Hematopoietic Stem Cells (HSCs) transduced with a Tie2.Ifn2.Ifntiviral plasmid.

R&D PATH 126. Pathology Individual Animal Data

ANIMAL NUMBER	:	RDPATH126 SEX: Female -1-001	DOSE GROUP: 1
Defined Sacrifice Group	:	K0	
State at Necropsy	:	Terminal sacrifice	
Date of Necropsy	:	05-May-2021	
Days on Test	:	83 (12-Feb-2021 - 05-May-2021)	

* MACROSCOPIC OBSERVATIONS
No macroscopic observations noted

* MICROSCOPIC FINDINGS
SPLEEN
-Extramedullary hematopoiesis, minimal
All other protocol tissues without pathologic findings

ANIMAL NUMBER	:	RDPATH126 SEX: Female -1-002	DOSE GROUP: 1
Defined Sacrifice Group	:	K0	
State at Necropsy	:	Terminal sacrifice	
Date of Necropsy	:	05-May-2021	
Days on Test	:	83 (12-Feb-2021 - 05-May-2021)	

* MACROSCOPIC OBSERVATIONS
No macroscopic observations noted

* MICROSCOPIC FINDINGS
SPLEEN
-Extramedullary hematopoiesis, minimal
All other protocol tissues without pathologic findings

ANIMAL NUMBER	:	RDPATH126 SEX: Female -1-003	DOSE GROUP: 1
Defined Sacrifice Group	:	K0	
State at Necropsy	:	Terminal sacrifice	
Date of Necropsy	:	05-May-2021	
Days on Test	:	83 (12-Feb-2021 - 05-May-2021)	

* MACROSCOPIC OBSERVATIONS
No macroscopic observations noted

* MICROSCOPIC FINDINGS
SPLEEN
-Extramedullary hematopoiesis, mild
All other protocol tissues without pathologic findings

ANIMAL NUMBER	:	RDPATH126 SEX: Female -1-004	DOSE GROUP: 1
Defined Sacrifice Group	:	K0	
State at Necropsy	:	Terminal sacrifice	
Date of Necropsy	:	06-May-2021	
Days on Test	:	84 (12-Feb-2021 - 06-May-2021)	

Collection and histopathology evaluation of tissues from wild-type mice treated with Hematopoietic Stem Cells (HSCs) transduced with a Tie2.Ifn2.Ifntiviral plasmid.

R&D PATH 126. Pathology Individual Animal Data

Continued Animal: RDPATH126-1-004

* MACROSCOPIC OBSERVATIONS
No macroscopic observations noted

* MICROSCOPIC FINDINGS
SPLEEN
-Extramedullary hematopoiesis, minimal
All other protocol tissues without pathologic findings

ANIMAL NUMBER	:	RDPATH126 -1-005 SEX: Female	DOSE GROUP: 1
Defined Sacrifice Group	:	K0	
State at Necropsy	:	Terminal sacrifice	
Date of Necropsy	:	06-May-2021	
Days on Test	:	84 (12-Feb-2021 - 06-May-2021)	

* MACROSCOPIC OBSERVATIONS
No macroscopic observations noted

* MICROSCOPIC FINDINGS
SPLEEN
-Extramedullary hematopoiesis, mild
All other protocol tissues without pathologic findings

ANIMAL NUMBER	:	RDPATH126 -1-006 SEX: Female	DOSE GROUP: 1
Defined Sacrifice Group	:	K0	
State at Necropsy	:	Terminal sacrifice	
Date of Necropsy	:	06-May-2021	
Days on Test	:	84 (12-Feb-2021 - 06-May-2021)	

* MACROSCOPIC OBSERVATIONS
No macroscopic observations noted

* MICROSCOPIC FINDINGS
SPLEEN
-Extramedullary hematopoiesis, minimal
All other protocol tissues without pathologic findings

ANIMAL NUMBER	:	RDPATH126 -2-007 SEX: Female	DOSE GROUP: 2
Defined Sacrifice Group	:	K0	
State at Necropsy	:	Terminal sacrifice	
Date of Necropsy	:	05-May-2021	
Days on Test	:	83 (12-Feb-2021 - 05-May-2021)	

* MACROSCOPIC OBSERVATIONS
No macroscopic observations noted

Collection and histopathology evaluation of tissues from wild-type mice treated with Hematopoietic Stem Cells (HSCs) transduced with a Tie2.Ifn2.Ifntiviral plasmid.

R&D PATH 126. Pathology Individual Animal Data

Continued Animal: RDPATH126-2-007

* MICROSCOPIC FINDINGS
SPLEEN
-Extramedullary hematopoiesis, minimal
All other protocol tissues without pathologic findings

ANIMAL NUMBER	:	RDPATH126 SEX: Female -2-008	DOSE GROUP: 2
Defined Sacrifice Group	:	K0	
State at Necropsy	:	Terminal sacrifice	
Date of Necropsy	:	05-May-2021	
Days on Test	:	83 (12-Feb-2021 - 05-May-2021)	

* MACROSCOPIC OBSERVATIONS
No macroscopic observations noted

* MICROSCOPIC FINDINGS
SPLEEN
-Extramedullary hematopoiesis, minimal
All other protocol tissues without pathologic findings

ANIMAL NUMBER	:	RDPATH126 SEX: Female -2-009	DOSE GROUP: 2
Defined Sacrifice Group	:	K0	
State at Necropsy	:	Terminal sacrifice	
Date of Necropsy	:	05-May-2021	
Days on Test	:	83 (12-Feb-2021 - 05-May-2021)	

* MACROSCOPIC OBSERVATIONS
No macroscopic observations noted

* MICROSCOPIC FINDINGS
LIVER
-Deposition pigment: hepatocyte, focal, minimal
SPLEEN
-Extramedullary hematopoiesis, mild
THYMUS
-Lymphoma (non fatal)
Comment: one lobe
SYSTEMIC NEOPLASMS
-Lymphoma malignant, lymphoblastic (malignant tumor)
All other protocol tissues without pathologic findings

ANIMAL NUMBER	:	RDPATH126 SEX: Female -2-010	DOSE GROUP: 2
Defined Sacrifice Group	:	K0	
State at Necropsy	:	Terminal sacrifice	
Date of Necropsy	:	06-May-2021	
Days on Test	:	84 (12-Feb-2021 - 06-May-2021)	

Collection and histopathology evaluation of tissues from wild-type mice treated with Hematopoietic Stem Cells (HSCs) transduced with a Tie2.Ifn2.Ifntiviral plasmid.

R&D PATH 126. Pathology Individual Animal Data

Continued Animal: RDPATH126-2-010

* MACROSCOPIC OBSERVATIONS (Evaluation Incomplete)
No macroscopic observations noted

* MICROSCOPIC FINDINGS
SPLEEN
-Extramedullary hematopoiesis, mild
All other protocol tissues without pathologic findings

ANIMAL NUMBER	:	RDPATH126 -2-011 SEX: Female	DOSE GROUP: 2
Defined Sacrifice Group	:	K0	
State at Necropsy	:	Terminal sacrifice	
Date of Necropsy	:	06-May-2021	
Days on Test	:	84 (12-Feb-2021 - 06-May-2021)	

* MACROSCOPIC OBSERVATIONS
No macroscopic observations noted

* MICROSCOPIC FINDINGS
SPLEEN
-Extramedullary hematopoiesis, minimal
All other protocol tissues without pathologic findings

ANIMAL NUMBER	:	RDPATH126 -2-012 SEX: Female	DOSE GROUP: 2
Defined Sacrifice Group	:	K0	
State at Necropsy	:	Terminal sacrifice	
Date of Necropsy	:	06-May-2021	
Days on Test	:	84 (12-Feb-2021 - 06-May-2021)	

* MACROSCOPIC OBSERVATIONS
No macroscopic observations noted

* MICROSCOPIC FINDINGS
SPLEEN
-Extramedullary hematopoiesis, minimal
All other protocol tissues without pathologic findings
