# Supplementary material for: Myeloid cell‐based delivery of IFN‐γ reprograms the leukemia microenvironment and induces anti‐tumoral immune responses
Source: EMBO Mol Med. 2021 Aug 30;13(10):e13598. doi: 10.15252/emmm.202013598 (PMC8495462; doi:10.15252/emmm.202013598)
Supplement: Supplementary file 2 — Expanded View Figures PDF [file EMMM-13-e13598-s005.pdf]

Expanded View Figures

Figure EV1. IFN-γ and TNF-α gene therapy: engraftment and safety.

A Third-generation SIN lentiviral vector expression cassette driven by a Tie2 enhancer/promoter (Tie2E, Tie2) and post-transcriptionally regulated by two couples of target sites for microRNA-126 and -130a (miR-126T, miR-130aT).

B Experimental design of genetically-modified HSPC transplants followed by challenge with B-ALL.

C Engraftment of CD45.1 donor cells and lineage composition in the blood, as assessed by flow cytometry (mean ± SD, each dot represents an individual mouse, two experiments, CTRL = 12 mice, IFN-γ = 14 mice, TNF-α = 7 mice).

D Vector copy number (VCN) in peripheral blood at 8 weeks post-transplantation (mean ± SD, each dot represents a pool of 2–3 mice, two experiments).

E From left to right: white blood cell count (WBC), red blood cell count (RBC), hemoglobin concentration (HGB), and platelet count (PLT) measured by hemocytometer (mean ± SD, each dot represents an individual mouse, two experiments, CTRL = 12 mice, IFN-γ = 14 mice, TNF-α = 7 mice).

F–I Mice were transplanted with Tie2.NGFR- (CTRL) (n = 6) or Tie2.IFN-γ- (IFN-γ) (n = 6) transduced Lin- cells and evaluated for potential IFN-γ-related toxicity. (F) Serum biochemistry at 8 and 12 weeks after transplantation (mean ± SD, each dot represents an individual mouse). (G) Gene expression levels of *Ifnγ* and IFN-γ-related genes in several organs at steady state at 12 weeks after transplantation (mean ± SD; *P = 0.0182 for BM, *P = 0.0129 for lung, **P = 0.0044, ****$P \leq 0.0001$, ordinary two-way ANOVA) The gray bar shows a single mouse that was systemically (i.p., intraperitoneally) injected with recombinant murine IFN-γ. (H) IFN-γ protein quantification (ELISA) in the plasma of transplanted mice (each dot represents an individual mouse). (I) Bar graphs showing the frequency of HSPC subpopulations in the BM. LSK ($CD45^+$ Lin^- $Sca1^+$ $CD117^+$ $CD34^-$ $CD150^+$), MPP ($CD45^+$ lin^- $Sca1^+$ $CD117^+$ $CD34^+$), MEP/CMP ($CD45^+$ lin^- $Sca1^-$ $CD117^+$); mean ± SD, each dot represents an individual mouse.

© 2021 The Authors

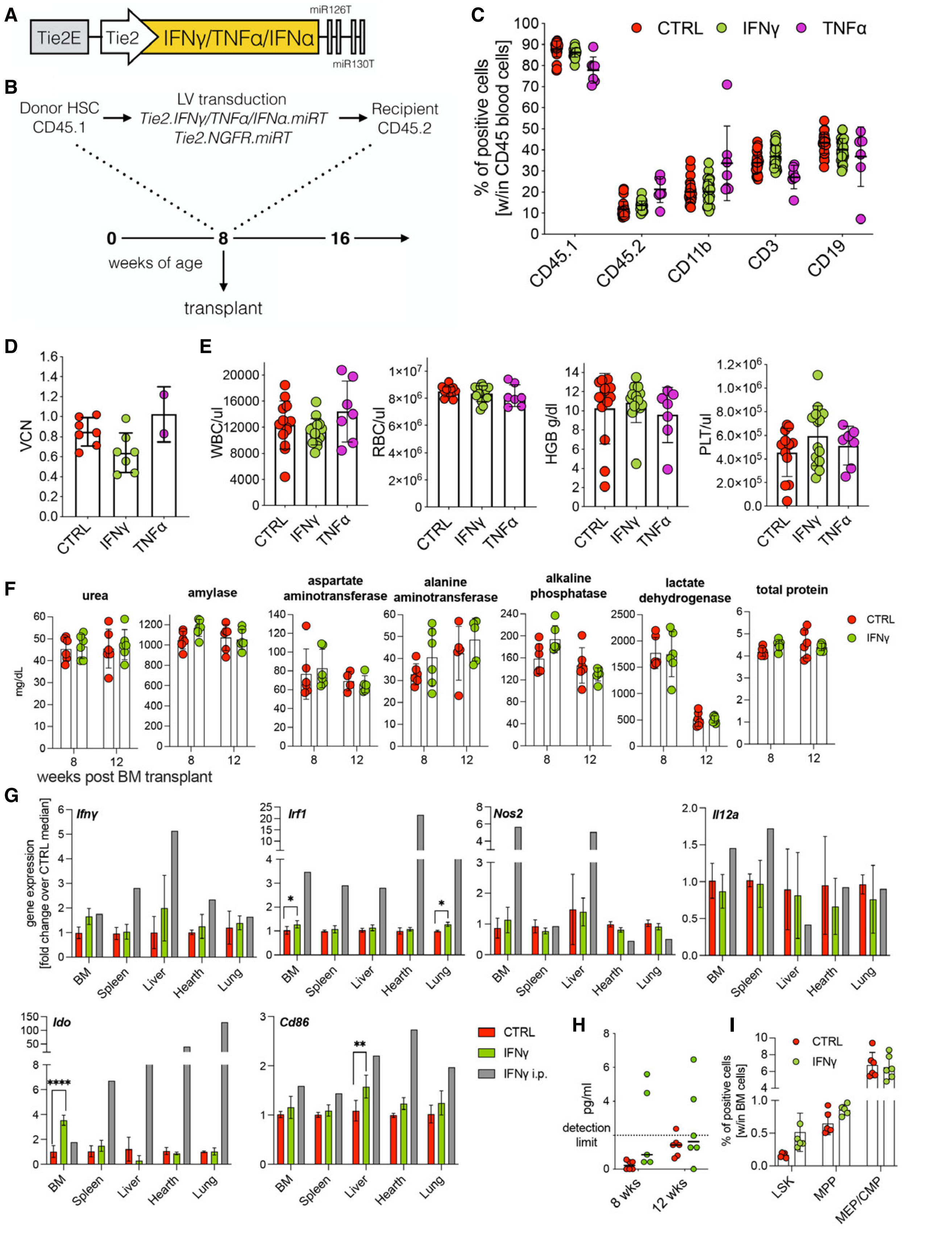
A
Tie2E
Tie2
IFNγ/TNFα/IFNα
miR126T
miR130T
B
Donor HSC
CD45.1
LV transduction
Tie2.IFNγ/TNFα/IFNα.miRT
Tie2.NGFR.miRT
Recipient
CD45.2
0
8
16
weeks of age
transplant
C
CTRL
IFNγ
TNFα
% of positive cells
[w/in CD45 blood cells]
CD45.1
CD45.2
CD11b
CD3
CD19
D
VCN
E
WBC/ul
RBC/ul
HGB g/dl
PLT/ul
F
urea
amylase
aspartate aminotransferase
alanine aminotransferase
alkaline phosphatase
lactate dehydrogenase
total protein
mg/dL
weeks post BM transplant
G
gene expression
[fold change over CTRL median]
Ifnγ
Irf1
Nos2
Il12a
Ido
Cd86
BM
Spleen
Liver
Hearth
Lung
IFNγ i.p.
H
pg/ml
detection limit
8 wks
12 wks
I
% of positive cells
[w/in BM cells]
LSK
MPP
MEP/CMP

Figure EV1.

Figure EV2. IFN-γ, but not TNF-α shows antitumor efficacy in miR-126-driven B-ALL models.

A–C Mice were transplanted with Tie2.NGFR- (CTRL, *n* = 8 mice), Tie2.IFN-γ- (IFN-γ, *n* = 9 mice) or Tie2.TNF-α- (TNF-α, *n* = 7 mice) transduced Lin^- cells and challenged with B-ALL (line#11). (A) B-ALL progression (line#11) measured as absolute number of OFP^+ cells in the peripheral blood of transplanted mice (mean ± SD, each dot represents an individual mouse; $^{**}P = 0.0027$ CTRL vs. IFN-γ and $^{**}P = 0.0063$ CTRL vs. TNF-α, $^{***}P = 0.0003$, two-way ANOVA). (B) Percentage of $CD8^+$ T lymphocytes within OFP^- $CD45^+$ splenic cells (mean ± SD, each dot represents an individual mouse). (C) Percentage of MHC class II-positive macrophages (Mφ), identified by F4/80 expression, in the spleen (mean ± SD, each dot represents an individual mouse; $^{**}P = 0.0063$, ordinary one-way ANOVA).

D–J Mice were transplanted with Tie2.NGFR- (CTRL, *n* = 7 mice) or Tie2.IFN-γ- (IFN-γ, *n* = 16 mice) transduced Lin^- cells, and challenged with an independently generated B-ALL clone (line#8). (D) B-ALL progression (line#8) in peripheral blood measured as the percentage of $CD45.2^{low}$ OFP^+ cells within $CD45^+$ cells (mean ± SD, each dot represents an individual mouse; $^{****}P \leq 0.0001$, ordinary two-way ANOVA with Geisser–Greenhouse correction). (E) B-ALL burden in the BM measured as the percentage of $CD45.2^{low}$ OFP^+ cells within $CD45^+$ cells (mean ± SD, each dot represents an individual mouse). (F) Bar graphs showing MFI of MHC II on OFP^+ B-ALL cells in the BM (mean ± SD, each dot represents an individual mouse, $P = 0.06$, Mann–Whitney test). (G) Bar graphs showing the mean fluorescence intensity of MHC II on BM macrophages (identified as $CD45.1^+$ OFP^- $CD11b^+$ $F4/80^+$ cells) (mean ± SD, each dot represents an individual mouse; $^{**}P \leq 0.01$, Mann–Whitney test). (H) Bar graphs showing the percentage of macrophages within OFP^- cells in the BM (mean ± SD, each dot represents an individual mouse; no significant differences were revealed by Mann–Whitney test). (I) Bar graphs showing the percentage of $CD4^+$ or $CD8^+$ T lymphocytes within OFP^- $CD3^+$ cells in the BM (mean ± SD, each dot represents an individual mouse; no significant differences were revealed by Mann–Whitney test). (J) Distribution of lymphocyte maturation stages within OFP^- $CD8^+$ T cells in the BM (mean ± SD, each dot represents an individual mouse; no significant differences were revealed by Mann–Whitney test).

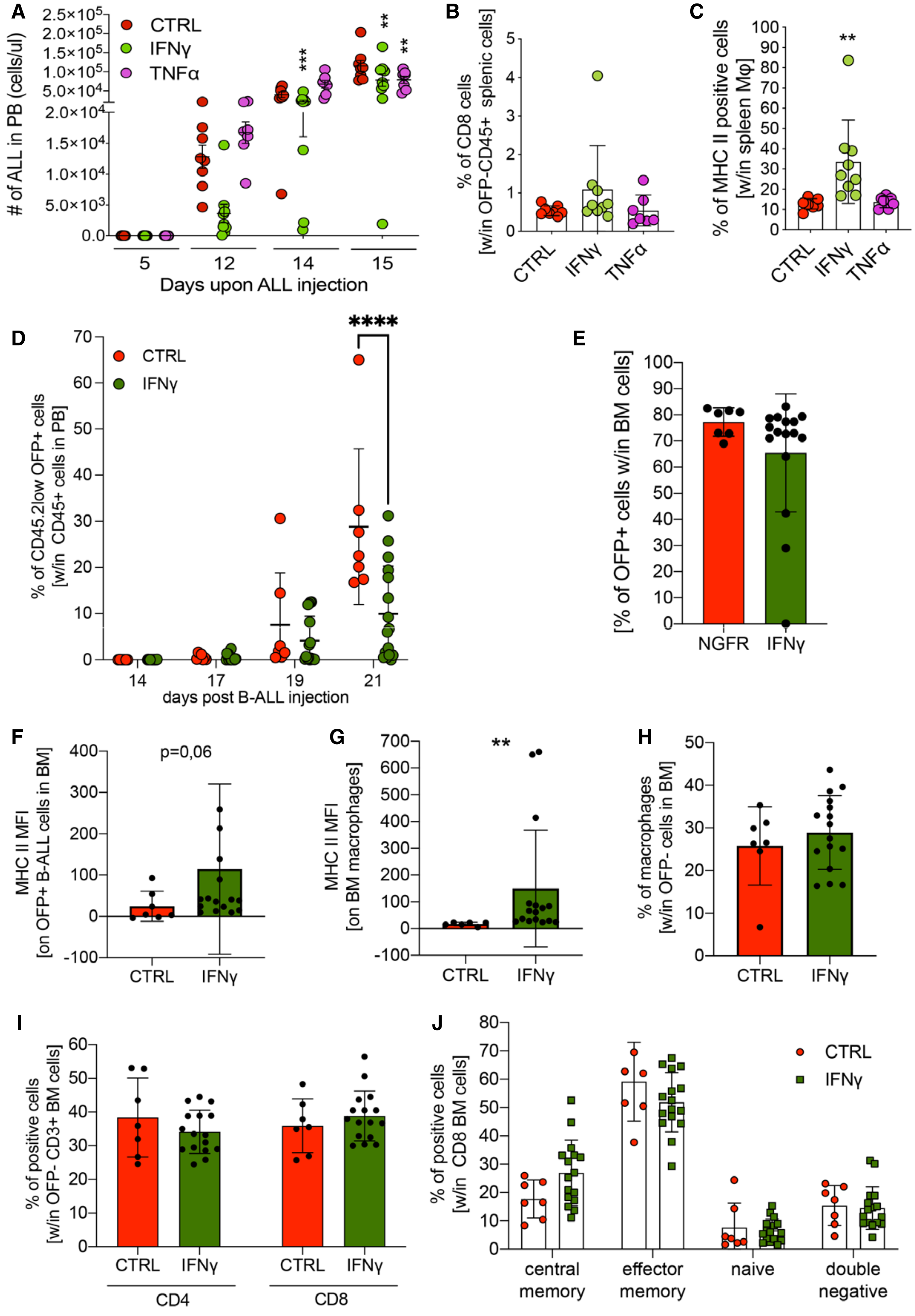

Figure EV2.

 © 2021 The Authors

Figure EV3. IFN-γ gene therapy exerts anti-leukemia effects in a relapse B-ALL model and can be implemented in a humanized setting.

A Experimental design. First part (therapeutic model): female CD45.2 C57 mice were injected with B-ALL (line#11). After 3 days, mice were treated with a single dose of 0.5 mg/kg vincristine. On day 4, mice were lethally irradiated, followed by transplantation of Tie2-IFN-γ-transduced or untransduced CD45.1 lin^- cells the day after. Leukemic burden was monitored by serial blood draws. Second part (relapse model): mice that were tumor-free 8 weeks (day-7) after initial B-ALL injection (confirmed by sentinel BM aspirate) were rechallenged with 1×10^5 B-ALL cells from an $NGFR^+/OVA^+$ subclone of disease#11 (OVA B-ALL) and followed for leukemia growth over time. The experimental endpoint was defined as the first demonstration of leukemia burden above 70% in the blood, or clinical signs of suffering.

B Leukemia burden in the therapeutic B-ALL model (merge of two replicate experiments: Exp#1, IFN-γ = 6 mice, CTRL = 5 mice; Exp#2, IFN-γ = 10 mice, CTRL = 10 mice). Absolute counts of $CD45.2^{low}CD19^+$ blasts in the blood are shown. Note that, at the 12 and 15 week time-points, there was a trend toward lower disease burden in the IFN-γ group (mean ± SD, each dot represents an individual mouse).

C–E Leukemia-free mice from Exp#2 were injected with OVA B-ALL (IFN-γ = 10 mice, UT = 10 mice). (C) Weight loss over time after OVA B-ALL injection (mean ± SD, each dot represents an individual mouse; $^{***}P \leq 0.001$, $^{****}P \leq 0.0001$, two-way ANOVA). (D) Leukemia burden in the relapse B-ALL model (mean ± SD, each dot represents an individual mouse; $^{***}P \leq 0.001$, $^{****}P \leq 0.0001$, two-way ANOVA). (E) Survival curve after OVA B-ALL injection (one experiment, UT = 9 mice, IFN-γ = 9 mice, one experiment, $^{*}P \leq 0.05$, log-rank [Mantel–Cox] test).

F Human peripheral blood mononuclear cells were obtained from buffy coats (*n* = 3 donors), transduced or not with the human TIE2.IFN-γ or a PGK.NGFR control construct in the presence of VPX, and polarized to an M2-like phenotype in culture. As a positive control, soluble IFN-γ protein (sIFN-γ) was used. The levels of human IFN-γ were quantified by ELISA (mean ± SD).

G Human $CD34^+$ cells (single donor) were transduced or not with the human TIE2.IFN-γ construct (vector copy number: 0.81) and cultured for 2 weeks in myeloid differentiating conditions at appropriate cell densities. The growth curve indicates no negative impact from transduction with the TIE2.IFN-γ construct.

H Human $CD34^+$ cells (single donor) were transduced or not with the human TIE2.IFN-γ construct (vector copy number: 0.81, as per point G) and cultured for 2 weeks in methylcellulose. Clonogenic potential (number of CFU-G/M or BFU-E per 3-cm plate) is shown, indicating no negative impact from transduction with the TIE2.IFN-γ construct (three technical replicates, mean ± SEM).

I Schematic representation of a therapeutic, humanized B-ALL model. NSGW41 mice were injected with a luciferase-marked, primary human B-ALL (week 6). After detection of B-ALL by bioluminescence imaging (week 10), two cycles of vincristine/dexamethasone chemotherapy were administered as induction treatment. Mice were then transplanted with two doses of 1×10^6 $CD34^+$ HSPC, transduced or not with the human TIE2.IFN-γ lentiviral vector (weeks 12 and 15). Disease progression was measured on week 17 (2 and 5 weeks after $CD34^+$ cell transplants, respectively). Not surprisingly, there were no differences between experimental groups, as relevant $CD34^+$ HSPC engraftment levels may not be reached until 6 weeks after transplant. To contain B-ALL growth, a single dose of 1×10^6 CD19 CAR-T cells (autologous to the $CD34^+$ graft) was administered (week 18- day 0), and mice were followed by periodic bleeding and bioluminescence imaging until they developed CAR-T-related complications (after day+14).

J Human myeloid cell engraftment in the blood before (day-7) and after CD19 CAR-T cell injection (day+14), estimated as the fraction of human $CD45^+/NGFR^-/CD19^-$ cells in the blood. Note that both the CD19 CAR-T cells and the B-ALL are marked by NGFR. There were no differences between human myeloid engraftment of mock-transduced, TIE2.IFN-γ-transduced or non-CAR-T-treated mice. Engraftment increased to therapeutically relevant levels at day+14 (mean ± SD, each dot represents an individual mouse; h-IFN-γ/CAR-T = 5 mice, UT/CAR-T = 5 mice, w/o CAR-T = 6 mice, three of which from the UT and three from the h-IFN-γ group).

K Monitoring of B-ALL burden by bioluminescence imaging (mean ± SEM; h-IFN-γ/CAR-T = 5 mice, UT/CAR-T = 5 mice, w/o CAR-T = 6 mice, three of which from the UT and three from the h-IFN-γ group). Please note a trend toward lower disease burden on day+14 in the CD19 CAR-T cell-treated mice from the TIE2.IFN-γ group, providing an initial proof of concept that this treatment may be active in a clinically relevant, human leukemia model. Future studies will employ T cell preparations that do not cause xenogeneic graft-versus-host disease, thereby allowing longer follow-up.

L Representative FACS plots (gated on $hCD45^+NGFR^-$ cells) of mice transplanted with TIE2.IFN-γ-transduced $CD34^+$ cells and treated or not with CD19 CAR-T cells on day+14. Note that CD19 CAR-T cell treatment induced human B-cell aplasia, as expected.

© 2021 The Authors

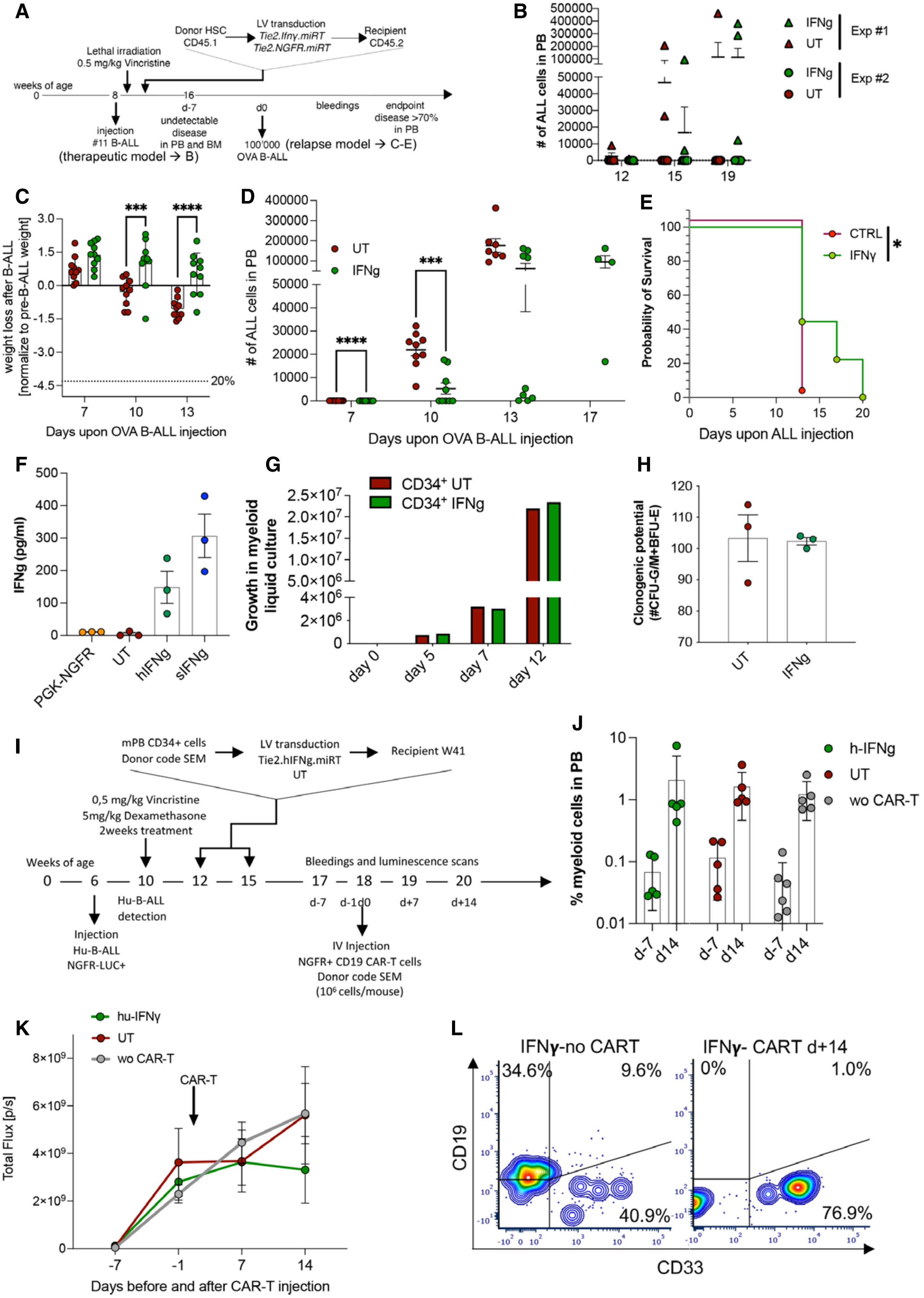

Figure EV3.

© 2021 The Authors

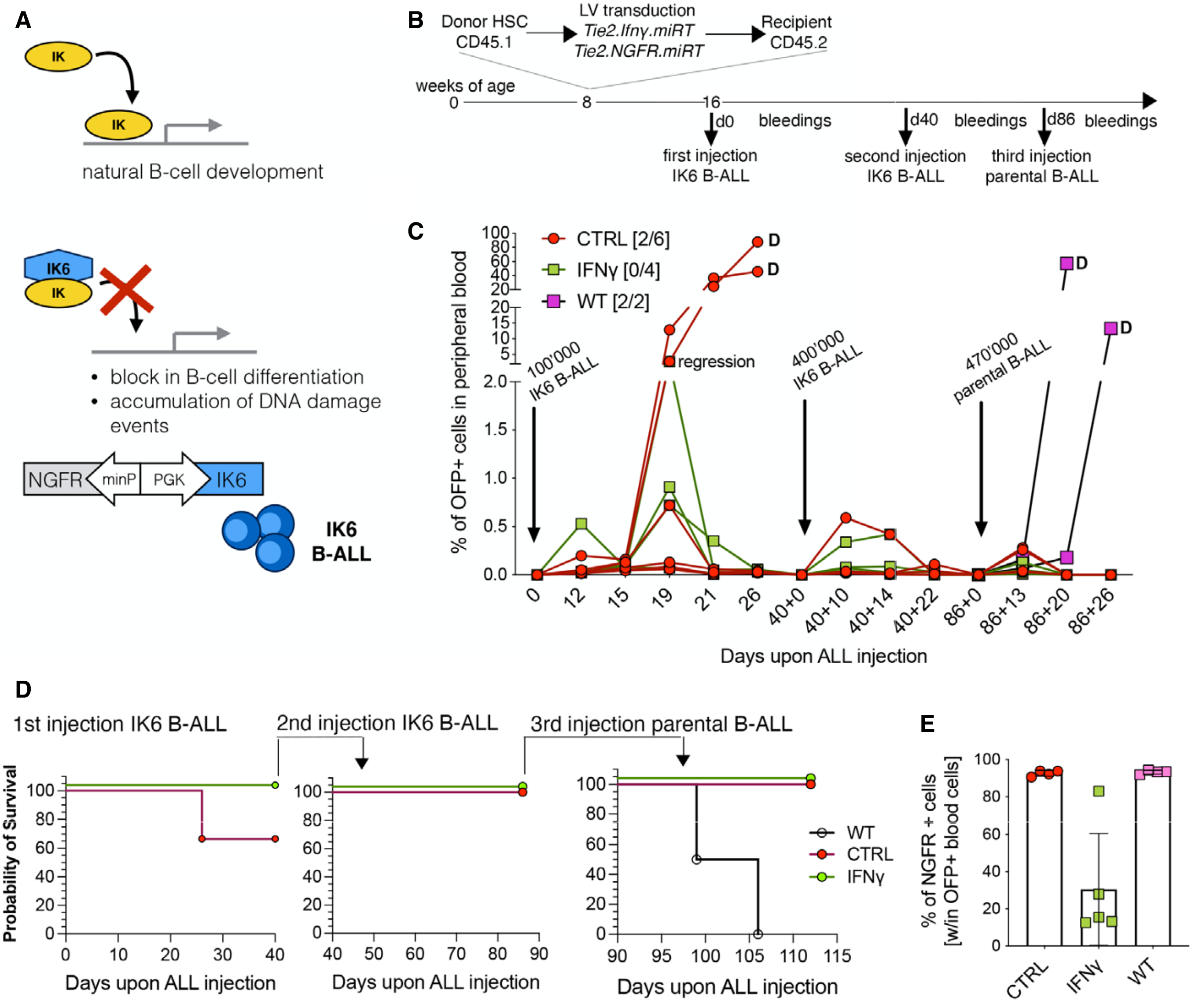

Figure EV4. IFN-γ gene delivery induces antigen loss in IKAROS6 expressing B-ALL.

A Schematic representation of IKAROS6 activity in leukemia, and genetic modification of the parental B-ALL to induce constitutive expression of the dominant-negative IK6 protein.

B Schematic representation of the experimental outline.

C B-ALL progression in the peripheral blood, measured as the percentage of OFP^+ cells within total white blood cells. CTRL, mice engrafted with HSPCs transduced with the Tie2e/p.NGFR vector; IFN-γ, mice engrafted with HSPCs transduced with the Tie2e/p.IFN-γ vector; WT CTRL, sub-lethally irradiated mice that did not receive prior BM transplantation. The numbers in [brackets] indicate the fraction of mice (over the total number) that developed disease upon primary challenge; differences were not significant by Fisher's exact test. Arrows indicate successive disease challenges, with the indicated B-ALL subline.

D Survival curves of three re-challenges with IK6 B-ALL (challenges #1 and #2) and parental line #8 disease (challenge #3; one experiment, CTRL = 6 mice, IFN-γ = 4 mice, WT = 2 mice).

E Expression of NGFR on OFP^+ leukemic cells after the first challenge with IK6 B-ALL (mice from (C), mean ± SD).

Figure EV5. Identification of Tie2-expressing monocytes and non-classical monocytes by scRNAseq analysis.

A UMAP highlighting cells expressing the signature of TEMs, based on the transcriptional signature published by Pucci *et al* (2009).
B Heatmap showing the expression of the genes from the TEM signature within the different myeloid populations defined by our custom signature (see Fig 3C).
C Mapping of the myeloid cell signatures identified by Witkowski *et al* (2020) onto the myeloid compartment of our leukemic mice. Note that the pro-leukemic mHB-M2 cluster colocalizes with our subset of non-classical monocytes.
D Representation of the pro-leukemic mHB-M2 cluster across the different experimental conditions. Note that this cluster becomes more represented in the IFN-γ group on day 17.
E Differential expression of mHB-M2 marker genes in the myeloid cells of our treated animals, according to experimental group.

© 2021 The Authors

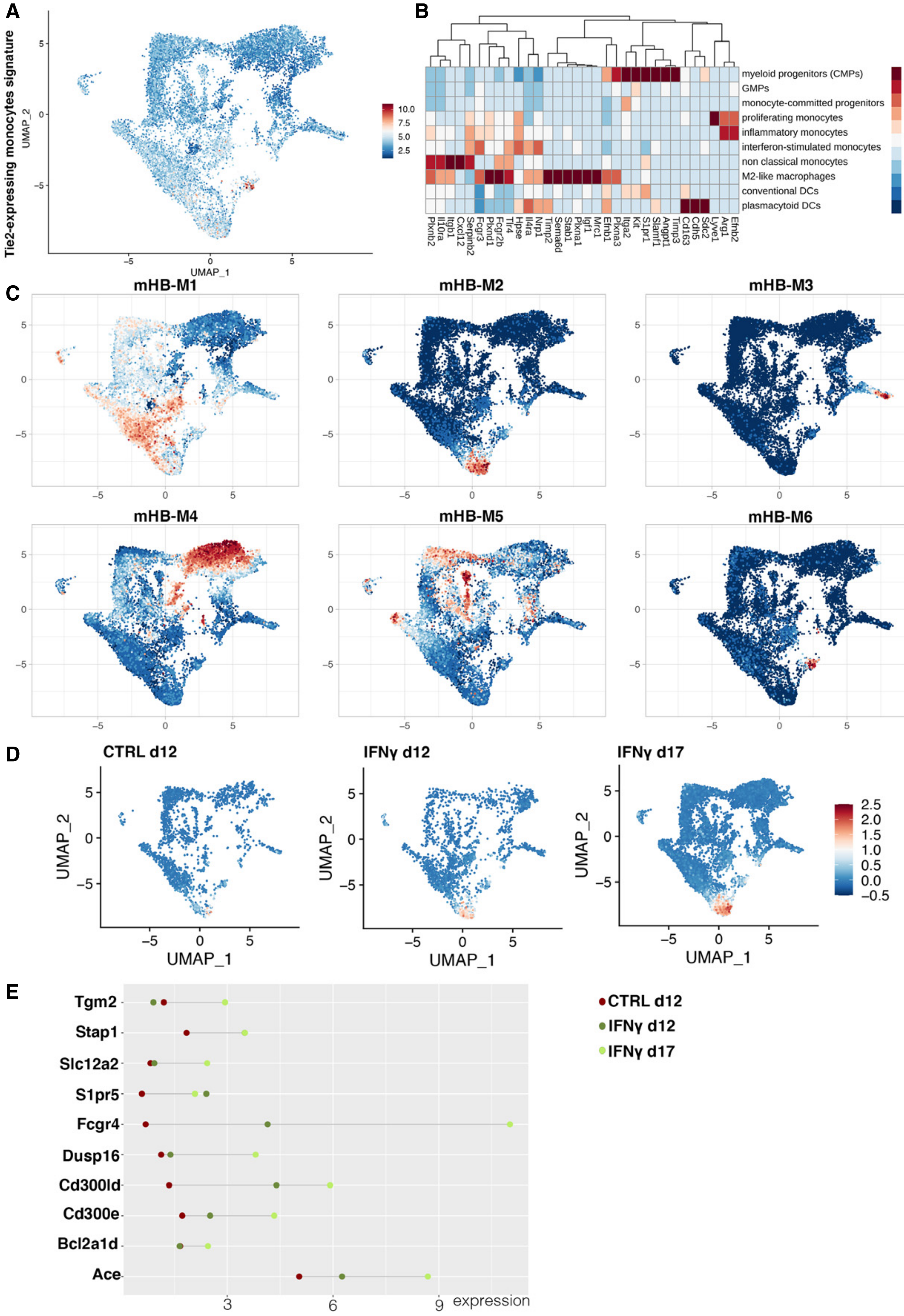
A
Tie2-expressing monocytes signature
UMAP_2
UMAP_1
10.0
7.5
5.0
2.5
B
myeloid progenitors (CMPs)
GMPs
monocyte-committed progenitors
proliferating monocytes
inflammatory monocytes
interferon-stimulated monocytes
non classical monocytes
M2-like macrophages
conventional DCs
plasmacytoid DCs
Plxnb2
Il10ra
Itgb1
Cxcl12
Serpinb2
Fcgr3
Plxnd1
Fcgr2b
Tlr4
Hpse
Il4ra
Nrp1
Timp2
Sema6d
Stab1
Plxna1
Igf1
Mrc1
Efnb1
Plxna3
Itga2
Kit
S1pr1
Slamf1
Angpt1
Timp3
Cd163
Cdh5
Sdc2
Lyve1
Arg1
Efnb2
C
mHB-M1
mHB-M2
mHB-M3
mHB-M4
mHB-M5
mHB-M6
D
CTRL d12
IFNγ d12
IFNγ d17
UMAP_2
UMAP_1
E
Tgm2
Stap1
Slc12a2
S1pr5
Fcgr4
Dusp16
Cd300ld
Cd300e
Bcl2a1d
Ace
expression
CTRL d12
IFNγ d12
IFNγ d17

Figure EV5.
