# Supplementary material for: Myeloid cell‐based delivery of IFN‐γ reprograms the leukemia microenvironment and induces anti‐tumoral immune responses
Source: EMBO Mol Med. 2021 Aug 30;13(10):e13598. doi: 10.15252/emmm.202013598 (PMC8495462; doi:10.15252/emmm.202013598)
Supplement: Supplementary file 8 [file EMMM-13-e13598-s007.pdf]

EMBO Molecular Medicine

Myeloid cell-based delivery of IFN-γ reprograms the leukemia microenvironment and induces anti-tumoral immune responses

Adele Mucci, Gabriele Antonarelli, Carolina Caserta, Francesco Maria Vittoria, Giacomo Desantis, Riccardo Pagani, Beatrice Greco, Monica Casucci, Giulia Escobar, Laura Passerini, Nico Lachmann, Francesca Sanvito, Matteo Barcella, Ivan Merelli, Luigi Naldini, and Bernhard Gentner
DOI: 10.15252/emmm.202013598

Corresponding author: Bernhard Gentner (gentner.Bernhard@hsr.it)

Review Timeline:

Submission Date:	16th Oct 20
Editorial Decision:	16th Nov 20
Revision Received:	11th Jul 21
Editorial Decision:	5th Aug 21
Revision Received:	11th Aug 21
Accepted:	12th Aug 21

Editor: Lise Roth

Transaction Report:

(Note: With the exception of the correction of typographical or spelling errors that could be a source of ambiguity, letters and reports are not edited. Depending on transfer agreements, referee reports obtained elsewhere may or may not be included in this compilation. Referee reports are anonymous unless the Referee chooses to sign their reports.)

16th Nov 2020

Dear Dr. Gentner,

Thank you for the submission of your manuscript to EMBO Molecular Medicine. We have now heard back from the referees who agreed to evaluate your manuscript.
As you will see, while the referees mention the interest of the study, they also raise substantial concerns on your work regarding the strength of the translational aspect, the under-studied mechanistic insights and the presentation of the data, which should be convincingly addressed in a major revision of the present manuscript.

Addressing the reviewers' concerns in full will be necessary for further considering the manuscript in our journal. As revising the manuscript according to the referees' recommendations appears to require a lot of additional work and experimentation, and given the potential interest of your findings, we are ready to extend the deadline to 6 months with the understanding that acceptance of the manuscript would entail a second round of review.
EMBO Molecular Medicine encourages a single round of revision only and therefore, acceptance or rejection of the manuscript will depend on the completeness of your responses included in the next, final version of the manuscript. For this reason, and to save you from any frustrations in the end, I would strongly advise against returning an incomplete revision. Should you find that the requested revisions are not feasible within the constraints outlined here and prefer, therefore, to submit your paper elsewhere, we would welcome a message to this effect.

When submitting your revised manuscript, please carefully review the instructions that follow below. Failure to include requested items will delay the evaluation of your revision:

1) A .docx formatted version of the manuscript text (including legends for main figures, EV figures and tables). Please make sure that the changes are highlighted to be clearly visible.

2) Individual production quality figure files as .eps, .tif, .jpg (one file per figure).

3) A .docx formatted letter INCLUDING the reviewers' reports and your detailed point-by-point responses to their comments. As part of the EMBO Press transparent editorial process, the point-by-point response is part of the Review Process File (RPF), which will be published alongside your paper.

4) A complete author checklist, which you can download from our author guidelines (https://www.embopress.org/page/journal/17574684/authorguide#submissionofrevisions). Please insert information in the checklist that is also reflected in the manuscript. The completed author checklist will also be part of the RPF.

5) Please note that all corresponding authors are required to supply an ORCID ID for their name upon submission of a revised manuscript.

6) Before submitting your revision, primary datasets produced in this study need to be deposited in

an appropriate public database (see
https://www.embopress.org/page/journal/17574684/authorguide#dataavailability).
Please remember to provide a reviewer password if the datasets are not yet public.
The accession numbers and database should be listed in a formal "Data Availability " section (placed after Materials & Method). Please note that the Data Availability Section is restricted to new primary data that are part of this study.

*** Note - All links should resolve to a page where the data can be accessed. ***

7) We would also encourage you to include the source data for figure panels that show essential data. Numerical data should be provided as individual .xls or .csv files (including a tab describing the data). For blots or microscopy, uncropped images should be submitted (using a zip archive if multiple images need to be supplied for one panel). Additional information on source data and instruction on how to label the files are available at
.

8) Our journal encourages inclusion of *data citations in the reference list* to directly cite datasets that were re-used and obtained from public databases. Data citations in the article text are distinct from normal bibliographical citations and should directly link to the database records from which the data can be accessed. In the main text, data citations are formatted as follows: "Data ref: Smith et al, 2001" or "Data ref: NCBI Sequence Read Archive PRJNA342805, 2017". In the Reference list, data citations must be labeled with "[DATASET]". A data reference must provide the database name, accession number/identifiers and a resolvable link to the landing page from which the data can be accessed at the end of the reference. Further instructions are available at .

9) We replaced Supplementary Information with Expanded View (EV) Figures and Tables that are collapsible/expandable online. A maximum of 5 EV Figures can be typeset. EV Figures should be cited as 'Figure EV1, Figure EV2" etc... in the text and their respective legends should be included in the main text after the legends of regular figures.

- For the figures that you do NOT wish to display as Expanded View figures, they should be bundled together with their legends in a single PDF file called *Appendix*, which should start with a short Table of Content. Appendix figures should be referred to in the main text as: "Appendix Figure S1, Appendix Figure S2" etc.

- Additional Tables/Datasets should be labeled and referred to as Table EV1, Dataset EV1, etc. Legends have to be provided in a separate tab in case of .xls files. Alternatively, the legend can be supplied as a separate text file (README) and zipped together with the Table/Dataset file.
See detailed instructions here:
.

10) The paper explained: EMBO Molecular Medicine articles are accompanied by a summary of the articles to emphasize the major findings in the paper and their medical implications for the non-specialist reader. Please provide a draft summary of your article highlighting
- the medical issue you are addressing,
- the results obtained and
- their clinical impact.

This may be edited to ensure that readers understand the significance and context of the research. Please refer to any of our published articles for an example.

11) For more information: There is space at the end of each article to list relevant web links for further consultation by our readers. Could you identify some relevant ones and provide such information as well? Some examples are patient associations, relevant databases, OMIM/proteins/genes links, author's websites, etc...

12) Every published paper now includes a 'Synopsis' to further enhance discoverability. Synopses are displayed on the journal webpage and are freely accessible to all readers. They include a short stand first (maximum of 300 characters, including space) as well as 2-5 one-sentences bullet points that summarizes the paper. Please write the bullet points to summarize the key NEW findings. They should be designed to be complementary to the abstract - i.e. not repeat the same text. We encourage inclusion of key acronyms and quantitative information (maximum of 30 words / bullet point). Please use the passive voice. Please attach these in a separate file or send them by email, we will incorporate them accordingly.

Please also suggest a striking image or visual abstract to illustrate your article. If you do please provide a png file 550 px-wide x 400-px high.

13) As part of the EMBO Publications transparent editorial process initiative (see our Editorial at http://embomolmed.embopress.org/content/2/9/329), EMBO Molecular Medicine will publish online a Review Process File (RPF) to accompany accepted manuscripts.

In the event of acceptance, this file will be published in conjunction with your paper and will include the anonymous referee reports, your point-by-point response and all pertinent correspondence relating to the manuscript. Let us know whether you agree with the publication of the RPF and as here, if you want to remove or not any figures from it prior to publication.

Please note that the Authors checklist will be published at the end of the RPF.

EMBO Molecular Medicine has a "scooping protection" policy, whereby similar findings that are published by others during review or revision are not a criterion for rejection. Should you decide to submit a revised version, I do ask that you get in touch after three months if you have not completed it, to update us on the status.

I look forward to receiving your revised manuscript.

Yours sincerely,

Lise Roth

Lise Roth, PhD
Editor
EMBO Molecular Medicine

To submit your manuscript, please follow this link:

Link Not Available

*Additional important information regarding Figures

https://bit.ly/EMBOPressFigurePreparationGuideline

Each figure should be given in a separate file and should have the following resolution:
Graphs 800-1,200 DPI
Photos 400-800 DPI
Colour (only CMYK) 300-400 DPI"

Figures are not edited by the production team. All lettering should be the same size and style; figure panels should be indicated by capital letters (A, B, C etc). Gridlines are not allowed except for log plots.
Figures should be numbered in the order of their appearance in the text with Arabic numerals. Each Figure must have a separate legend and a caption is needed for each panel.

***** Reviewer's comments *****

Referee #1 (Comments on Novelty/Model System for Author):

Some of the effects are incremental and additional experiments need to be performed to strengthen the data and show some translational ability of this therapeutic strategy in patients with B-ALL or other types of cancer.

INFg and TNFa have long been tested in preclinical models of cancer and some in patients. Targeted delivery is crucial to avoid the systemic toxicity caused by these cytokines. It would be useful to see efficacy on tumor-bearing mice, in other words efficacy on mice that already developed tumors in order to replicate the clinical situation and strengthen the medical impact as well as adequacy of this gene delivery platform.

Referee #1 (Remarks for Author):

This study reports a cell mediated gene delivery of INFg cytokine to treat B-cell acute lymphoblastic leukemia (B-ALL) and comparison to another cytokine TNFa gene. The authors used HSPC to selectively express these cytokines in tumour infiltrating monocytes/macrophages. Cytokines can cause systemic toxicity and need to be targeted. The authors irradiated the mice, then transplanted them with HSPC-expressing cytokines, and subsequently challenged them with B-ALL tumor cells. The results show anti-tumor efficacy of HSPC-mediated expression of INFg cytokine.

I have the following points that need to be addressed before this work can be accepted for publication in EMBO Molecular Medicine.

1. Introduction should provide the rationale for selecting INFg among other existing cytokines.

2. Rationale and advantages of using this type delivery system compared to other existing gene delivery platforms.

3. This study shows proof-of-concept only; how can this be translated into cancer patient treatment? Cancer patients are treated after cancer detection. One translatable experiment would be to challenge the mice with B-ALL cells, then treat them with HSPC expressing INFg and the controls. Mice with established colorectal subcutaneous MC38 tumors can also be used to treat tumor-bearing mice with HSPC-expressing INFg.

4. More data on toxicity, such as monitoring the weight of animals for the duration of the experiment, histopathological analysis of the healthy tissues and lactate dehydrogenase (LDH) levels in the serum are missing and would boost the clinical feasibility. In addition to efficacy, safety is another crucial consideration for any new treatment.

5. An experiment to evaluate the duration of INFg expression in HSPC overtime would provide useful information. Biodistribution of these cells could be performed by screening INFg expression in tissues by ELISA, or by using HSPC expressing a reporter gene.

6. Some of the effects are significant but incremental, it would be interesting to have survival data following single and repeated dosing.

Minor points:
- The last sentence in the abstract: "The activity of IFN was further enhanced...." is copied and pasted at the end of the introduction, this sentence should be rephrased when used for a second time.

- In, Fig-1B increase of CD8+ is incremental.

- Fig-1D, not clear to me why we have additional blue and red colours?

- So, Fig-2B means that the control mice surviving the disease developed an immunisation against the tumour cells? How many mice had spontaneous regression of the B-ALL without treatment? Does this happen in cancer patients?

Referee #2 (Comments on Novelty/Model System for Author):

This is a very novel approach towards immunotherapy that will be of interest to readers. Combination therapies,as demonstrated in the manuscript, will be necessary for a more complete /longlasting response in patients. One issue that probably needs to be addressed is the effect of CTLA4 treatment on T regs as this is now thought to be a major effect of such treatment.

Referee #2 (Remarks for Author):

In this manuscript,the authors have developed a new approach towards immunotherapy, i.e. expression of IFNg in the myeloid cell compartment. This is an interesting approach and efficacy, at least partial, is demonstrated. However the following points should be considered

1. does IFNg have any direct effects on the tumor cells, e.g. growth inhibition, induction of PDL-1,

etc?
2. A major role of anti-CTLA4 treatment is thought to be elimination of Tregs. Is this occurring here.
3. Do the myeloid populations express iNOS or IDO themselves, given the strong induction of these genes by IFNg.
4. Likewise is any expression of IL-27 observed, as this could be a survival factor for the T cells and can be induced by IFNg.
5. Any difference in response based on sex?
6. When the tumors regrow, is any resistance to IFNg obderved in the tumors. This has been shown to be one mechanism seen in tumors.
7. Is there any increased expression of Type 1 interferons in response to the IFNg expression in the myeloid cells? If so, this might impact myeloid population biology.
8. I am not sure all of Fig.2 is essential for the paper. The antigen loss is curious but may be outside the scope and main focus of this study.
9. Were circulating levels of IFNg detected?

Referee #3 (Remarks for Author):

On this manuscript, Adele M., et al. propose an IFN-y gene therapy strategy to manipulate the tumor microenvironment (TME) and restore immune anticancer response. Based on previous studies, the authors genetically manipulate HSPCs to generate IFN-y delivery macrophages which partially reverse the immunosuppressive TME in B-ALL and colorectal cancer models. The authors showed that their system promotes an initial inflammatory environment in the BM detrimental for the leukemia progression. They claimed that such an environment enhance antigen presenting against the tumor and cytotoxic activity of the T-cells. T-cell exhaustion might explain the leukemia relapse.

Immune-based approaches have emerged as an effective treatment option to mitigate tumorigenesis. Therefore, the relevance of this study relies on its potential therapeutic impact. The in vivo experiments are well designed and some results are intriguing. However, there are major concerns on the mechanistic model. The propose therapy is only efficient for a short period of time (17 days). Therefore, a deep understanding on the IFN-y-mediated changes in the microenvironment, a comprehensive mapping of the interactions between leukemic and immune cells and the molecular mechanisms driving treatment susceptibility and relapse would enhance the novelty and the relevance of this study. Regardless, the analysis of the scRNA-seq data is very superficial and the results are poorly presented in the Figures. Functional validation of the findings is lacking. Finally, it is unclear which are the benefits of IFN-Y genetic transfer vs systemic treatment. Taking all these into account, I recommend that the authors address the following concerns before considering the manuscript for publication.
Specific Major Points:
1. Authors claimed that the genetic manipulation of HSPCs to generate pro-inflammatory macrophages would be safest than systemic administration:
o Is the efficiency similar? Did the authors compare side by side systemic administration and genetic transfer?
o While manipulation of HSPCs might result in normal hematopoiesis under-steady state conditions, the generation of IFN-y delivery macrophages might be detrimental during inflammatory response triggered by infections or chronic inflammation. Thus, are those mice more susceptible to LPS-mediated endotoxin shock?
o Supp. Fig 1: Authors claimed that the genetically modified HSPCs (to express IFN-y and TNF-a under the control of Tie2e/p) are able to fully reconstitute hematopoiesis with no major

abnormalities (Supp fig. 1C-H). However, the analysis is based in peripheral blood parameters. Quantifications of HSC, MPP and other progenitors in the bone marrow should be included.
2. Figure Sup 3: Do the macrophages and CD8 cells in the colorectal model show increased levels of MHC II?Do the colorectal cells also upregulate MHC II?
3. Figure 2: A scheme explaining the time line of the different challenges will help to understand the experimental approach.
4. Survival curves in Figures 2, and 6 would strength the data.
5. Representative FACS plot should be included for the gating strategy of the cells and for the quantification of the levels of MHC II
6. The scRNA-seq (Figure 4 and 5) is poorly analyzed. Authors should review some of the published studies to proper present their results, particularly the bone marrow landscape of B-ALL recently published by Witkowski M. et al., Cancer Cell 2020.
o Figure 4a, b and c: each cluster should properly correlate with a cell type. I recommend the authors to plot: a) UMAP-color coded for unbiased cluster, b) UMAP-color coded for each condition (Control 12, Control 17, IFN-y 12 and IFNy 17) and c) UMAP-color coded for cell calling.
o A heatmap or a BubbleMap showing the levels of the main genes used as cell markers should accompanied Figure 4a, 4b and 4c.
o Cluster contribution in each condition should be addressed.
o It is unclear whether B-ALL cells are present in Figure 4a.
o Which is the TEM signature include in Figure 4d? A heatmap/BubbleMap should be included
o Which is the distribution of the cell types included in Figure 4c per sample? Are there differences between them?
o What are: interferon stimulated monocytes and inflammatory monocytes? Based on which gene expression patterns the authors are separating these two populations?
o Where are the classical monocytes (which is the major population of the bone marrow) in Figure 4c?
o A subset of Non classical monocytes has been shown to play essential roles in the progression of B-ALL (Witkowski et al., 2020). Can the authors identify these subsets in their study? Do IFN-y mice show less non-classical monocytes at day 12 than day 17? How do the authors correlate their results with this study?
7. Overall, it is unclear the transcriptional and molecular mechanisms by which IFN-y promotes a delay in the leukemia progression at day 12 and how or why leukiemic cells scape. On the one hand the authors claimed that B-ALL cells increase MHCII and suggest that they could become a target from the immune system. However, do the authors observe increased cell death in the RNA-seq or signs of cytotoxic activity? Are there differences in proliferation? On the other hand, the authors claimed that on d17 the B-ALL cells become more oncogenic (upregulation of Myc, Kit…etc). Is the IFN-y signaling directly delaying the oncogenic transformation?
8. Which are the transcriptional differences between B-ALL cells that express high and low MHC II levels?
9. Are there differences in the percentage of exhausted T cells between the different conditions studied included in the scRNA-seq? Can authors correlate data on Figure 4b and Figure 5B and C?
10. Authors should review the writing. Some expressions are inaccurate (f.i. "a subline that models progressed form of leukemia") and many sentences are complicated and difficult to understand.

Minor:
1. The legend of Figure 1 should clarify the differences in the tumor burden on experiments showed on 1a-b and 1c-d.
2. Representative FACS plots and gating strategy should be included, specially to characterize: Macrophages, Monocytes…
3. Figure 2: Survival representation should complement the PB quantification of B-ALL cells.

4. Figure 2: The authors claims that 2/3 control mice died from B-ALL (Figure 2B) but the graphs shows 4 control mice.
5. Peripheral blood numbers of T (CD3+) and B (CD19) cells in Figure 3a doesn't represents normal hematopoiesis. Usually at steady-state hematopoiesis or 4-5 weeks after transplantation the distribution of B-cells is around 60-70% and T-cells below 25%.
6. Statistical analysis should be included in Figure 4e and f.

Referee #1 (Comments on Novelty/Model System for Author):

Some of the effects are incremental and additional experiments need to be performed to strengthen the data and show some translational ability of this therapeutic strategy in patients with B-ALL or other types of cancer.

INFg and TNFa have long been tested in preclinical models of cancer and some in patients. Targeted delivery is crucial to avoid the systemic toxicity caused by these cytokines. It would be useful to see efficacy on tumor-bearing mice, in other words efficacy on mice that already developed tumors in order to replicate the clinical situation and strengthen the medical impact as well as adequacy of this gene delivery platform.

We thank the reviewer for his sharp and constructive comments on our manuscript.

Referee #1 (Remarks for Author):

This study reports a cell mediated gene delivery of INFg cytokine to treat B-cell acute lymphoblastic leukemia (B-ALL) and comparison to another cytokine TNFa gene. The authors used HSPC to selectively express these cytokines in tumour infiltrating monocytes/macrophages. Cytokines can cause systemic toxicity and need to be targeted. The authors irradiated the mice, then transplanted them with HSPC-expressing cytokines, and subsequently challenged them with B-ALL tumor cells. The results show anti-tumor efficacy of HSPC-mediated expression of IFNg cytokine.

I have the following points that need to be addressed before this work can be accepted for publication in EMBO Molecular Medicine.

1. Introduction should provide the rationale for selecting INFg among other existing cytokines.

As recommended by the Reviewer, we have added a sentence to the introduction, further explaining the rationale for selecting IFNg for our immunotherapy approach:
"With type-I interferons, tumor necrosis factor (TNF)-α, and some interleukins (e.g., IL-12), IFNγ is a top cytokine candidate to stimulate antitumor immunity."

2. Rationale and advantages of using this type delivery system compared to other existing gene delivery platforms.
We have added the following sentence to the discussion:
"Advantages of this delivery platform include limited systemic cytokine exposure and the need for a single, one-off treatment only, as genetically modified HSPCs guarantee persistence."

3. This study shows proof-of-concept only; how can this be translated into cancer patient treatment? Cancer patients are treated after cancer detection. One translatable experiment would be to challenge the mice with B-ALL cells, then treat them with HSPC expressing INFg and the controls. Mice with established colorectal subcutaneous MC38 tumors can also be used to treat tumor-bearing mice with HSPC-expressing IFNg.
We thank the reviewer for this relevant question. Similar to autologous or allogeneic transplantation, our gene-based treatment strategy is intended as a consolidation therapy, to deplete residual disease refractory to standard treatment thereby avoiding relapse/metastasis. In practical terms, eligible patients first undergo standard chemotherapy inducing remission and creating a time window of clinically-stable disease, in which the autologous HSPC, endowed with antitumor activity by means of genetic engineering, may be engrafted. We are conducting a clinical study in patients with glioblastoma (NCT03866109), where autologous HSPC transduced with a human IFNα cassette are transplanted after surgical and radiotherapeutic de-bulking of the primary tumor. We have so far treated 15 patients demonstrating clinical feasibility of the approach.
The above-described human scenario is difficult to model in mice, due to the scarcity of relevant leukemia relapse models and optimized intensive chemotherapy regimens applicable to the mouse

setting. We explored the possibility to temporarily control an established B-ALL disease by a combination of vincristine chemotherapy and various doses of total body irradiation that would allow the engraftment of syngeneic HSPC transduced with the Tie2-IFNg cassette. The window between disease relapse and disease eradication was narrow and variable between experiments, requiring careful set up experiments.

The revised version now contains new experiments in a therapeutic murine B-ALL model, whereby leukemia-bearing mice receive chemoradiotherapy, followed by transplantation of Tie2-IFNg-engineered cells confirming significant treatment benefit (New Fig. 1H-I). A relapse model (New EV 4A-E) shows improved clinical condition and significantly longer survival upon IFNg gene therapy. Results are described in the text:

"To approach a more clinically relevant experimental model, we tested the efficacy of IFNγ gene therapy in a therapeutic setting. Mice challenged with line #11 B-ALL received chemoradiotherapy for disease control and conditioning, and were then transplanted with gene-modified lineage-negative HSPCs (Fig. 1H). Importantly, in this therapeutic setting, IFNγ gene therapy resulted in significant leukemia growth inhibition compared to control animals (Fig. 1I). In replicate experiments, where vincristine chemotherapy and irradiation were given earlier after B-ALL injection (EV 4A), most animals were cured from leukemia in both IFNγ and control groups (EV 4B). To model B-ALL relapse, mice surviving the first challenge were then injected with a B-ALL subclone of line #11 (NGFR+/Ovalbumin+). Mice from the IFNγ gene therapy group showed a significant delay in relapse kinetics (EV 4C), which translated into improved clinical condition (EV 4D) and prolonged survival (EV 4E)."

To further support the translatability of cytokine-engineered HSPC transplantation, we constructed a human IFNγ vector and tested its safety both *in vitro* and *in vivo* in a xenograft model. *In vitro* testing of the human IFNγ construct showed IFNγ release in CD34-transduced differentiated myeloid cells, reduced expression of immunosuppressive cytokines such as IL-10 and induction of IFN responsive genes (data not shown). Moreover, transduced cells showed similar growth curve kinetics in liquid culture and comparable colony forming potential to untransduced cells. We also performed a pilot experiment on a highly challenging human xenograft model featuring primary B-ALL transplantation, induction chemotherapy, transplantation of CD34+ cells transduced with the IFNg construct and infusion of autologous CART cells against CD19 (New EV 4F-K). Even though we realize that this data represents an initial proof-of-concept, to be confirmed in follow up studies, we believe there is value in including this experiment, which further underlines the clinical relevance and potential translatability of our work. We have added to the main text:

"To further confirm clinical translatability, a humanized TIE2.IFNγ construct was designed, validated for functionality and absence of toxicity on human culture-derived M2 macrophages and CD34+ hematopoietic stem and progenitor cells, and tested in a therapeutically-relevant model of human B-ALL, in combination with CD19 CAR-T cells (EV 4F-K)."

A detailed description of the experiment is provided in the figure legends accompanying Extended View Figure 4.

4. More data on toxicity, such as monitoring the weight of animals for the duration of the experiment, histopathological analysis of the healthy tissues and lactate dehydrogenase (LDH) levels in the serum are missing and would boost the clinical feasibility. In addition to efficacy, safety is another crucial consideration for any new treatment.

We thank the reviewer for raising this important point. To answer to this issue, we have set up a toxicity study, where mice were transplanted with either control vector- or IFNg vector-transduced cells, and carefully monitored. We checked several biochemical parameters on mice sera (urea, amylase, aspartate aminotransferase, alanine aminotransferase, alkaline phosphatase, lactate dehydrogenase, serum proteins) at two time-points (8- and 12-weeks post-transplantation). As shown in the New EV 1F, no significant differences were detected between IFNg engrafted mice and control mice. There were no intercurrent deaths, no differences in body weight between the 2 groups and no macroscopic abnormalities at necropsy. Organ histopathology was performed by an

expert mouse pathologist, and the full pathology report is attached to the submission. No major alteration was observed, except for one IFNg mouse, which showed a T cell lymphoblastic lymphoma in the thymus (incidental finding). However, as highlighted in point 7 of the report, lymphoma is a spontaneous tumor in mice and is enhanced by irradiation. Therefore, no causal link can be established with the gene therapy treatment. Notably, as part of the preclinical package for IFNa gene therapy, which uses the same Tie2-driven vector backbone, we have performed formal GLP toxicity studies on hundreds of mice. Also that study highlighted the occurrence of thymomas in a fraction of mice, but none was caused by vector insertions.
The text has been updated as follows:
When transplanted into lethally irradiated CD45.2 recipients, these cells, independently from the transgene, fully reconstituted hematopoiesis of the animals, with the persistence of gene-marking *in vivo* with vector copy numbers (VCN) ranging from 0.4 to 1 (EV 1C, D). Conversely, transplantation of cells where these cytokines were expressed from a ubiquitous (PGK) or strong myeloid-specific promoter (SP146-gp91) resulted in 100% lethality by day 14 (data not shown). Instead, no significant hematologic abnormalities were observed compared to controls, except a minor T cell reduction in the TNFα group, suggesting specificity of gene expression control by the miRNA-regulated Tie2e/p cassette (EV 1C, E), confirmed by the modest upregulation of IFNγ-responsive genes in the tissues, without altering blood biochemical parameters and with barely detectable IFNγ levels in the plasma of engrafted mice (EV 1F-H). For IFNγ, an in-depth toxicity study was performed, confirming that transduced cell engraftment was stably maintained without negative impact on BM progenitor cell numbers (EV 1I). Necropsy with organ histopathology did not reveal abnormalities, except for an incidental finding of thymoma in a single mouse (Appendix 1).

5. An experiment to evaluate the duration of INFg expression in HSPC overtime would provide useful information. Biodistribution of these cells could be performed by screening INFg expression in tissues by ELISA, or by using HSPC expressing a reporter gene.
In order to answer to this question, we have measured Ifng-related gene signatures on several organs from the mice of the toxicity study (EV 1G) and performed IFNg measurement in the blood plasma by ELISA (EV 1H). Overall, these analyses revealed a tight regulation of the vector cassette, with cytokine levels barely above the detection limit (2 pg/ml) under steady-state condition, and no widespread activation of an inflammatory gene signatures in several organs. Serial measurements of IFNg levels in the plasma (EV1H) and vector copy number on hematopoietic cells (not shown) showed stability over time, as expected from an HSC graft.

6. Some of the effects are significant but incremental, it would be interesting to have survival data following single and repeated dosing.
As engraftment of genetically-engineered HSPC requires conditioning, and -once engrafted- the genetic engineering remains stable over time, repeated dosing is not contemplated for ex vivo HSPC based gene therapies. We have included a new survival experiment in the manuscript:
"To model B-ALL relapse, mice surviving the first challenge were then injected with a B-ALL subclone of line #11 (NGFR+/Ovalbumin+). Mice from the IFNγ gene therapy group showed a significant delay in relapse kinetics (EV 4C), which translated into improved clinical condition (EV 4D) and prolonged survival (EV 4E)."

Minor points:

- The last sentence in the abstract: "The activity of IFNgwas further enhanced...." is copied and pasted at the end of the introduction, this sentence should be rephrased when used for a second time.

The end of the intro has been rephrased: ”As chronic IFNγ exposure induced counterregulatory responses undermining its efficacy, we have shown that combination therapies improve therapeutic efficacy.”

- In, Fig-1B increase of CD8+ is incremental.
This has been added.

- Fig-1D, not clear to me why we have additional blue and red colours?
We utilized different colors for those mice that were utilized for the single cells RNA sequencing analysis. We have now specified this in the figure caption.

- So, Fig-2B means that the control mice surviving the disease developed an immunisation against the tumour cells? How many mice had spontaneous regression of the B-ALL without treatment? Does this happen in cancer patients?
In general the disease expressing IK6, originating from the disease line #8, is less aggressive (i.e., it carries a lower number of leukemia-initiating cells) compared to the parental line #11 used in the other experiments. Moreover, the introduction of artificial antigens including the human NGFR and the human IKAROS6 may have increased its immunogenicity. Furthermore, even if not proven in our model, IKAROS6 expression in human leukemias is associated with increased mutational burden. Taken together, these factors may explain why also 4 out of 6 CTRL engrafted mice did not develop or regressed from the initial injection of the IK6-disease. However, the fact that all animals that survived the first challenge were resistant to subsequent challenges by the parental leukemia (devoid of the artificial antigens NGFR and IKAROS6) argues for an immune-mediated rejection, with antigen-spreading to other leukemia-associated antigens. In cancer patients, this scenario cannot be fully recapitulated since, for vaccination purposes, one can only challenge with putative cancer antigens but not actual diseases. The IK6 model has been de-emphasized and moved to Supplementary material (New EV5), as suggested by the other reviewer.

Referee #2 (Comments on Novelty/Model System for Author):

This is a very novel approach towards immunotherapy that will be of interest to readers. Combination therapies, as demonstrated in the manuscript, will be necessary for a more complete /longlasting response in patients. One issue that probably needs to be addressed is the effect of CTLA4 treatment on T regs as this is now thought to be a major effect of such treatment.

We thank the reviewer for the positive comments about our work and for pointing out relevant questions, which we tried to address in this revision.

Referee #2 (Remarks for Author):

In this manuscript,the authors have developed a new approach towards immunotherapy, i.e. expression of IFNg in the myeloid cell compartment. This is an interesting approach and efficacy, at least partial, is demonstrated. However, the following points should be considered

1. does IFNg have any direct effects on the tumor cells, e.g. growth inhibition, induction of PDL-1, etc?

As shown in the response to Reviewer 3 point 7, IFNg treated B-ALL has reduced viability (shown by Annexin V staining, New Fig. 5E) as well as reduced proliferation (scRNA seq data for Ki67, New Fig. 5F). Such effects might be caused by a direct effect of IFNg on the responding disease. In the answer to the reviewer 3 point 8, we show that a fraction of leukemic cells responds to IFNg stimulation by up-regulating MHC II (New Fig.5G-H). By comparing MHC II high versus low cells within the IFNg d12 sample, we observed diverse pathway activation. In detail, MHC II high cells upregulate inflammation and type I and II interferon pathways, while the MHC II low cells have

higher activation of pathways associated with proliferation, oncogenic transformation, and metabolic alterations. This MHC II high population that seems to respond to IFNg progressively disappears. However, most of the IFNg anti-tumoral activity seems to be immune mediated and not a direct effect on the disease as proven by the experiments in Figure 2. The text has been modified as follows:

"Similarly, B-ALL showed transcriptional heterogeneity by unsupervised analysis (Fig. 5A), with transient up-regulation of an IFNγ gene signature as well as MHC II molecules (Fig. 5B, C). This was accompanied by a reduction of the potential neoantigen OFP, which is co-expressed with the miR-126 oncogene (Nucera *et al*, 2016) (Fig. 5D). Moreover, B-ALL cells from IFNγ treated animals displayed decreased viability and reduced expression of the proliferation marker Mki67 compared to controls (Fig. 5E, F). Based on the MHC II molecules expression module score, we divided B-ALL cells from IFNγ d12 into an MHCII-high subpopulation, displaying enrichment for IFN-responsive gene modules and an MHCII-low subpopulation, with higher expression of proliferative and oxidative phosphorylation pathways (Fig. 5G, H). Moreover, at day 17, B-ALL cells from treated animals showed reduced expression of the IFNγ receptors and transducers (Ifngr1, Ifngr2, and Jak1), accompanied by reduced intracellular signaling via Stat1 and Irf1 (Fig 5I), possibly indicating the development of a resistance mechanism to IFNγ (Arenas *et al*, 2018)."

2. A major role of anti-CTLA4 treatment is thought to be elimination of Tregs. Is this occurring here.
We thank the reviewer for pointing out this relevant mechanism of anti-CTLA4 treatment. We have studied Tregs in frozen samples from the combination experiment (Fig.6E-G), where we combined our gene therapy approach with anti-CTLA4 antibodies. The proportion of Treg cells within the CD4+ BM T cell compartment was high. However, no significant difference was observed in terms of FOXP3+ CD4 T cells, nor of Treg cells (gated on CD3, CD4 and FOXP3 CD25) in the bone marrow of the animals, as shown in the figures below. Interestingly, the combination treatment of IFNγ gene therapy with anti-CTLA4 significantly reduced expression of PD-1 compared to untreated controls in BM Treg cells. This downregulation might reduce their immunosuppressive capacity (Wang C, Li Y, Proctor TM, Vandenbark AA, Offner H. Down-modulation of programmed death 1 alters regulatory T cells and promotes experimental autoimmune encephalomyelitis. J Neurosci Res. 2010 Jan;88(1):7-15. doi: 10.1002/jnr.22181. PMID: 19642196; PMCID: PMC2783709.)

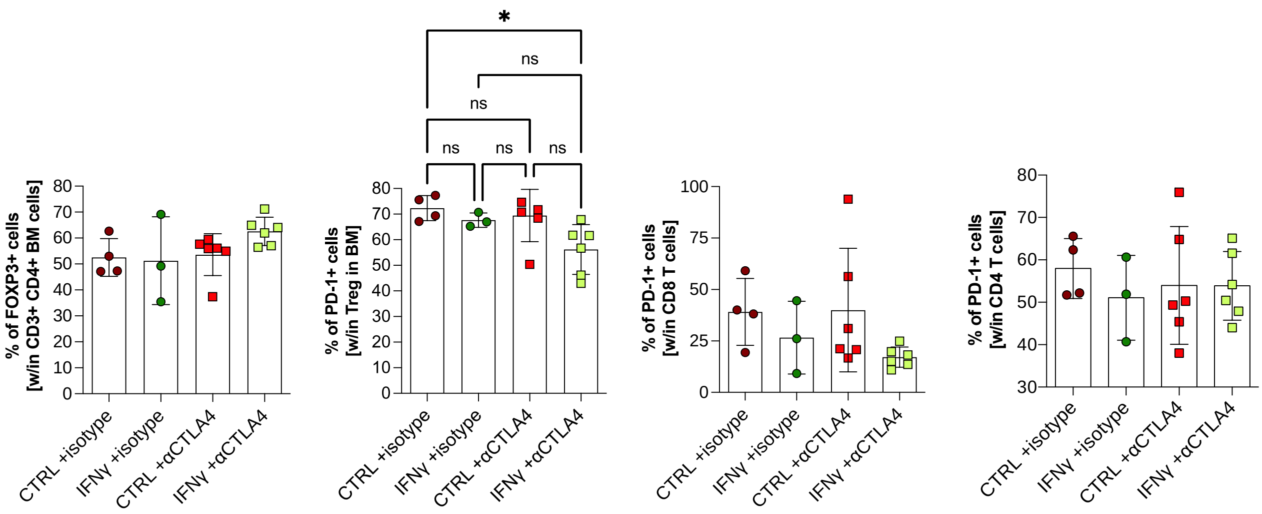

3. Do the myeloid populations express iNOS or IDO themselves, given the strong induction of these genes by IFNg.
We evaluated the expression of iNOS and IDO in single cell RNA sequencing and could not find cells expressing them. This doesn't exclude that such genes are expressed, as the levels might be below the detection limit of the single cell RNA sequencing. Indeed, ddPCR assay evaluating

IFNg-induced genes show that our gene therapy induces expression of some of them in the BM, while only small if any induction was seen in other organs such as heart, lungs, spleen and liver (New EV 1G).

4. Likewise is any expression of IL-27 observed, as this could be a survival factor for the T cells and can be induced by IFNg.
We really appreciate the reviewer's suggestion and looked for IL-27 expression in the myeloid compartment. As shown in the New Fig. 4D, we did observe higher numbers of cells expressing IL-27 in the IFNγ treated animals compared to controls. Interestingly, IL-27 expression colocalizes with the population having the highest signature for IFNγ. The text has been modified as follows:

"Notably, we see widespread induction of IL-27, a T cell pro-survival factor (Schneider *et al*, 2011), in myeloid cells from the IFNγ group, persisting to day 17 (Fig. 4D)."

5. Any difference in response based on sex?
Our experiments were performed in female mice, as the B-ALL we used was generated in female animals. Sex matching may be relevant in the context of immunological studies to avoid unexpected and undesirable non-specific immune activation Therefore, we followed the common practice to match gender for transplantation experiments.

6. When the tumors regrow, is any resistance to IFNg observed in the tumors.

We appreciate the reviewer's suggestion and, based on mechanisms described in literature (Arenas EJ, et al. Acquired cancer cell resistance to T cell bispecific antibodies and CAR T targeting HER2 through JAK2 down-modulation. Nature Communications 2021, Castro F, et al. Interferon-Gamma at the crossroads of tumor immune surveillance or evasion. Frontiers in Immunology 2018), we verified down-regulation and up-regulation of genes described to induce resistance to IFNg in tumors. As shown in the New Fig. 5I, B-ALL cells show reduced expression of receptors and signal transducers of IFNg (Ifngr1, Ifngr2 and Jak1) at the late time point (day 17). This reduction is accompanied by reduced intracellular signaling via Stat1 and Irf1.
The text has been modified as follows:
"Moreover, at day 17, B-ALL cells from treated animals showed reduced expression of the IFNγ receptors and transducers (Ifngr1, Ifngr2, and Jak1), accompanied by reduced intracellular signaling via Stat1 and Irf1 (Fig 5I), possibly indicating the development of a resistance mechanism to IFNγ (Arenas *et al*, 2018)."

7. Is there any increased expression of Type 1 interferons in response to the IFNg expression in the myeloid cells? If so, this might impact myeloid population biology.
As evidenced by type I IFN-stimulated genes analyzed in the myeloid population of our scRNAseq dataset, we see a mild upregulation of type I responses in the presence of IFNg gene therapy (see figure below).

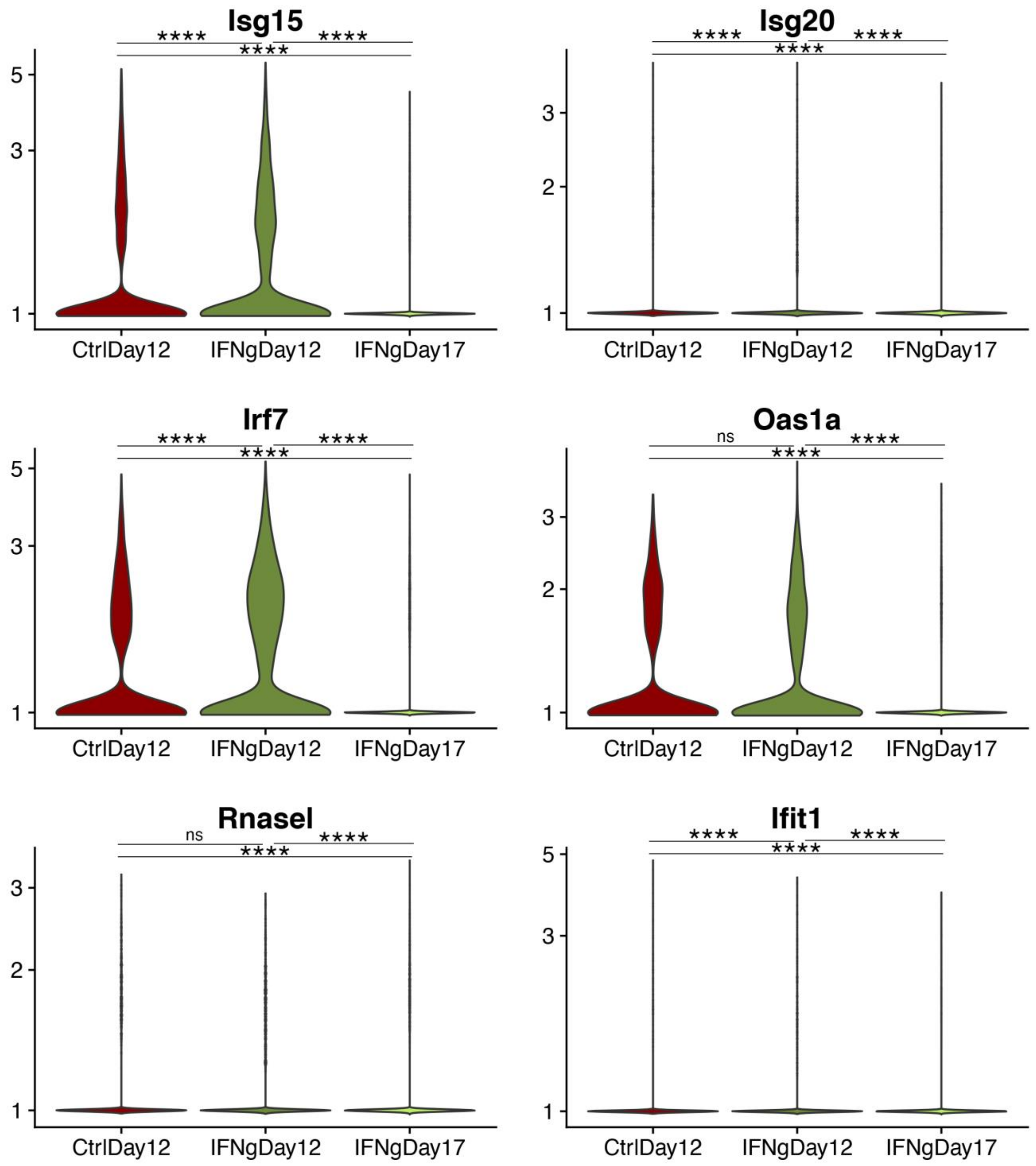

8. I am not sure all of Fig.2 is essential for the paper. The antigen loss is curious but may be outside the scope and main focus of this study.

We appreciated the reviewer comment and agree that former Figure 2 has a different angle compared to the rest of the paper. However, we still think this information is relevant in explaining the mechanisms by which IFNg induces anti-tumoral effects. Even though this figure is not fully focused on the therapeutic potential of our gene therapy approach, we think that antigen loss as well as acquisition of a more aggressive phenotype are the mechanisms that lead to efficacy loss. By showing a disease carrying additional artificial antigens that are specifically lost in the treated animals, we confirm that the disease we are using as a model is especially aggressive and that IFNg is causing a stronger immune pressure that leads to activation of immune responses and eventually in antigen escape. The data has now been moved to Supplementary information (New Fig. EV5).

9. Were circulating levels of IFNg detected?

We performed serial IFNg measurement in the peripheral blood of transplanted mice, showing barely detectable cytokine levels, reaching approximately 2 pg/ml in a fraction of mice (see Reviewer 1 point 5). The data is shown in the New Fig. EV1H. The text has been updated as follows:

"Instead, no significant hematologic abnormalities were observed compared to controls, except a minor T cell reduction in the TNFα group, suggesting specificity of gene expression control by the miRNA-regulated Tie2e/p cassette (EV 1C, E), confirmed by the modest upregulation of IFNγ-responsive genes in the tissues, without altering blood biochemical parameters and with barely detectable IFNγ levels in the plasma of engrafted mice (EV 1F-H)."

Referee #3 (Remarks for Author):

On this manuscript, Adele M., et al. propose an IFN-y gene therapy strategy to manipulate the tumor microenvironment (TME) and restore immune anticancer response. Based on previous studies, the authors genetically manipulate HSPCs to generate IFN-y delivery macrophages which partially reverse the immunosuppressive TME in B-ALL and colorectal cancer models. The authors showed that their system promotes an initial inflammatory environment in the BM detrimental for the leukemia progression. They claimed that such an environment enhance antigen presenting against the tumor and cytotoxic activity of the T-cells. T-cell exhaustion might explain the leukemia relapse.

Immune-based approaches have emerged as an effective treatment option to mitigate tumorigenesis. Therefore, the relevance of this study relies on its potential therapeutic impact. The in vivo experiments are well designed and some results are intriguing. However, there are major concerns on the mechanistic model. The propose therapy is only efficient for a short period of time (17 days). Therefore, a deep understanding on the IFN-y-mediated changes in the microenvironment, a comprehensive mapping of the interactions between leukemic and immune cells and the molecular mechanisms driving treatment susceptibility and relapse would enhance the novelty and the relevance of this study. Regardless, the analysis of the scRNA-seq data is very superficial and the results are poorly presented in the Figures. Functional validation of the findings is lacking. Finally, it is unclear which are the benefits of IFN-Y genetic transfer vs systemic treatment. Taking all these into account, I recommend that the authors address the following concerns before considering the manuscript for publication.

We apologize for the non-comprehensive analysis of our scRNAseq data in the first version of our manuscript. We have made a significant effort to improve the depth and clarity of these data, discovering novel hints with regards to the mechanisms of immune evasion.

Specific Major Points:

1. Authors claimed that the genetic manipulation of HSPCs to generate pro-inflammatory macrophages would be safest than systemic administration:

o Is the efficiency similar? Did the authors compare side by side systemic administration and genetic transfer?

While we have not directly performed side-by-side comparison, we are aware of data comparing systemically administered Interferon-alpha with gene-based delivery through HSPC transduced with the same Tie2-promoter/miRNA-regulated vector backbone (Naldini, personal communication). Gene-based delivery in the context of the Tie2 cassette provided at least similar (if not better) tumor control than systemic administration of an extended release IFNa formulation

optimized for mice, at dramatically reduced systemic cytokine exposure in healthy organs not affected by the tumor.
We agree with the reviewer that it would be interesting to address the activity of systemic IFNg administration. However, based on literature, there are severe side effects to systemic delivery arguing in favor of employing gene therapy as a safer strategy to better regulate and limit its biodistribution (Devane JG et al. A short 2 week dose titration regimen reduces the severity of flu-like symptoms with initial interferon gamma-1b treatment. Curr Med Res Opin. 2014 Jun;30(6):1179-87. Granstein RD et al. The Systemic Administration of Gamma Interferon Inhibits Collagen Synthesis and Acute Inflammation in a Murine Skin Wounding Model. Journal of Investigative Dermatology 1989 93(1):18-27. Hillman GG et al. Inhibition of murine renal carcinoma pulmonary metastases by systemic administration of interferon gamma: mechanism of action and potential for combination with interleukin 4.Clin Cancer Res 1997 (3) (10) 1799-1806). Based on clinical trials from the 90's, systemic administration of IFNg has been discontinued in most oncologic contexts, due to the presence of moderate to severe side effects. Furthermore, IFNg is responsible for many autoimmune disease (Pollard KM, Cauvi DM, Toomey CB, Morris KV, Kono DH. Interferon-γ and systemic autoimmunity. Discov Med. 2013 Sep;16(87):123-31. PMID: 23998448; PMCID: PMC3934799.). Tightly regulated and local expression represents a drastically safer mechanism of delivery.

We have performed some experiments with a broader expression of the IFNg gene by using either a PGK promoter or SP146/gp91phox myeloid specific promoter. Here, both ubiquitous expression and myeloid specific expression led to death of the animals before engraftment (within the first 14 days post-irradiation). Reduced regulation in IFNg expression causes impairment of HSPCs engraftment.

We have added the following sentence to the new version:
"Conversely, transplantation of cells where these cytokines were expressed from a ubiquitous (PGK) or strong myeloid-specific promoter (SP146-gp91) resulted in 100% lethality by day 14 (not shown)."

Moreover, we now compared the expression of IFNg response genes in mice exposed systemically to a single dose of IFNg to mice treated with the gene therapy approach. The data highlights how the gene therapy is more specifically limiting its systemic expression with barely any induction of IFNg-related genes in other organs except the bone marrow suggesting that cytokine release is tightly controlled (New EV 1G). The text has been integrated as follows:

"Instead, no significant hematologic abnormalities were observed compared to controls, except a minor T cell reduction in the TNFα group, suggesting specificity of gene expression control by the miRNA-regulated Tie2e/p cassette (EV 1C, E), confirmed by the modest upregulation of IFNγ-responsive genes in the tissues, without altering blood biochemical parameters and with barely detectable IFNγ levels in the plasma of engrafted mice (EV 1F-H).

o While manipulation of HSPCs might result in normal hematopoiesis under-steady state conditions, the generation of IFN-y delivery macrophages might be detrimental during inflammatory response triggered by infections or chronic inflammation. Thus, are those mice more susceptible to LPS-mediated endotoxin shock?
Based on the reviewer's suggestion, we induced a LPS-mediated endotoxin shock by i.p. injection of 100ug LPS/mouse (the dose was previously tested in wild type mice and resulted close to LD50). Upon challenge, 4 out of 7 CTRL and 5 out of 7 IFNγ animals died showing no statistical difference as measured by contingency test. Serum of the challenged animals was collected and evaluated for cytokine concentration over-time (figure below). IFNγ was increased in our gene therapy group.

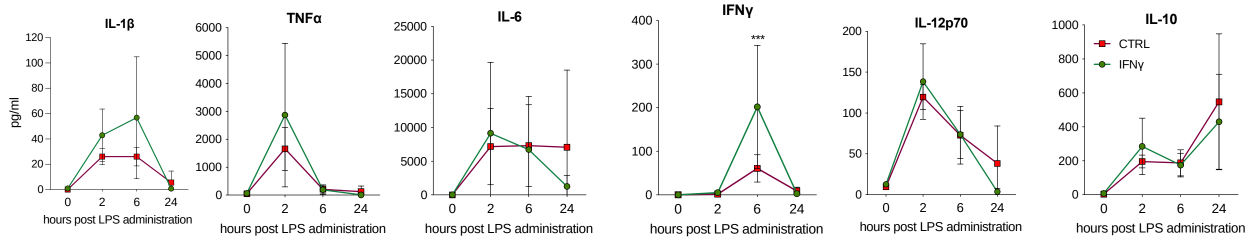

o Supp. Fig 1: Authors claimed that the genetically modified HSPCs (to express IFN-y and TNF-a under the control of Tie2e/p) are able to fully reconstitute hematopoiesis with no major abnormalities (Supp fig. 1C-H). However, the analysis is based in peripheral blood parameters. Quantifications of HSC, MPP and other progenitors in the bone marrow should be included.

We have further characterized the BM environment (New EV 1I) of mice receiving our gene therapy and showed that at steady state, 12 weeks post-transplant, mice with IFNg had higher levels of LSK and MPP cells (New EV 1I). This is probably related to the capacity of type I and II IFN to induce upregulation of Sca-1 (Morcos MNF et al. SCA-1 Expression Level Identifies Quiescent Hematopoietic Stem and Progenitor Cells. Stem Cell Reports. 2017 Jun 6;8(6):1472-1478.; Zhang Y et al. MyD88 Signaling in CD4 T Cells Promotes IFN-γ Production and Hematopoietic Progenitor Cell Expansion in Response to Intracellular Bacterial Infection. The Journal of Immunology May 1, 2013, 190 (9) 4725-4735). The text has been modified as follows:

"For IFNγ, an in-depth toxicity study was performed, confirming that transduced cell engraftment was stably maintained without negative impact on BM progenitor cell numbers (EV 1I). Necropsy with organ histopathology did not reveal abnormalities, except for an incidental finding of thymoma in a single mouse (Appendix Figure S1)."

2. Figure Sup 3: Do the macrophages and CD8 cells in the colorectal model show increased levels of MHC II? Do the colorectal cells also upregulate MHC II?

As shown in the histogram below, we observed similar levels of MHC II between IFNg and CTRL groups within the macrophages in the model of colorectal carcinoma (green lines for IFNg, red lines for CTRL and grey filled for the FMO control, 2 representative animals per group).

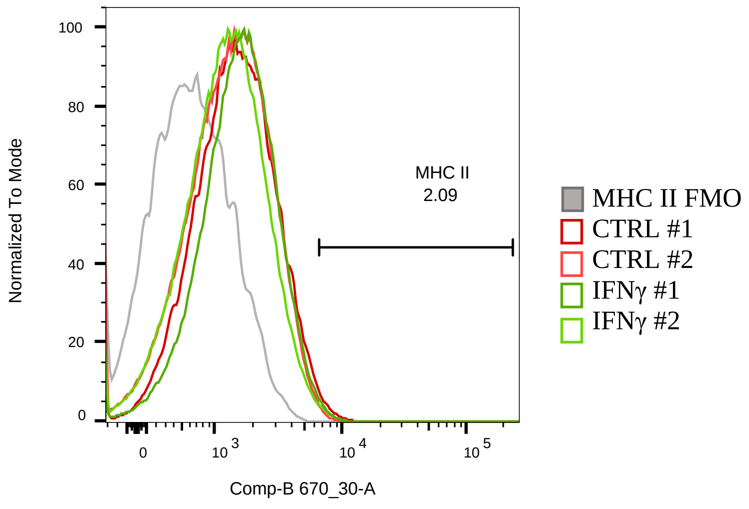

By excluding CD45 positive cells we also looked for MHC II expression on MC38 cells however no expression was detectable (green lines for IFNg, red lines for CTRL and grey filled for the FMO control). This is probably expected for epithelial cells without antigen presenting capacity.

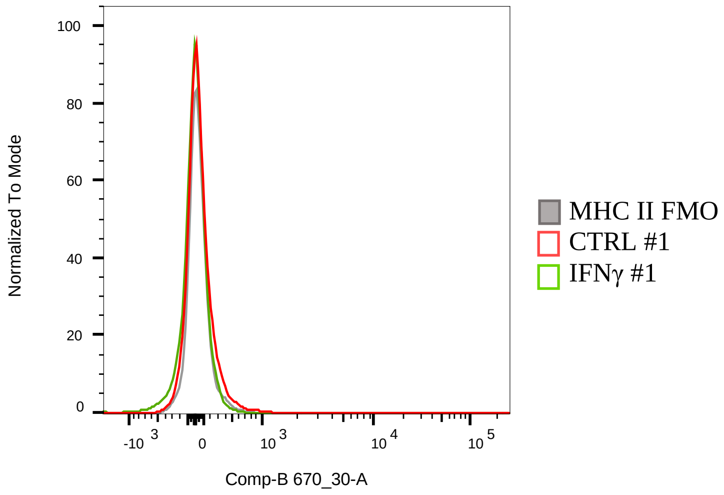

3. Figure 2: A scheme explaining the time line of the different challenges will help to understand the experimental approach.

Following the suggestions from the other reviewers, the data present in Old Fig. 2 have now been moved to Supplementary information (New Fig.5). For clarity, timelines and survival curves have been added. Only a single experiment is shown, as the replicate did not provide additional information.

4. Survival curves in Figures 2, and 6 would strength the data.

As recommended by the Reviewer we included survival curves for the experiment shown in New Fig. EV5. Even though statistical significance is not reached, the trend shows a trend for higher frequency of remaining disease-free after a first challenge with IK6-B-ALL. Unfortunately, we don't have survival data for Figure 6, as all experiments were terminated at the established endpoint, between day16 and 18, to evaluate the changes induced by the different treatments on the tumor microenvironment. We have, however, performed new experiments starting with a therapeutic setup. A B-ALL relapse challenge was performed with survival readout, which is now presented in New Fig. EV4A-E. The manuscript text has been updated as follows:

"To approach a more clinically relevant experimental model, we tested the efficacy of IFNγ gene therapy in a therapeutic setting. Mice challenged with line #11 B-ALL received chemoradiotherapy for disease control and conditioning and were then transplanted with gene-modified lineage-negative HSPCs (Fig. 1H). Importantly, in this therapeutic setting, IFNγ gene therapy resulted in significant leukemia growth inhibition compared to control animals (Fig. 1I). In replicate experiments, where vincristine chemotherapy and irradiation were given earlier after B-ALL injection (EV 4A), most animals were cured from leukemia in both IFNγ and control groups (EV 4B). To model B-ALL relapse, mice surviving the first challenge were then injected with a B-ALL subclone of line #11 (NGFR+/Ovalbumin+). Mice from the IFNγ gene therapy group showed a significant delay in relapse kinetics (EV 4C), which translated into improved clinical condition (EV 4D) and prolonged survival (EV 4E)."

5. Representative FACS plot should be included for the gating strategy of the cells and for the quantification of the levels of MHC II

We have included a new EV Figure (EV 8) where we show examples of flow cytometry analysis with the respective the gating strategies used to define the myeloid and lymphoid compartments in bone marrow and spleen of transplanted animals.

6. The scRNA-seq (Figure 4 and 5) is poorly analyzed. Authors should review some of the

published studies to proper present their results, particularly the bone marrow landscape of B-ALL recently published by Witkowski M. et al., Cancer Cell 2020.

o Figure 4a, b and c: each cluster should properly correlate with a cell type. I recommend the authors to plot: a) UMAP-color coded for unbiased cluster, b) UMAP-color coded for each condition (Control 12, Control 17, IFN-y 12 and IFNy 17) and c) UMAP-color coded for cell calling.
We appreciate the Reviewer's suggestion and have dedicated the New Fig. 3 to cell-type calling, further supported by the New Fig. EV6. The text has been updated as follows:

"Unsupervised clustering identified many transcriptional states representative of the major cell types present in BM, with SingleR classification revealing a broad representation of the different hematopoietic cell types, including B-ALL, myeloid and lymphoid clusters (Fig.3A). In detail, the unsupervised and custom analysis revealed subpopulations within the myeloid (Fig.3B, C) and lymphoid (Fig.3D, E) compartments. Moreover, we detected a small cluster of M2-like macrophages expressing a characteristic TEM signature (EV 6A, B) (Pucci *et al*, 2009), as well as a distinct subset of non-classical monocytes (mHB-M2) that have recently been associated with disease progression in B-ALL (EV 6C, D) (Witkowski *et al*, 2020). Notably, the mHB-M2 subset was overrepresented in IFNγ treated animals at day 17, indicating a possible mechanism of therapy resistance (EV 6D, E)."

o A heatmap or a BubbleMap showing the levels of the main genes used as cell markers should accompanied Figure 4a, 4b and 4c.
Based on the reviewer suggestion we have added this information in the New Fig. 3, which now includes the panels in the recommended order.

o Cluster contribution in each condition should be addressed.
We included in the New Fig. 4A/B the bar-graphs describing cluster contribution to each sample, and here below the heatmap exemplifying the same data.

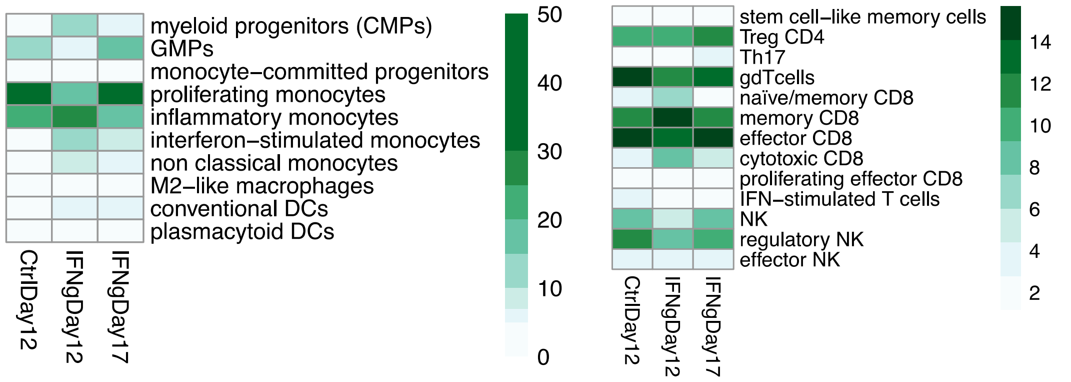

o It is unclear whether B-ALL cells are present in Figure 4a.
The former Figure 4A already included B-ALL cells. However, to further highlight where they fall, we have added to single R definition the rTTA to label B-ALL cells in the New Fig. 5, which are now clearly identifiable.

o Which is the TEM signature include in Figure 4d? A heatmap/BubbleMap should be included
We have moved this information to the New EV Figure 6A/B and have added the heatmap highlighting the genes that characterize TEMs.

o Which is the distribution of the cell types included in Figure 4c per sample? Are there differences between them?

We have included the population distributions in the different samples in the New Figure 4 A and B. However, we didn't observe any major differences, in particular when comparing the d17 IFNg sample with CTRL d12.

The text has been updated as follows:
"Next, we compared the relative representation of the different subpopulations in the sequenced samples. IFNγ-treated animals showed increased myeloid progenitors (CMPs+GMPs), dendritic cells and, surprisingly, non-classical monocytes at the expense of proliferating monocytes (Fig 4A), as well as an increase of cytotoxic CD8 lymphocytes on day 12 (Fig. 4B)."

o What are: interferon stimulated monocytes and inflammatory monocytes? Based on which gene expression patterns the authors are separating these two populations?
We have decided to subdivide the classical monocytes based on the different expression of either proliferation genes, inflammatory genes or specific IFNg-induced genes. As included in the New Fig. 3C, we used marker genes to separate the two populations, even though they still belong to the bigger category of classical monocytes, as now indicated in the Figure.

o Where are the classical monocytes (which is the major population of the bone marrow) in Figure 4c?
As highlighted by expression of Ly6c2 (Figure below), classical monocytes make up most of the myeloid cells in the bone marrow, also in our B-ALL context. In order to better understand the changes induced by our gene therapy approach, however, we subdivided classical monocytes based on proliferation and expression of IFNγ signature, defining inflammatory monocytes, proliferating monocytes and interferon-stimulated monocytes (heatmap with markers of the different populations below and in the New Figure 3C).

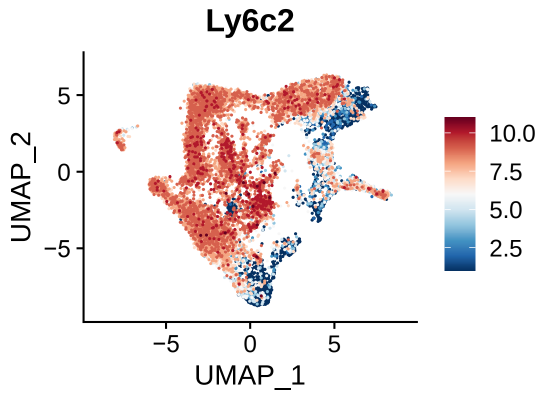

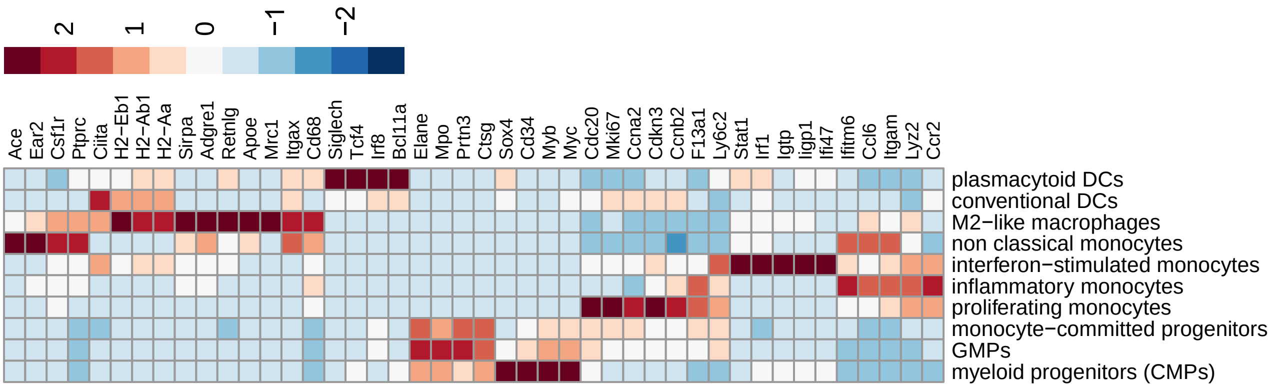

o A subset of Non classical monocytes has been shown to play essential roles in the progression of B-ALL (Witkowski et al., 2020). Can the authors identify these subsets in their study? Do IFN-y

mice show less non-classical monocytes at day 12 than day 17? How do the authors correlate their results with this study?

Yes we can identify them (as shown in the figures below in scRNAseq and included in the New EV Figure 6), and they colocalized with our subset of non-classical monocytes. Our treatment seems to increase this subpopulation, probably as a negative feedback loop induced by IFNg to regulate inflammation. Indeed, IFNg treated mice up-regulate genes of non-classical monocytes (Witkowski et al., 2020) over-time, with lower expression at d12 and increased at d17. This might represent one of the mechanisms employed by leukemia to overcome the immune pressure induced by our gene therapy. Moreover, we analyzed the expression levels of the top genes identified by Witkowski et al. 2020 of murine non-classical monocytes according to their expression at different time points.

The results section has been updated as follows:

"Moreover, we detected a small cluster of M2-like macrophages expressing a characteristic TEM signature (EV 6A, B) (Pucci *et al*, 2009), as well as a distinct subset of non-classical monocytes (mHB-M2) that have recently been associated with disease progression in B-ALL (EV 6C, D) (Witkowski *et al*, 2020). Notably, the mHB-M2 subset was overrepresented in IFNγ treated animals at day 17, indicating a possible mechanism of therapy resistance (EV 6D, E)."

Potential implications of these findings are now discussed:

"Interestingly, TEMs were distinct from a pro-leukemic population of non-classical monocytes described by Witkowski *et al*. This latter population was detected at higher frequency in the IFNγ-treated animals (see EV Fig.6), indicating a potential feedback mechanism activated by leukemia to facilitate immune evasion. It is tempting to speculate that drugs specifically targeting these non-classical monocytes (but not TEMs) may synergize with IFNγ gene therapy in B-ALL and possibly other tumors."

7. Overall, it is unclear the transcriptional and molecular mechanisms by which IFN-y promotes a delay in the leukemia progression at day 12 and how or why leukiemic cells scape. On the one hand the authors claimed that B-ALL cells increase MHCII and suggest that they could become a target from the immune system. However, do the authors observe increased cell death in the RNA-seq or signs of cytotoxic activity? Are there differences in proliferation? On the other hand, the authors claimed that on d17 the B-ALL cells become more oncogenic (upregulation of Myc, Kit...etc). Is the IFN-y signaling directly delaying the oncogenic transformation?

Based on flow cytometry and scRNAseq data, we observe that B-ALL has higher viability and proliferation in CTRL versus IFNg (as shown by Annexin V staining and Ki67 expression – below image). These data are now shown in the New Fig. 5.

"Moreover, B-ALL cells from IFNγ treated animals displayed decreased viability and reduced expression of the proliferation marker Mki67 compared to controls (Fig. 5E, F)."

However, direct involvement of IFNg in B-ALL transformation could only be studied by generating an IFNg insensitive disease. We attempted to obtain B-ALL by inducing over-expression of miR126 in IFNgR1 KO bone marrows, but we were unable to obtain a big enough mouse cohort to obtain a transformation event.

8. Which are the transcriptional differences between B-ALL cells that express high and low MHC II levels?

We performed GSEA analysis showing top hallmark gene sets for MHCII low and MHCII high BALL subsets, as shown below. MHCII-high B-ALL cells displayed gene sets of inflammatory and interferon-related responses, whereas MHCII-low B-ALL cells oxidative phosphorylation, myc targets and ROS pathways. This is an interesting finding, as explained in the response to reviewer 2 point 1 and has been included in the New Figure 5.

"Based on the MHC II molecules expression module score, we divided B-ALL cells from IFNγ d12 into an MHCII-high subpopulation, displaying enrichment for IFN-responsive gene modules and an MHCII-low subpopulation, with higher expression of proliferative and oxidative phosphorylation pathways (Fig. 5G, H). Moreover, at day 17, B-ALL cells from treated animals showed reduced expression of the IFNγ receptors and transducers (Ifngr1, Ifngr2, and Jak1), accompanied by reduced intracellular signaling via Stat1 and Irf1 (Fig 5I), possibly indicating the development of a resistance mechanism to IFNγ (Arenas *et al*, 2018).

9. Are there differences in the percentage of exhausted T cells between the different conditions studied included in the scRNA-seq?
To evaluate T cells, we measured expression of genes associated with exhaustion. Even if only small, there seems to be an increase in T cell exhaustion at the late timepoint of our IFNg gene therapy (Figure below). The module score is based on expression of Pdcd1, Lag3, Tigit, Cd244a, Ctla4, Cd160, Nt5e, Egr2, Eomes, Tcf7, Batf, and Tox. However, this doesn't seem to be the main mechanism used by the leukemia to escape the immune system as only mild differences were observed.

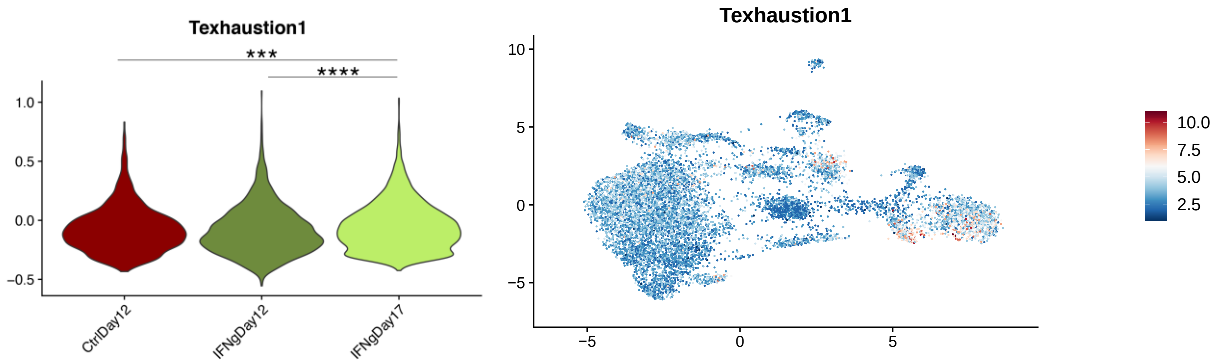

Mapping of exhausted T cells on the UMAP plot is shown in New Fig.4G.

Can authors correlate data on Figure 4b and Figure 5B and C?
We included updated panels in the New Figure 4 for T cells exhaustion and TCR expansion.
"CD4+ T cells and some cytotoxic CD8+ T cells showed expression of T cell exhaustion markers (Fig. 4G), but with barely any differences between the CTRL and IFNγ."

10. Authors should review the writing. Some expressions are inaccurate (f.i. "a subline that models progressed form of leukemia") and many sentences are complicated and difficult to understand. We have copy-edited the text, and hope it reads better now.

Minor:
1. The legend of Figure 1 should clarify the differences in the tumor burden on experiments showed on 1a-b and 1c-d.
2. Representative FACS plots and gating strategy should be included, specially to characterize: Macrophages, Monocytes... Inserted EV Figure 6
3. Figure 2: Survival representation should complement the PB quantification of B-ALL cells. Added to Figure 2
4. Figure 2: The authors claims that 2/3 control mice died from B-ALL (Figure 2B) but the graphs shows 4 control mice. Text editing
5. Peripheral blood numbers of T (CD3+) and B (CD19) cells in Figure 3a doesn't represents normal hematopoiesis. Usually at steady-state hematopoiesis or 4-5 weeks after transplantation the distribution of B-cells is around 60-70% and T-cells below 25%.
6. Statistical analysis should be included in Figure 4e and f.

These minor points have been addressed.

5th Aug 2021

Dear Dr. Gentner,

Thank you for the submission of your revised manuscript to EMBO Molecular Medicine. We have now received the enclosed reports from the referees who had reviewed your original manuscript. As you will see, they are now supportive of publication, and I am therefore pleased to inform you that we will be able to accept your manuscript once the following points will be addressed:

1/ Main manuscript text:
- Please remove the red text and only keep in track changes any new modification.
- Please provide up to 5 keywords.
- Please remove Main points #1 and #2.
- As per our guidelines on "Unpublished Data", the journal does not permit citation of "Data not shown". All data referred to in the paper should be displayed in the main or Expanded View figures.
- Please introduce a space before a reference in parenthesis.
- Material and Methods: when referring to previously published methods, make sure that sufficient information is available to ensure reproducibility of the experiments. Please include the EV supplemental methods in the main manuscript.
o Mice: please indicate the age and gender of the mice used in the experiments. Please indicate the housing and husbandry conditions.
o Human samples: please include the full statement that informed consent was obtained from all subjects and that the experiments conformed to the principles set out in the WMA Declaration of Helsinki and the Department of Health and Human Services Belmont Report.
o Cells: please indicate whether the cells were tested for mycoplasma contamination.
- Thank you for providing a Data Availability section. Please place it after the Material and Methods and note that the data must be publicly available before online publication.
- The Funding information should be merged with the Acknowledgements.
- Please rename the Competing Interests "Conflict of Interest"
- Please rename "The Paper Explained" section.
- References: 10 authors should be listed before et al.

2/ Figures and Appendix:
- A maximum of 5 Expanded View (EV) Figures can be typeset. EV figures should be uploaded individually. EV Figures should be cited as 'Figure EV1, Figure EV2" etc... in the text and their respective legends should be included in the main text after the legends of regular figures. The additional figures should be either main figures, or bundled together with their legends in a single PDF file called *Appendix*, which should start with a short Table of Content. Appendix figures should be referred to in the main text as: "Appendix Figure S1, Appendix Figure S2" etc.
- Please indicate in the main and appendix figures or in their legends the exact p= values, not a range, along with the statistical test used. Please also provide exact p values for non-significant results (n.s.). Some people found that to keep the figures clear, providing a supplemental table with all exact p-values was preferable. You are welcome to do this if you want to.
- Please add legends to your dataset EV files. Tables EV1 and EV2 could be placed in the Appendix. Table EV2: please also provide the antibody dilutions.
- Please add a Table of Content to the Appendix.
- Please make sure that all figures are referenced in the main text. Callouts are currently missing for

all panels of Figures EV 1-3, 5-8, Tables EV 1,3,4 (callouts for Table 3, 4).

3/ We would also encourage you to include the source data for figure panels that show essential data. Numerical data should be provided as individual .xls or .csv files (including a tab describing the data). For blots or microscopy, uncropped images should be submitted (using a zip archive if multiple images need to be supplied for one panel). Additional information on source data and instruction on how to label the files are available at
.

4/ Checklist:
- section C/7: indicate whether the cells were tested for mycoplasma contamination.
- section E/12: include the full statement that informed consent was obtained from all subjects and that the experiments conformed to the principles set out in the WMA Declaration of Helsinki and the Department of Health and Human Services Belmont Report.
- sections F/19-20-21 and G/22: please fill.

5/ For more information: There is space at the end of each article to list relevant web links for further consultation by our readers. Could you identify some relevant ones and provide such information as well? Some examples are patient associations, relevant databases, OMIM/proteins/genes links, author's websites, etc...

6/ Thank you for providing a nice synopsis image. I have resized it and cropped part of it for the eTOC, please let me know if you agree with both (attached) as these would be the final versions and changes during proofing are usually not allowed.
I also slightly edited your synopsis text, please let me know if you agree with the following, or amend as you see fit:

IFNγ gene therapy is safe and reduces tumor progression in mouse models of B-cell acute lymphoblastic leukemia and colorectal carcinoma. Its effects are immune-mediated through antigen-presentation and reprogramming of the tumor microenvironment.
- IFNγ antitumor effects are driven by immune activation through antigen presentation, clonal T cell expansion, and TME reprogramming
- Combining IFNγ gene therapy with other immunotherapies leads to enhanced and prolonged anti-tumoral activity

7/ As part of the EMBO Publications transparent editorial process initiative (see our Editorial at http://embomolmed.embopress.org/content/2/9/329), EMBO Molecular Medicine will publish online a Review Process File (RPF) to accompany accepted manuscripts.
This file will be published in conjunction with your paper and will include the anonymous referee reports, your point-by-point response and all pertinent correspondence relating to the manuscript.
Let us know whether you agree with the publication of the RPF and as here, if you want to remove or not any figures from it prior to publication.
Please note that the Authors checklist will be published at the end of the RPF.

I look forward to receiving your revised manuscript.

Yours sincerely,

Lise Roth

Lise Roth, PhD
Editor
EMBO Molecular Medicine

To submit your manuscript, please follow this link:

Link Not Available

*Additional important information regarding Figures
Each figure should be given in a separate file and should have the following resolution:
Graphs 800-1,200 DPI
Photos 400-800 DPI
Colour (only CMYK) 300-400 DPI"

Figures are not edited by the production team. All lettering should be the same size and style; figure panels should be indicated by capital letters (A, B, C etc). Gridlines are not allowed except for log plots. Figures should be numbered in the order of their appearance in the text with Arabic numerals. Each Figure must have a separate legend and a caption is needed for each panel.

*Additional important information regarding figures and illustrations can be found at https://bit.ly/EMBOPressFigurePreparationGuideline

The system will prompt you to fill in your funding and payment information. This will allow Wiley to send you a quote for the article processing charge (APC) in case of acceptance. This quote takes into account any reduction or fee waivers that you may be eligible for. Authors do not need to pay any fees before their manuscript is accepted and transferred to our publisher.

***** Reviewer's comments *****

Referee #1 (Remarks for Author):

The authors have addressed my concerns and points.

Referee #2 (Remarks for Author):

The authors have done a good job in addressing my concerns

Referee #3 (Remarks for Author):

The authors have performed many additional experiments and clarified all my concerns. I have no

further comments and gladly recommend the publication of this work.

The authors performed the requested editorial changes.

2nd Revision - Editorial Decision 12th Aug 2021

12th Aug 2021

Dear Dr. Gentner,

Thank you for submitting your revised manuscript to EMBO Molecular Medicine. We are pleased to inform you that your manuscript is accepted for publication and is now being sent to our publisher to be included in the next available issue of EMBO Molecular Medicine!

Please note that all data have to be publicly available before online publication.

Please read below for additional IMPORTANT information regarding your article, its publication and the production process.

Congratulations on your interesting work!

Lise Roth

Lise Roth, Ph.D
Editor
EMBO Molecular Medicine

Follow us on Twitter @EmboMolMed
Sign up for eTOCs at embopress.org/alertsfeeds

EMBO PRESS

YOU MUST COMPLETE ALL CELLS WITH A PINK BACKGROUND

PLEASE NOTE THAT THIS CHECKLIST WILL BE PUBLISHED ALONGSIDE YOUR PAPER

Corresponding Author Name: Bernhard Gentner
Journal Submitted to: EMBO Molecular Medicine
Manuscript Number: EMM-2020-13598-V2

Reporting Checklist For Life Sciences Articles (Rev. June 2017)

This checklist is used to ensure good reporting standards and to improve the reproducibility of published results. These guidelines are consistent with the Principles and Guidelines for Reporting Preclinical Research issued by the NIH in 2014. Please follow the journal’s authorship guidelines in preparing your manuscript.

A- Figures
1. Data
The data shown in figures should satisfy the following conditions:

- the data were obtained and processed according to the field’s best practice and are presented to reflect the results of the experiments in an accurate and unbiased manner.
- figure panels include only data points, measurements or observations that can be compared to each other in a scientifically meaningful way.
- graphs include clearly labeled error bars for independent experiments and sample sizes. Unless justified, error bars should not be shown for technical replicates.
- if n< 5, the individual data points from each experiment should be plotted and any statistical test employed should be justified
- Source Data should be included to report the data underlying graphs. Please follow the guidelines set out in the author ship guidelines on Data Presentation.

2. Captions
Each figure caption should contain the following information, for each panel where they are relevant:

- a specification of the experimental system investigated (eg cell line, species name).
- the assay(s) and method(s) used to carry out the reported observations and measurements
- an explicit mention of the biological and chemical entity(ies) that are being measured.
- an explicit mention of the biological and chemical entity(ies) that are altered/varied/perturbed in a controlled manner.

- the exact sample size (n) for each experimental group/condition, given as a number, not a range;
- a description of the sample collection allowing the reader to understand whether the samples represent technical or biological replicates (including how many animals, litters, cultures, etc.).
- a statement of how many times the experiment shown was independently replicated in the laboratory.
- definitions of statistical methods and measures:
  - common tests, such as t-test (please specify whether paired vs. unpaired), simple χ2 tests, Wilcoxon and Mann-Whitney tests, can be unambiguously identified by name only, but more complex techniques should be described in the methods section;
  - are tests one-sided or two-sided?
  - are there adjustments for multiple comparisons?
  - exact statistical test results, e.g., P values = x but not P values < x;
  - definition of ‘center values’ as median or average;
  - definition of error bars as s.d. or s.e.m.

Any descriptions too long for the figure legend should be included in the methods section and/or with the source data.

In the pink boxes below, please ensure that the answers to the following questions are reported in the manuscript itself. Every question should be answered. If the question is not relevant to your research, please write NA (non applicable). We encourage you to include a specific subsection in the methods section for statistics, reagents, animal models and human subjects.

USEFUL LINKS FOR COMPLETING THIS FORM

http://www.antibodypedia.com
http://1degreebio.org
http://www.equator-network.org/reporting-guidelines/improving-bioscience-research-repor
http://grants.nih.gov/grants/olaw/olaw.htm
http://www.mrc.ac.uk/Ourresearch/Ethicsresearchguidance/Useofanimals/index.htm
http://ClinicalTrials.gov
http://www.consort-statement.org
http://www.consort-statement.org/checklists/view/32-consort/66-title
http://www.equator-network.org/reporting-guidelines/reporting-recommendations-for-tumo
http://datadryad.org
http://figshare.com
http://www.ncbi.nlm.nih.gov/gap
http://www.ebi.ac.uk/ega
http://biomodels.net/
http://biomodels.net/miriam/
http://jjj.biochem.sun.ac.za
https://osp.od.nih.gov/biosafety-biosecurity-and-emerging-biotechnology/
http://www.selectagents.gov/

B- Statistics and general methods

Please fill out these boxes (Do not worry if you cannot see all your text once you press return)

1.a. How was the sample size chosen to ensure adequate power to detect a pre-specified effect size?	A minimum of two technical replicates was always included and most experiments were performed in at least two experimental duplicates.
1.b. For animal studies, include a statement about sample size estimate even if no statistical methods were used.	The sample size was decided based on each experiment in using a statistical calculation with a potency of 0.8 based on expected means and standard deviations. In general we always had a minimum of 4 animals/group in each experiment and experiments were repeated at least twice to confirm the observed effects.
2. Describe inclusion/exclusion criteria if samples or animals were excluded from the analysis. Were the criteria pre-established?	If animals or data points were excluded, the values would have resulted positive in the Outlier Test included in the PRISM software.
3. Were any steps taken to minimize the effects of subjective bias when allocating animals/samples to treatment (e.g. randomization procedure)? If yes, please describe.	MC38 tumors measurments were performed in blind by hiding the cage card from the person taking the measurments. Bioluminescence imagine has been performed with unbiased software analysis/calibration.
For animal studies, include a statement about randomization even if no randomization was used.	In figure EV4, mice were randomized in the groups to achieve similar average leukemia burden based on bioluminescence data.
4.a. Were any steps taken to minimize the effects of subjective bias during group allocation or/and when assessing results (e.g. blinding of the investigator)? If yes please describe.	For MC38 caliber measurments and colony unit counts, the investigator was blinded. Otherwise, most of the others readouts could not be biased from the investigator.
4.b. For animal studies, include a statement about blinding even if no blinding was done	Blinding was performed in the MC38 context by hiding the cage cards from the investigator taking the measurments.
5. For every figure, are statistical tests justified as appropriate?	Statistical tests applied to each figure are explained in the figure description and have been performed with PRISM or R. Statistical analysis performed on single cell RNA seq data was performed and described in the supplementary methods.
Do the data meet the assumptions of the tests (e.g., normal distribution)? Describe any methods used to assess it.	If a parametric test was used, data was first tested for normal distribution. Otherwise, a non-parametric test was employed.
Is there an estimate of variation within each group of data?	Yes, shown in every figure as mean +/- standard deviation

Is the variance similar between the groups that are being statistically compared?	Yes

C- Reagents

6. To show that antibodies were profiled for use in the system under study (assay and species), provide a citation, catalog number and/or clone number, supplementary information or reference to an antibody validation profile. e.g., Antibodypedia (see link list at top right), 1DegreeBio (see link list at top right).	All detailes on used antibodies can be found in EV Table 2
7. Identify the source of cell lines and report if they were recently authenticated (e.g., by STR profiling) and tested for mycoplasma contamination.	B-ALL leukemias were obtained from in vivo expansion of the originally generated diseases (Escobar et al. 2018). These cells do not grow in culture. In the absence of exposure to a cell culture environment, mycoplasma screening has not been regarded useful.

* for all hyperlinks, please see the table at the top right of the document

D- Animal Models

8. Report species, strain, gender, age of animals and genetic modification status where applicable. Please detail housing and husbandry conditions and the source of animals.	Mouse - C57Bl/6 and C57Bl/6 Ly5.1: purchased from Charles River -females - 8 weeks at time of treatment (transplant of genetically modified HSCPs) if not differently specified in the experimental outline. Mouse - NSG W41: internal breeding at the mouse facility - 6-8 weeks at time of treatment (challenge with human B-ALL cells). Housing was in the two animal facilities of the San Raffaele Hospital (Dibit 1 - Via Olgettina 58, 20132 Milan, Italy and Dibit2 -Via Olgettina 60, 20132 Milan, Italy). Mice were housed with a limit of 5 animals/cage, were fed at libitum and light/dark cycles were controlled.
9. For experiments involving live vertebrates, include a statement of compliance with ethical regulations and identify the committee(s) approving the experiments.	All animal procedures were performed according to protocols approved by the Animal Care and Use Committee of the San Raffaele Scientific Institute (IACUC 600, 836, 936, 1095 and 1102) and communicated to the Ministry of Health and local authorities according to italian law.
10. We recommend consulting the ARRIVE guidelines (see link list at top right) (PLoS Biol. 8(6), e1000412, 2010) to ensure that other relevant aspects of animal studies are adequately reported. See author guidelines, under 'Reporting Guidelines'. See also: NIH (see link list at top right) and MRC (see link list at top right) recommendations. Please confirm compliance.	Confirm Compliance

E- Human Subjects

11. Identify the committee(s) approving the study protocol.	San Raffaele Hospital Institutional Review Board
12. Include a statement confirming that informed consent was obtained from all subjects and that the experiments conformed to the principles set out in the WMA Declaration of Helsinki and the Department of Health and Human Services Belmont Report.	All primary cells were obtained from donors that signed informed consent forms approved by the Ospedale San Raffaele Ethics Committee, in accordance with the declaration of Helsinki, the Department of Health and Human Services Belmont Report and the Good Clinical Practice guidelines of the International Conference on Harmonization
13. For publication of patient photos, include a statement confirming that consent to publish was obtained.	not applicable
14. Report any restrictions on the availability (and/or on the use) of human data or samples.	not applicable
15. Report the clinical trial registration number (at ClinicalTrials.gov or equivalent), where applicable.	not applicable
16. For phase II and III randomized controlled trials, please refer to the CONSORT flow diagram (see link list at top right) and submit the CONSORT checklist (see link list at top right) with your submission. See author guidelines, under 'Reporting Guidelines'. Please confirm you have submitted this list.	not applicable
17. For tumor marker prognostic studies, we recommend that you follow the REMARK reporting guidelines (see link list at top right). See author guidelines, under 'Reporting Guidelines'. Please confirm you have followed these guidelines.	not applicable

F- Data Accessibility

18: Provide a "Data Availability" section at the end of the Materials & Methods, listing the accession codes for data generated in this study and deposited in a public database (e.g. RNA-Seq data: Gene Expression Omnibus GSE39462, Proteomics data: PRIDE PXD000208 etc.) Please refer to our author guidelines for 'Data Deposition'.<br><br>Data deposition in a public repository is mandatory for:<br>a. Protein, DNA and RNA sequences<br>b. Macromolecular structures<br>c. Crystallographic data for small molecules<br>d. Functional genomics data<br>e. Proteomics and molecular interactions	A data availability section is included at the end of the Material & Methods section, listing all necessary information to access the files
19. Deposition is strongly recommended for any datasets that are central and integral to the study; please consider the journal's data policy. If no structured public repository exists for a given data type, we encourage the provision of datasets in the manuscript as a Supplementary Document (see author guidelines under 'Expanded View' or in unstructured repositories such as Dryad (see link list at top right) or Figshare (see link list at top right).	We have deposited the single cell RNA sequencing data under the identifier GSE178941. Data will be publicly released once the manuscript has been formally accepted.
20. Access to human clinical and genomic datasets should be provided with as few restrictions as possible while respecting ethical obligations to the patients and relevant medical and legal issues. If practically possible and compatible with the individual consent agreement used in the study, such data should be deposited in one of the major public access-controlled repositories such as dbGAP (see link list at top right) or EGA (see link list at top right).	The study is mostly done in mouse models, and only cells from healthy volunteer human donors are used in some experiments. This manuscript does not refer to any clinical patient data
21. Computational models that are central and integral to a study should be shared without restrictions and provided in a machine-readable form. The relevant accession numbers or links should be provided. When possible, standardized format (SBML, CellML) should be used instead of scripts (e.g. MATLAB). Authors are strongly encouraged to follow the MIRIAM guidelines (see link list at top right) and deposit their model in a public database such as Biomodels (see link list at top right) or JWS Online (see link list at top right). If computer source code is provided with the paper, it should be deposited in a public repository or included in supplementary information.	Detailed information on single cell RNA sequencing data analysis is provided in the methods section.

G- Dual use research of concern

22. Could your study fall under dual use research restrictions? Please check biosecurity documents (see link list at top right) and list of select agents and toxins (APHIS/CDC) (see link list at top right). According to our biosecurity guidelines, provide a statement only if it could.	Our study does not fall under dual use research restrictions.
